# Generative AI use before medical visits: disclosure-item responses, trust, and care-seeking behaviors in a cross-sectional social-media survey in Poland

**DOI:** 10.3389/fdgth.2026.1933451

**Published:** 2026-08-27

**Authors:** Simona Wójcik, Anna Rulkiewicz, Justyna Domienik-Karłowicz

**Affiliations:** 1 Department of Science, LUX MED Sp. z o.o., Warsaw, Poland; 2 LUX MED Sp. z o.o., Warsaw, Poland; 3 Wyższa Szkoła Nauk Medycznych w Warszawie, Warsaw, Poland

**Keywords:** care-seeking behavior, digital health, disclosure-item response, generative artificial intelligence, health information seeking, patient-physician communication, Poland, trust

## Abstract

**Background:**

Patient-facing generative artificial intelligence (AI) tools such as ChatGPT are increasingly used for health information seeking, yet their role in patient–physician communication remains insufficiently characterized. Using the Network Episode Model as the primary framework, this study examined self-reported pre-consultation AI use, whether respondents reported disclosing that use to a physician, and self-reported AI-associated health-behavior change in a convenience sample of Polish adults recruited via social media. Findings are exploratory.

**Methods:**

Cross-sectional online survey, anonymous with respect to the investigators (*N* = 1,044), administered in Polish via Google Forms with programmed routing; recruitment used a multi-account social-media strategy. A 24-item *de novo* questionnaire assessed health-related and pre-consultation AI use, disclosure-item responses, trust, self-reported behavioral consequences, and sociodemographics. Q13 responses were analyzed among respondents reporting AI use before a medical visit; the survey did not confirm that a consultation subsequently occurred. Exploratory analyses included chi-square, Mann–Whitney U, Kruskal–Wallis, Spearman correlations, and logistic regression restricted to definite Yes/No responses; the originally submitted coding and Benjamini–Hochberg adjustment were retained as sensitivity analyses.

**Results:**

Health-related AI use was reported by 84.3% (*n* = 880; 95% CI 82.0–86.4). Among respondents reporting AI use before a medical visit (*n* = 519), 82.5% (*n* = 428; 95% CI 79.0–85.5) selected ‘No’ when asked whether they had informed a physician about that use. Because attendance was not confirmed, this proportion cannot be interpreted as a rate of what patients withheld during completed clinical encounters. Selection of ‘No’ was similar across visit-avoidance strata (79.2–86.8%). Among those selecting ‘No’ who reported no AI-related visit avoidance (*n* = 235), the most frequent reasons were fear of a negative physician reaction (86.4%) and reluctance to undermine physician authority (74.9%). In primary definite-response models, male gender and greater AI trust were associated with the outcomes; AI trust was strongly negatively associated with age. Apparent discrimination and explained variation were modest.

**Conclusions:**

Most respondents reporting pre-consultation AI use selected ‘No’ on the disclosure item. Because consultation attendance and a defined index episode were not confirmed, the actual disclosure rate within completed clinical encounters cannot be estimated from this survey. The findings advance a hypothesis about a possible communication barrier requiring prospective, episode-based study.

## 1 Introduction

Conversational AI tools such as ChatGPT, Gemini, Claude, Grok, DeepSeek, and Copilot are increasingly used for symptom interpretation, medication information, and triage-related questions (1, 2). In a nationally representative Australian survey, approximately one in ten adults reported using ChatGPT to obtain health information in 2024 (3), while multinational survey data indicate growing public engagement with AI chatbots for healthcare assistance across diverse settings (4). From the patient perspective, generative AI is accessible and supportive, though users often fail to recognize its limitations (5). Unlike static health portals, large language models (LLMs) hold a conversation, adapt to context, and respond in a register that is hard to evaluate critically (6, 7). Previous work has framed GPT-4 and related LLMs as potentially useful across medical documentation, education, consultation support, triage, and virtual-assistant functions, while emphasizing that such systems cannot replace direct physician–patient contact or clinical responsibility (8). The implications for care-seeking behavior and the patient–physician relationship remain insufficiently characterized, particularly in Central-Eastern European healthcare contexts; the absence of regulatory oversight for patient-facing LLM health applications has been identified as a systemic vulnerability (9).

In Poland, healthcare professionals report encountering AI-informed patients but describe limited guidance for managing these interactions (10). Polish evidence on generative AI in medicine has so far focused mainly on professional and educational contexts, whereas patient-facing pre-consultation use remains less well characterized (11).

**Figure 1:**
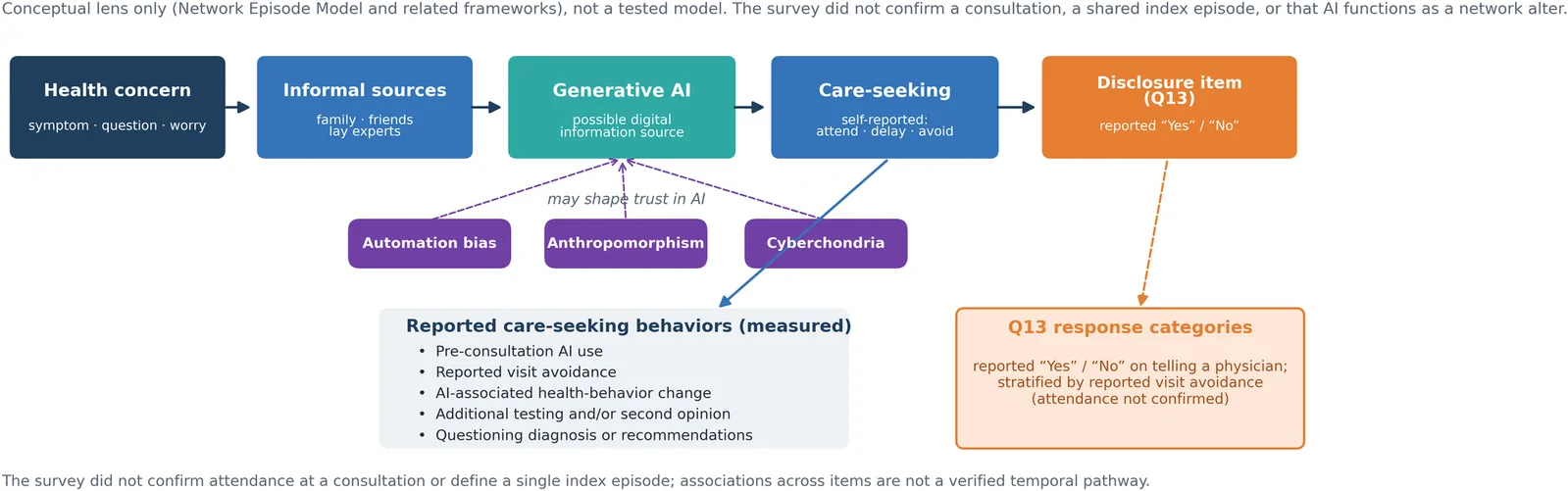
Interpretive framework: generative AI as a possible pre-consultation information source. Conceptual lens only (Network Episode Model and related frameworks); not a tested model. The survey did not confirm a consultation, a shared index episode, or that AI functions as a network alter.

Several established frameworks offer an interpretive lens for these developments. Pescosolido's Network Episode Model describes healthcare utilization as a dynamic social process shaped by informal and professional network ties consulted before, during, or instead of formal care (12). A more recent empirical application similarly demonstrates that the structure and strength of personal-network ties can shape pathways of response to health problems (13). Within this interpretive framework, generative AI may be considered an additional digital information source within the patient's pre-consultation network (Figure 1). However, because the present questionnaire did not define a single index consultation or establish that the relevant items referred to the same event, the study does not test a complete or internally coherent network episode. Automation bias describes over-reliance on automated outputs presented with confidence or apparent technical authority (14, 15). Anthropomorphism—the attribution of human-like qualities to non-human entities (16)—is intensified by conversational AI through natural language and empathetic phrasing, which helps explain why trust in AI-generated health information is clinically relevant (17); generative AI reshapes trust in health communication by shifting both the source of information and the authority attached to it (18). Finally, cyberchondria describes anxiety-amplifying patterns of online health information seeking in which repeated searching increases rather than resolves health concerns (19, 20); conversational AI could either mitigate or intensify this process (21). These frameworks are used to interpret, not to test, the observed behaviors: this study measures self-reported behaviors only, none of these constructs was assessed with a validated psychometric scale, and no direct test of these theories is offered. The Network Episode Model serves as the primary organizing framework for the study; automation bias, anthropomorphism, and cyberchondria are presented only as alternative interpretive concepts that may be compatible with selected observations but were neither operationalized nor tested against competing explanations.

For this study, the primary descriptive outcome was the full Q13 response distribution among respondents reporting AI use before a medical visit (Q4 = Yes); selection of ‘No’ is reported as an item response and not as verified non-disclosure during a completed encounter; the response is referred to throughout as selection of ‘No’ on Q13, without implying that a subsequent consultation occurred, that an opportunity to disclose arose, or that Q4 and Q13 referred to the same episode. Q6 was used as a descriptive stratifier for Q13 and was also examined as a separate exploratory outcome, and Q11 was a second exploratory outcome. In this study, reported visit avoidance and self-reported health-behavior changes (such as medication-related decisions) are treated only as *possible* indicators of AI-associated health-behavior change, not as evidence that professional care was clinically necessary, that avoidance was harmful, or that medication changes involved prescription therapy for chronic conditions. The multivariable models report these two outcomes under neutral behavioral labels (reported visit avoidance; any AI-associated health-behavior change), and the heterogeneous Q11 outcome is not equated with substitution of professional care. Respondents who reported pre-visit AI use and selected ‘No’ on the Q13 disclosure item are described in those literal terms; no label implying a confirmed consultation is used.

The objectives were to: (1) describe the prevalence and patterns of health-related AI use; (2) describe the distribution of Q13 responses (whether respondents reported having told a physician about their AI use) among pre-consultation AI users, including the response distribution by reported visit-avoidance status; (3) characterize self-reported behavioral consequences across three axes—accelerated contact or earlier scheduling, anxiety, and behavior change/testing; and (4) examine gender-associated differences. Analyses were exploratory; no *a priori* hypotheses were preregistered.

## 2 Materials and methods

The study was reported with reference to the STROBE reporting guideline for cross-sectional studies and the CHERRIES checklist for web-based surveys; completed checklists are provided as Supplementary Material, with fields that could not be computed (for example, view rate and participation rate) marked explicitly as not available. The study was not preregistered; all analyses should be considered exploratory.

### 2.1 Study design, setting, and recruitment

This was a cross-sectional online survey conducted in Poland and anonymous with respect to the investigators; Google-account sign-in was required and platform-level data processing by the survey provider remained possible. Participants were recruited between 1 March and 15 April 2026 through an Instagram-based, multi-account dissemination strategy. Adults aged ≥18 years residing in Poland were eligible. The first author published five recruitment invitations on her professional, education-focused Instagram account on 1, 4, 7, 10, and 13 March 2026. The account focused primarily on artificial intelligence and workplace applications; it was not an institutional LUX MED account, and neither the recruitment materials nor the visible profile biography displayed the LUX MED name, logo, or institutional affiliation. One recruitment post was also published on each of the first author's LinkedIn and Facebook accounts on the first day of data collection; because both generated very limited reach, no further posts, sharing, or reposting were undertaken on these platforms.

To broaden dissemination beyond the first author's AI-oriented follower network, the operators of 15 independently managed Instagram accounts were directly asked to share the survey invitation. The accounts represented diverse, predominantly non-medical and non-technical audience profiles, including lifestyle/fashion, beauty and cosmetics, travel, culinary content, fitness and sport, health and wellbeing, parenting and family life, photography and art, and entertainment/humor/memes. Approximate account sizes ranged from 8,000 to 73,000 followers. Further organic redistribution was encouraged, and the invitation was ultimately shared by 101 unique Instagram accounts, including the 15 directly contacted accounts. An anonymized summary of the 15 directly contacted accounts is provided in Supplementary File K.

No paid advertising, boosted post, or other paid promotion was used. Neither respondents nor account owners received financial or non-financial compensation. The recruitment materials described the survey as concerning health-related use of generative AI and the patient–physician relationship but did not disclose the study hypotheses, expected associations, or preferred response patterns. The same Google Forms link was used across all dissemination sources; therefore, the number of completed questionnaires attributable to individual accounts could not be reconstructed. This convenience sampling does not permit generalization to the Polish adult population; the recruitment frame is expected to over-represent digitally active and AI-interested individuals.

Recruitment was conducted independently of LUX MED and outside its healthcare services, facilities, patient-communication channels, and information systems. Participants were members of the public recruited primarily through public Instagram posts and organic sharing by independently managed accounts, with a single additional post on each of the first author's Facebook and LinkedIn accounts on the first day (of very limited reach); they were not recruited from LUX MED patient lists, appointment systems, medical facilities, clinical programs, or employee databases, and no treating physicians, clinical staff, or LUX MED administrative personnel recruited participants on behalf of the study. The sample was not a patient cohort: participants were not recruited or enrolled in their capacity as LUX MED patients, and the survey did not ask about current or previous use of LUX MED services or collect any information identifying respondents as patients of LUX MED or any other healthcare provider. The study did not use LUX MED medical records, patient registries, appointment data, clinical infrastructure, research personnel, marketing databases, mailing lists, social-media channels, or internal analytical systems, and no LUX MED patient or employee identifier was collected or linked to the survey data. The individual-level dataset was controlled by the authors and was not transferred to, stored by, or made accessible to LUX MED for operational, commercial, marketing, employment, or clinical purposes.

No *a priori* sample-size calculation was performed; the study was exploratory and used a convenience sample. For orientation only, a *post hoc* precision assessment indicates that a full-sample proportion estimated on *n* = 1,044 has a maximum 95% confidence-interval half-width of approximately 3.1 percentage points (at *p* = 0.50), and the principal disclosure estimate (*n* = 519) a maximum half-width of approximately 4.3 points (about 3.3 points at the observed proportion). These figures are *post hoc* and do not imply representativeness of Polish adults or adequate power for any routed subgroup or multivariable model. The achieved sample comprised 1,044 submitted questionnaires.

### 2.2 Instrument, consent, routing, platform, and translation

The 24-item questionnaire was developed *de novo*: the authors did not identify a suitable validated Polish instrument that covered the study's combination of pre-visit generative-AI use, disclosure-item responses, and AI-associated care-seeking behaviors. Expert review supported content relevance, clarity, and face validity through review by two independent external experts. The first expert was a medical-communication specialist with a master's degree in psychology and 10 years of professional experience in patient-facing communication, health literacy, and the preparation and review of health-information materials; this expert reviewed the clarity and neutrality of the consultation, medication, diagnostic-testing, health-action, and physician-communication items. The second was a digital-health specialist with a master's degree in economics and 15 years of professional experience in the design, implementation, and usability evaluation of patient-facing digital health services; this expert reviewed the logical sequence, routing, cognitive burden, response-option coverage, and alignment with the intended analyses. Both experts were employed by organizations unaffiliated with the authors, LUX MED, or Wyższa Szkoła Nauk Medycznych, and neither took part in participant recruitment, data collection, statistical analysis, or authorship of the manuscript. Neither expert received any compensation for the review, and neither had a prior professional collaboration with the authors. The instrument was then pretested in two stages with 30 purposively selected adults (age range 18–72 years) who received no compensation and who did not take part in the main survey. In stage 1, 15 participants completed the draft during an online Microsoft Teams session using concurrent verbal probing (sessions lasting approximately 15–25 minutes) to identify comprehension, response-process, option-coverage, and routing problems. In stage 2, a separate group of 15 participants completed the revised questionnaire independently online and then provided structured feedback; estimated unaided completion time was approximately 10 minutes.

Pretesting led to five substantive refinements: (1) examples of generative-AI tools (e.g., ChatGPT, Gemini) were added to the Q1 wording and its introductory text, because some participants were unsure whether an ordinary search engine also counted; (2) ‘don't remember’, ‘not applicable’, and ‘prefer not to answer’ options were added where participants could not give a definite Yes/No answer; (3) requests for additional testing and for a second opinion were distinguished as separate response categories within Q10, because participants treated them as two distinct behaviors; (4) routing was simplified for respondents who did not use AI for health-related matters, so that they were not shown items that did not apply to them; and (5) the Q14 response categories—did not consider it important, fear of a negative physician reaction, no time, physician did not ask, reluctance to undermine physician authority, and ‘other’—were derived from participants' spontaneous comments, with recurring meanings grouped by the authors into the final categories by consensus as informal questionnaire refinement rather than formal qualitative coding. This expert review addressed content relevance, clarity, and face validity and did not constitute a formal content-validity-index exercise or psychometric certification; full details are provided in Supplementary File J. The instrument primarily measured discrete behavioral indicators rather than latent psychometric constructs; therefore, internal-consistency metrics such as Cronbach's alpha were not applicable, and no formal psychometric validation was performed.

The survey was administered to participants exclusively in Polish; the English text was never displayed to respondents. An author-prepared English reporting translation of the questionnaire was produced after data collection, solely for inclusion in the Supplementary Materials and to enable reviewers and international readers to understand the instrument. It was not used for recruitment or data collection and was prepared without generative AI: AR prepared the English reporting translation and JD-K independently cross-checked it against the Polish fielded instrument. Because it was prepared for reporting purposes only, no formal cross-cultural adaptation, back-translation, or psychometric validation of the English version was performed.

Before entering the questionnaire, participants viewed a multi-page Polish-language participant-information module describing the study purpose, procedures, voluntariness, anonymity with respect to the investigators, potential discomfort, data handling, and contact details. Eligibility was confirmed through required declarations that the respondent was aged at least 18 years and lived in Poland. Participants were required to confirm that they had read and understood the study information, understood that participation was voluntary, knew that they could discontinue before submitting the form, understood that withdrawal after anonymous submission might not be possible, and consented to participation and analysis of their responses. A required binary consent item was also used; selecting non-consent terminated the survey. The participant-information, eligibility, and digital-consent screens are reproduced as Supplementary File I.

The survey was administered using Google Forms and was accessible through a responder link. The form was configured to limit submissions to one response per Google account; respondents were therefore required to sign in to a Google account. Collection of email addresses was disabled, and no names or email addresses were included in the research dataset. Respondents could review and revise their answers within the form before final submission; editing after submission was disabled. These response settings, the consent gate, and the implemented branching rules for all four routing items (Q1, Q4, Q11, Q13) are documented by screenshots of the form configuration reproduced in Supplementary File I-b; the corresponding author attests that they were unchanged during data collection from 1 March to 15 April 2026. The configured branching corresponds to the routing specification in Supplementary File P and to the structural non-display patterns in the complete response export. This mechanism limited duplicate participation at the Google-account level but could not completely exclude multiple submissions by the same individual using different accounts. No names, email addresses, national identification numbers, contact details, residential addresses, or medical-record data were collected in or available through the research dataset. Only submitted questionnaires were included in the analytic dataset; the number of incomplete (started but not submitted) sessions was not recorded in the platform export and, together with the view rate and participation rate, is reported as not available in the CHERRIES checklist.

Programmed routing is shown in Supplementary Figure S1 and specified item-by-item in Supplementary File P. Respondents selecting ‘No’ or ‘Don't know/don't remember’ on Q1 skipped Q2–Q14 and proceeded directly to Q15; among respondents selecting ‘Yes' on Q1, Q4 controlled the display of Q5–Q9, Q11 controlled the display of Q12, and Q13 = No triggered Q14. Q15–Q24 were displayed to all respondents. The complete raw export was audited: all 164 respondents who did not report health-related AI use (Q1 = No or Don't know/don't remember) had Q2–Q14 structurally not displayed and completed Q15–Q24; no routing violations were identified. The instruction in the earlier supplementary questionnaire directing Q1 non-users to Q18 was a documentation error; the implemented route was Q15, as documented by the form-configuration screenshots in Supplementary File I-b and verified against the structural non-display patterns in the complete response export. N/D (shown in Supplementary File D) denotes an item not displayed because of programmed routing and is not respondent-level missingness.

### 2.3 Variables and terminology

Item wording is reported using terms that match the actual items. Exact Polish item wording with English translation for the items on which the primary outcome depends (Q6 and Q13) is reproduced here from the questionnaire: Q6 (Polish: ‘Czy zdarzyło się Panu/Pani zrezygnować z wizyty, ponieważ odpowiedź AI zasugerowała, że problem nie wymaga konsultacji?’; English: ‘Have you ever given up a medical visit because the AI response suggested that the problem did not require a consultation?’; response options: Yes/No/I don't remember/Prefer not to answer). Q13 (Polish: ‘Czy powiedział/a Pan/Pani lekarzowi, że korzystał/a z AI w tej sprawie?’; English: ‘Did you tell your physician that you had used AI in this matter?’; response options: Yes/No/I don't remember/Not applicable). Q14, displayed after selection of ‘No’ on Q13, asked: ‘If no—why?’ (multiple answers allowed). The response options were: ‘I did not consider it important,’ ‘I was afraid of a negative physician reaction,’ ‘There was no time,’ ‘The physician did not ask,’ ‘I did not want to undermine the physician's authority,’ and ‘Other.’ The exact original Polish wording and the parallel English translation are reproduced in Supplementary Material S1. The exposure item Q4 asks in Polish about AI use ‘przed wizytą lekarską’ (literally, ‘before a medical visit’), and this literal wording is used throughout the manuscript, tables, and figures. The item does not specify timing, does not confirm that a visit subsequently took place, and does not establish a single index episode shared across items; these limits on interpretation are stated separately (Section 4.6) rather than built into the item label. The literal bilingual wording of every item is provided as Supplementary Material S1 and in the Instrument Fidelity and Routing Matrix (Supplementary File P).

#### 2.3.1 Primary descriptive outcome

The four-category Q13 response distribution (Yes/No/I don't remember/Not applicable) among respondents reporting AI use before a medical visit (Q4 = Yes, *n* = 519). Selection of ‘No’ is reported as an item response and not as verified non-disclosure during a completed encounter.

#### 2.3.2 Secondary exploratory outcomes

Q6, reported giving up a medical visit because an AI response suggested a consultation was unnecessary (Q4 = Yes base); and Q11, any AI-associated health-behavior change without prior physician consultation (Q1 = Yes base, *n* = 880). Q6 was additionally used as a descriptive stratifier in the Q13 analysis and was not treated as evidence that a consultation occurred.

#### 2.3.3 Further descriptively analyzed items

Q9 (self-reported increase in health-related anxiety after AI use, 1–5; Q4 = Yes base). Q8 (perceived preparedness for the physician discussion, 1–5; Q4 = Yes base). Q10 (requests for additional testing and/or a second opinion; Q1 = Yes base), with the two derived outcomes used in Table 4 defined as ‘any additional testing’ = tests only or both, and ‘any second-opinion request’ = second opinion only or both. Q16 (questioning a physician's diagnosis or recommendations after AI use) was displayed to all respondents; because the item is meaningful only for respondents who actually used AI, the Q1 = Yes subset (*n* = 880) is reported as the primary analysis, with the full-sample distribution given as a secondary analysis for completeness. Additional items were analyzed descriptively: Q5, Q7, Q17, and Q18–Q19. Q12 (types of health-behavior change) was a multiple-response item; all categories are reported in Supplementary File D.

#### 2.3.4 Covariates

Self-reported gender (Q22; the survey assessed self-identified gender rather than biological sex, hence the term ‘gender’ is used consistently). Age group (Q21), examined categorically. AI trust (Q15, 1–5). AI use frequency (Q2, 1–4). Digital competency (Q20, 1–5). Education (Q23), examined categorically. Healthcare utilization frequency (Q24, 1–5). Q2 (AI-use frequency) was coded 1–4 from the least to the most frequent option (1 = ‘only once, just to try’; 2 = ‘once or twice’; 3 = 'several times'; 4 = ‘regularly, several times a month or more’), so higher scores denote more frequent use. AI trust (Q15) was anchored 1 = ‘do not trust at all’ to 5 = ‘fully trust’, and digital competency (Q20) 1 = ‘very low’ to 5 = ‘very high’. Healthcare-utilization frequency (Q24) used five categories: 1 = ‘a few times a year or less', 2 = ‘once every few months', 3 = ‘once a month’, 4 = 'several times a month', and 5 = ‘very often’. In the regression models, primary and vocational education were combined into a single lower-education category because only seven respondents reported primary education; this was a revision-stage decision made to avoid an unstable sparse cell.

### 2.4 Statistical analysis

Descriptive statistics are reported with explicit denominators and, for principal proportions, 95% Wilson confidence intervals. Chi-square tests of independence with Cramér's V, Mann–Whitney U with rank-biserial correlation (and the U statistic reported alongside p-values), Kruskal–Wallis H, and Spearman correlations were used for exploratory bivariate analyses. All statistical tests were two-sided with a nominal significance level of *α*=0.05. Cramér's V and rank-biserial correlation are reported as numerical effect sizes; where qualitative descriptors (small/moderate/strong) appear they follow the indicative descriptive conventions in (22) and are not treated as formal decision cut-offs.

For Q6 and Q11, primary binary logistic models were restricted to respondents providing definite Yes/No outcome responses; ‘don't remember’ and ‘prefer not to answer’ responses were excluded rather than coded as confirmed absence. The originally submitted occurrence-versus-no-reported-occurrence coding was retained as a sensitivity analysis. Gender was modeled as man versus woman, with women as the reference category; respondents preferring not to report gender were excluded from gender-stratified and regression analyses. Age and education were modeled categorically after evaluation of the linear ordinal specification. AI trust, AI-use frequency, digital competency, and healthcare-utilization frequency were entered according to the final coding table, with exact coding and reference categories reported. The model specification was logit(outcome) = gender + categorical age + Q15 AI trust + Q2 AI-use frequency + Q20 digital competency + Q24 healthcare utilization + categorical education; no interaction terms were included. The reduction from definite-response eligibility to the final analytic N reflected exclusion of respondents selecting ‘prefer not to answer’ for gender (Q6: 428 to 409; Q11: 792 to 759); the other model covariates were complete in these analytic sets. Model results include the number of observations and events, odds ratios with 95% confidence intervals, Nagelkerke R^2^, Hosmer–Lemeshow calibration, AUC, and FDR-adjusted sensitivity results. Because covariates were measured on different scales, OR magnitudes were not compared as direct measures of relative importance. Variance inflation factors were inspected; the maximum VIF was 3.26 in the Q6 model and 3.01 in the Q11 model, both for AI trust, below conventional thresholds of concern.

Benjamini–Hochberg false discovery rate (FDR) correction was applied across the eight reported gender-stratified comparisons in Table 4 (Q2, Q6, Q9, the Q10 composite, Q10 testing, Q10 second-opinion, Q11, and Q24); all eight remained significant after adjustment. Effect-size signs for Mann–Whitney comparisons follow a women-versus-men convention (positive indicates higher values in women), and binary risk differences are expressed as men minus women. Age and education were entered categorically in the revised analysis at the reviewers' request. Likelihood-ratio tests comparing the categorical specification with a single ordered term were not significant for either covariate (age: Q6 *p* = 0.068, Q11 *p* = 0.92; education, 1 df: Q6 *p* = 0.110, Q11 *p* = 0.052), so the categorical form is used because it was requested at revision rather than as evidence of significant non-linearity. Global likelihood-ratio tests for each multi-level covariate (whole variable versus its omission) are reported in Section 3.7. Data-quality screening. To examine unusual response patterns, a structural response-quality audit of the complete de-identified export verified bidirectional routing and displayed-item completeness, counted exact full-vector matches, and performed a route-aware near-duplicate scan requiring at least 15 jointly displayed fields and at least 95% identity; the age-by-Q15 and Q15-by-Q17 structures were quantified using contingency tables, Cramér's V, entropy, and modal classification accuracy, and broad straightlining across five ordinal items and sequential clustering in export order (lag-1 correlations and permutation-based adjacent-match rates) were assessed. No routing violations were identified. One exact matching pair was found among respondents following the short non-user route; the full dataset was retained and a one-record-removed sensitivity analysis had no substantive effect. Because timestamps, IP addresses, device or user-agent metadata, and source-account identifiers were unavailable, these analyses could not establish respondent identity or exclude automated or multiple participation.

#### 2.4.1 Internal validation

Model performance was internally validated by nonparametric bootstrap resampling, with the full primary model refitted in each resample. For each model, resampling continued until 1,000 valid refits were obtained. The Q6 model required 1,122 sampling attempts; 122 refits were non-convergent or failed numerically. The Q11 model produced 1,000 valid refits in 1,000 attempts. The Q6 optimism-corrected estimates are therefore conditional on successful refitting. Standard optimism correction and a recalibration model were applied, with grouped calibration plots; the full algorithm, random seeds, software versions, and run-level outputs are documented in Supplementary File L and the public repository. Optimism-corrected AUC, Brier score, Nagelkerke R^2^, and calibration slope and intercept are reported as point estimates.

### 2.5 Ethics

Ethical review and approval were not obtained. Under Articles 21 and 29 of the Polish Act of 5 December 1996 on the Professions of Physician and Dentist, mandatory bioethics-committee review applies to medical experiments. The authors assessed the present project as falling outside that statutory category because it was a non-interventional online survey of adults and involved no treatment allocation, diagnostic, therapeutic, or preventive procedure, biological material, medical-record access, recruitment in a clinical setting, or directly identifying data. The investigators received no names, email addresses, contact details, or other direct identifiers. Participants viewed detailed study information, confirmed that they were aged at least 18 years and resided in Poland, and provided informed digital consent before entering the questionnaire. No independent institutional exemption determination was available at resubmission. The study is therefore not described as formally approved or exempt; the authors provide the legal and procedural rationale transparently for editorial assessment. If the editor determines that an independent institutional opinion is required under journal policy, the authors will obtain one before further processing.

## 3 Results

### 3.1 Sample characteristics

Of 1,044 respondents, 572 (54.8%) identified as women, 422 (40.4%) as men, and 50 (4.8%) preferred not to specify their gender. The 25–34 age group was most represented (28.4%), followed by 35–44 (25.4%). Secondary education was most frequent (59.6%, *n* = 622), followed by vocational (21.9%, *n* = 229), higher (17.8%, *n* = 186), and primary education (0.7%, *n* = 7). Mean digital competency was 4.08/5 (SD 0.84). No respondent selected the Q22 ‘Other’ gender category, and no respondent selected ‘Prefer not to answer’ for Q23 education; reporting these zero categories documents the handling of every offered sociodemographic response option. Full demographics are presented in Supplementary Table S1.

Comparison with available Polish benchmarks indicated substantial selection effects. Respondents aged 18–44 accounted for 67.2% of the sample compared with approximately 42.5% of Polish adults, whereas respondents aged ≥65 accounted for 4.6% compared with approximately 25.1%. Secondary education was reported by 59.6% of respondents compared with a harmonized benchmark of approximately 35.1%, whereas higher education was reported by 17.8% compared with approximately 25.2%. These comparisons are descriptive because reference years, age thresholds, and category definitions differed (Supplementary File E); no weighting or formal representativeness test was applied. As a bound on the influence of this selection, a descriptive selection-standardization sensitivity analysis calibrating to available Polish age, sex, and education distributions shifted headline proportions modestly (for example, reported health-related AI use from 84.3% to approximately 79.5–80.8%, and mean AI trust from 3.25 to approximately 2.80–2.94); the education-inclusive calibration was unstable (7 respondents with primary education, effective sample size falling to ∼113) and all such figures are descriptive sensitivity analyses, not representative estimates for Poland (Supplementary File O).

### 3.2 Prevalence and patterns of AI health use

84.3% (*n* = 880; 95% CI 82.0–86.4) reported ever using AI for health-related purposes (Q1 = Yes). Among Q1 = Yes respondents, 40.2% (*n* = 354) reported repeated use, defined as the combination of 'several times' (*n* = 340) and ‘regularly, several times a month or more’ (*n* = 14); the remainder reported occasional use (once or twice: 51.1%, *n* = 450; only once: 8.6%, *n* = 76). The most frequent purposes (Q3, base *n* = 880; a multiple-response item, so percentages do not sum to 100%) were symptom checking (76.0%), evaluating visit necessity (68.9%), and visit preparation (59.2%). 59.0% of Q1 = Yes respondents (*n* = 519; 95% CI 55.7–62.2) reported using AI specifically before a medical visit (Q4 = Yes).

### 3.3 Disclosure among pre-consultation AI users

Because the survey did not confirm whether a medical consultation actually took place following AI use, the disclosure outcome (Q13) is reported as a distribution and stratified by reported AI-related visit avoidance (Q6), not as an encounter-based disclosure estimate.

**Figure 2:**
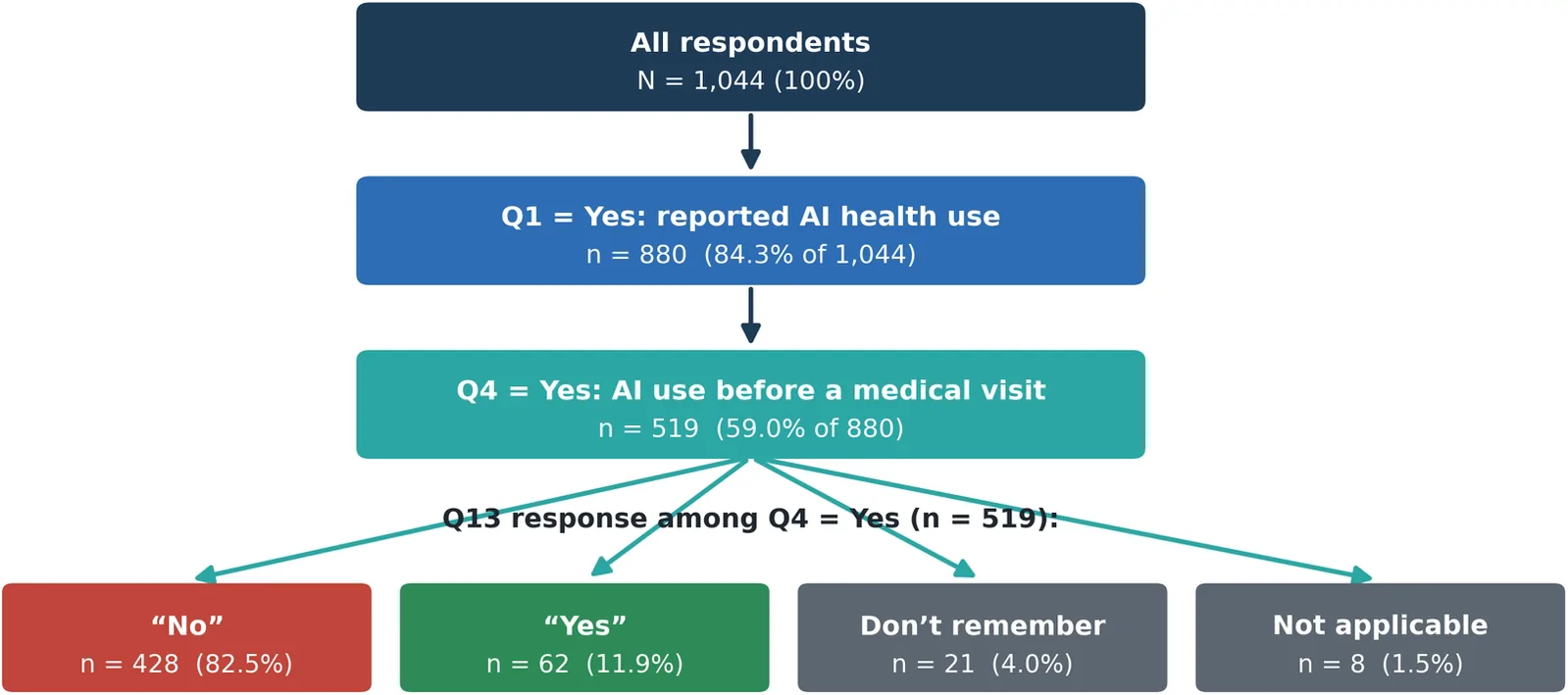
Survey routing and denominator tree. Each node reports n and the percentage of its immediate parent denominator. Q6 (reported visit avoidance) is a stratifier, not a gate to disclosure, and attendance at a consultation was not confirmed; the routing gates are Q1, Q4, Q11, and Q13 (Supplementary Figure S1).

Among all respondents reporting AI use before a medical visit (Q4 = Yes, *n* = 519), 428 (82.5%; 95% CI 79.0–85.5) selected ‘No’ on Q13, 62 (11.9%) selected ‘Yes', 21 (4.0%) selected ‘Don't remember’, and 8 (1.5%) selected ‘Not applicable’ (Table 1). The routing that produced these denominators is shown in Figure 2. In sensitivity analyses, the proportion selecting ‘No’ was 83.8% (428/511) after excluding ‘Not applicable’ responses and 87.3% (428/490) among respondents providing a definite Yes/No answer. This proportion cannot be interpreted as a disclosure rate during completed clinical encounters, because the survey did not confirm that a consultation occurred or that Q6 and Q13 referred to the same episode.

**Table 1:** Disclosure item (Q13) response distribution by reported visit-avoidance status (Q6).

Q6 group	Q13 = Yes (selected)	Q13 = No (selected)	Q13 = No, 95% CI	Don't remember	Not applicable	Row total
Q6 = Yes (reported visit avoidance)	19 (13.2%)	114 (79.2%)	71.8–85.0	8 (5.6%)	3 (2.1%)	144
Q6 = No (no reported AI-related visit avoidance)	32 (11.3%)	235 (82.7%)	77.9–86.7	12 (4.2%)	5 (1.8%)	284
Q6 = Don't remember	5 (11.4%)	39 (88.6%)	76.0–95.0	0 (0.0%)	0 (0.0%)	44
Q6 = Prefer not to answer	6 (12.8%)	40 (85.1%)	72.3–92.6	1 (2.1%)	0 (0.0%)	47
**Total (Q4** **=** **Yes)**	**62** (**11.9%)**	**428** (**82.5%)**	**79.0**–**85.5**	**21** (**4.0%)**	**8** (**1.5%)**	**519**

Base: respondents reporting AI use before a medical visit (Q4 = Yes, *n* = 519). The survey did not confirm that a consultation occurred; cells report the response selected on Q13 and not confirmed clinical disclosure. 95% CI = Wilson interval for the proportion selecting ‘No’ on Q13. All four Q13 response categories are shown within each Q6 group.

'Selected Yes'/'Selected No' denote the response chosen on the disclosure item; because attendance at a consultation was not confirmed and Q6 and Q13 were not tied to the same episode, these are not confirmed clinical disclosure/non-disclosure events. The manuscript's combined ‘Q6 other (*n* = 91)’ category is split here into Don't remember (*n* = 44) and Prefer not to answer (*n* = 47).

Bold values in the final row denote totals across all Q6 response categories, that is, all respondents reporting AI use before a medical visit (Q4 = Yes, *n* = 519).

Stratification by reported visit-avoidance status showed uniformly high selection of ‘No’ across strata: among respondents reporting AI-related visit avoidance (Q6 = Yes, *n* = 144), 79.2% (*n* = 114; 95% CI 71.8–85.0); among respondents reporting no AI-related visit avoidance (Q6 = No, *n* = 284), 82.7% (*n* = 235; 95% CI 77.9–86.7); and among respondents selecting ‘Don’t remember’ or ‘Prefer not to answer’ on Q6 (*n* = 91), 86.8% (*n* = 79; 95% CI 78.4–92.3).

**Figure 3:**
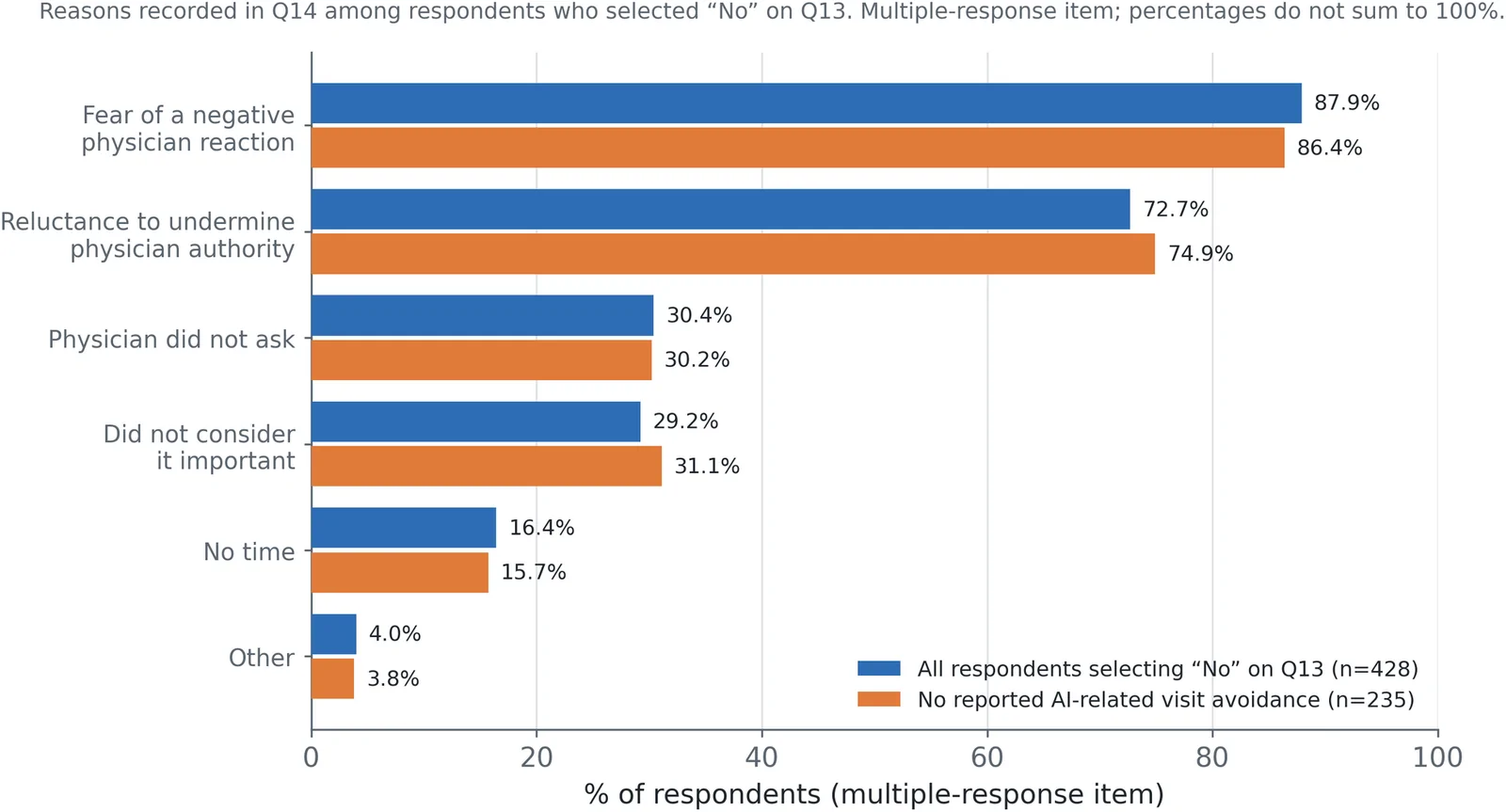
Reasons for selecting ‘No’ on Q13 (disclosure item), recorded in Q14. Multiple-response item; percentages do not sum to 100%. All respondents selecting ‘No’ on Q13 (*n* = 428) and the subgroup reporting no AI-related visit avoidance (*n* = 235).

Among the 428 pre-consultation AI users who selected ‘No’ on Q13, the most frequently selected reasons were fear of a negative physician reaction (Q14.2: 87.9%, *n* = 376), reluctance to undermine physician authority (Q14.5: 72.7%, *n* = 311), physician not asking (Q14.4: 30.4%, *n* = 130), not considering it important (Q14.1: 29.2%, *n* = 125), no time (Q14.3: 16.4%, *n* = 70), and other (Q14.6: 4.0%, *n* = 17). Reasons are presented in Table 2 and Figure 3 (overall, *n* = 428; and restricted to the Q6 = No subgroup, *n* = 235). Q14 was a multiple-response item; percentages do not sum to 100%.

**Table 2:** Self-reported reasons for selecting ‘No’ on the disclosure item (Q14).

Reason	Overall n (%) (*n* = 428)	Overall 95% CI	Subgroup n (%) (*n* = 235)	Subgroup 95% CI
Fear of a negative physician reaction (Q14.2)	376 (87.9%)	84.4–90.6	203 (86.4%)	81.4–90.2
Reluctance to undermine physician authority (Q14.5)	311 (72.7%)	68.3–76.7	176 (74.9%)	69.0–80.0
Physician did not ask (Q14.4)	130 (30.4%)	26.2–34.9	71 (30.2%)	24.7–36.4
Did not consider it important (Q14.1)	125 (29.2%)	25.1–33.7	73 (31.1%)	25.5–37.2
No time (Q14.3)	70 (16.4%)	13.2–20.2	37 (15.7%)	11.6–20.9
Other (Q14.6)	17 (4.0%)	2.5–6.3	9 (3.8%)	2.0–7.1

Q14 was a multiple-response item administered to respondents selecting ‘No’ on Q13; percentages are within-column and do not sum to 100%. Overall column: pre-consultation AI users selecting ‘No’ (Q4 = Yes and Q13 = No, *n* = 428). Subgroup column: those additionally reporting no AI-related visit avoidance (Q4 = Yes, Q6 = No, Q13 = No, *n* = 235). 95% CI = Wilson interval.

The two distributions are closely similar; fear of a negative physician reaction was the most frequently selected reason in both columns, and the ‘physician did not ask’ category was selected by fewer than one third in either column.

### 3.4 Self-reported behavioral responses

The self-reported behavioral responses to pre-consultation AI use are organized along three axes: reported acceleration of contact or earlier scheduling, self-reported anxiety increase, and behavior-change/testing behaviors, with terminology matched to the actual items and no implied validated construct (in particular, no validated patient-activation measure was used).

Reported acceleration of contact or earlier scheduling (base: pre-consultation AI users, Q4 = Yes, *n* = 519). 52.2% (*n* = 271) reported scheduling a medical visit sooner following AI use (Q7). On the perceived-influence item (Q5), 35.5% (*n* = 184) reported that AI information accelerated their decision to contact a physician, 18.7% (*n* = 97) that it delayed the decision, 14.3% (*n* = 74) that they decided not to attend, and 31.6% (*n* = 164) reported no influence. Perceived preparedness for the physician discussion (Q8) had a mean of 3.19/5 (SD 0.72; median 3, IQR 3–4; *n* = 519).

Self-reported anxiety increase (base: pre-consultation AI users, Q4 = Yes, *n* = 519; items Q5–Q9 were administered only to respondents reporting pre-consultation AI use). The item asked whether AI information increased health-related anxiety, so it measures a self-reported change rather than an anxiety level: mean 3.75/5 (SD 0.78; median 4). AI trust was negatively correlated with this anxiety (rs = −0.538, *p* < 0.001).

#### 3.4.1 Self-reported behavior change and testing-related behaviors

Among pre-consultation AI users (*n* = 519), 27.7% (*n* = 144) reported having forgone a visit because an AI response suggested the problem did not require a consultation (Q6). Additional testing and/or second-opinion requests (Q10) were reported by 66.6% (551/827) of AI health users for whom the item was applicable (53 ‘not applicable’ responses excluded from the denominator): additional tests only *n* = 256, second opinion only *n* = 131, and both *n* = 164 (any additional testing *n* = 420; any second opinion *n* = 295). Among all AI health users (*n* = 880), 42.8% (*n* = 377) reported any AI-associated health-behavior change without prior physician consultation (Q11). Uncertain and refusal responses were retained descriptively but excluded from the primary definite-response models rather than coded as absence of the behavior: for Q6 (Q4 = Yes base, *n* = 519), 44 respondents (8.5%) selected ‘don't remember’ and 47 (9.1%) ‘prefer not to answer’; for Q11 (Q1 = Yes base, *n* = 880), 51 (5.8%) selected ‘don't remember’ and 37 (4.2%) ‘prefer not to answer’. Q12 (types of change) was a multiple-response item; the most frequent categories were dietary or supplement changes (57.6%, *n* = 217) and over-the-counter medication use (48.0%, *n* = 181), with self-reported medication cessation reported by 14.9% (*n* = 56); all Q12 categories, including care-escalation categories such as urgent medical attendance, are reported in Supplementary File D. Because Q12 combined lifestyle, medication-related, diagnostic, care-avoidance, and care-escalation behaviors, the broad Q11 outcome should not be interpreted as a single, homogeneous health-behavior outcome, and in particular not as homogeneous substitution of professional care. Q16 (questioning a physician's diagnosis or recommendations after AI use) was displayed to all respondents, but because a respondent who never used AI for health could not have questioned a physician on the basis of AI, the primary analysis is restricted to AI health users (Q1 = Yes, *n* = 880): 436 (49.5%) reported doing so, 368 (41.8%) did not, 52 (5.9%) did not remember, and 24 (2.7%) selected ‘not applicable’. For completeness, the full-sample distribution (*N* = 1,044) was Yes 436 (41.8%), No 447 (42.8%), Don't remember 52 (5.0%), and Not applicable 109 (10.4%). None of the 164 respondents without health-related AI use gave an affirmative response, so restricting the base to AI users omits no affirmative case.

### 3.5 AI trust by age group

**Figure 4:**
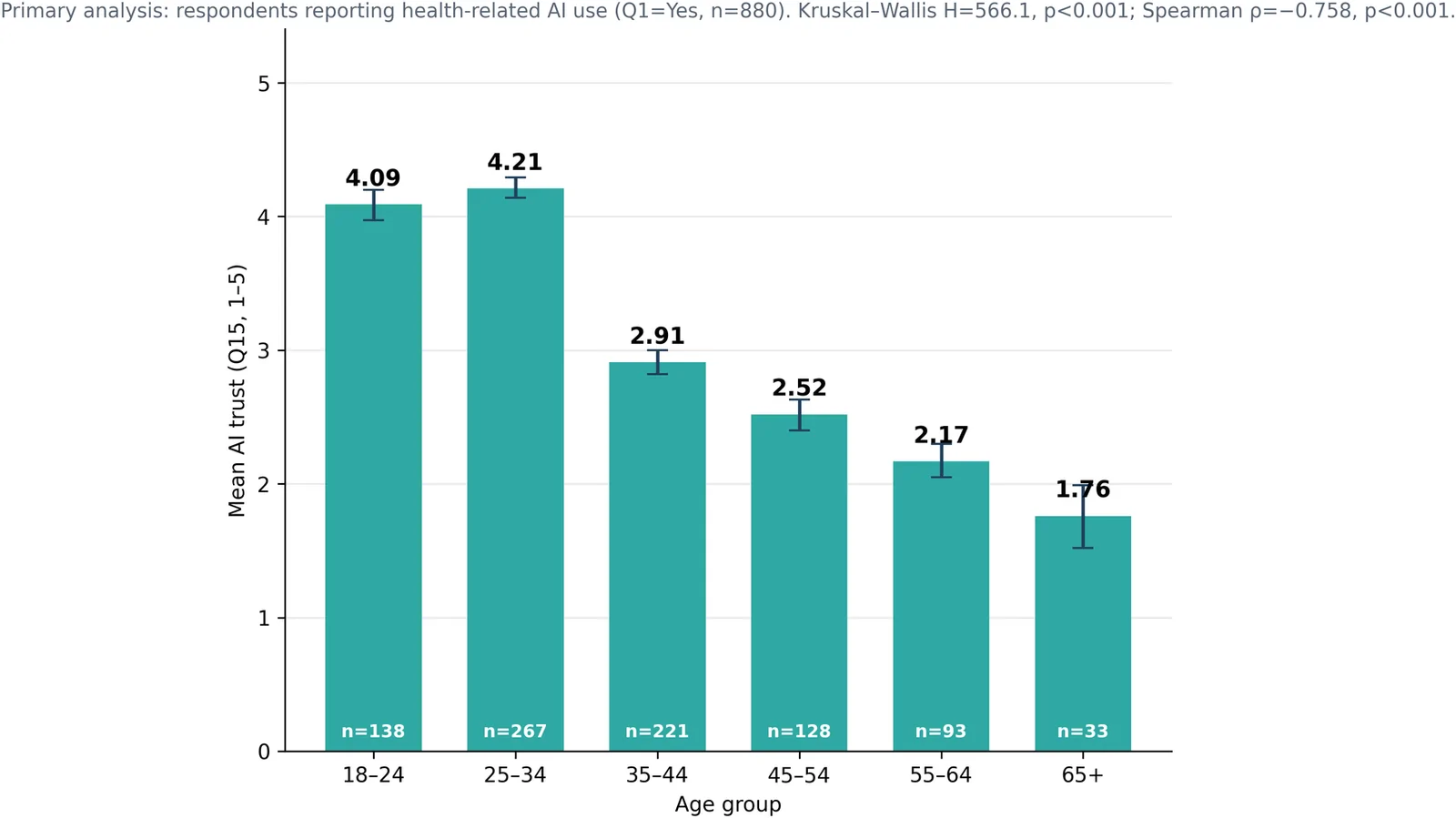
Ai trust by age group (mean, 95% CI). Primary analysis among respondents reporting health-related AI use (Q1 = Yes, *n* = 880); Kruskal–Wallis H = 566.1, *p* < 0.001; Spearman *ρ*=−0.758, *p* < 0.001.

AI trust (Q15, 1–5) differed substantially across age groups. The analysis among respondents reporting health-related AI use (Q1 = Yes, *n* = 880) is presented as the primary analysis: Kruskal–Wallis H = 566.1, *p* < 0.001; Spearman *r*s = −0.758, *p* < 0.001. The full-sample analysis (*N* = 1,044; H = 666.2, *r*s = −0.762) yielded a nearly identical result and is reported as a secondary analysis; because Q15 was displayed to all respondents, values from respondents without health-related AI experience may represent general or hypothetical attitudes rather than experience-based evaluations, and are reported separately in Supplementary File B. AI trust was high in both younger groups and highest among respondents aged 25–34 (mean 4.21/5), with the 18–24 group slightly below this peak (mean 4.09/5), and was substantially lower from age 35 onward, reaching its lowest value in the 65 + group (mean 1.76/5); the association is therefore strongly negative overall but not strictly monotonic across the two youngest groups (Table 3, Figure 4). Age-stratified trust distributions remained concentrated within one or two adjacent response categories, with interquartile ranges of 0.75–1.0 in the primary Q1 = Yes analysis (Table 3). AI trust was positively correlated with AI-use frequency and digital competency.

**Table 3:** AI trust (Q15, 1–5) by age group.

Age group	n (Q1 = Yes)	Mean (SD)	Median (IQR)	Mean 95% CI	Full-sample mean (n) [secondary]
18–24	138	4.09 (0.67)	4 (0.75)	3.97–4.20	4.09 (*n* = 141)
25–34	267	4.21 (0.64)	4 (1.0)	4.14–4.29	4.21 (*n* = 296)
35–44	221	2.91 (0.68)	3 (1.0)	2.82–3.00	2.94 (*n* = 265)
45–54	128	2.52 (0.65)	3 (1.0)	2.40–2.63	2.53 (*n* = 172)
55–64	93	2.17 (0.60)	2 (1.0)	2.05–2.30	2.22 (*n* = 122)
65+	33	1.76 (0.66)	2 (1.0)	1.52–1.99	1.79 (*n* = 48)

Primary analysis: respondents reporting health-related AI use (Q1 = Yes, *n* = 880). Kruskal–Wallis H = 566.1, *p* < 0.001; Spearman rs = −0.758, *p* < 0.001. Full-sample values (*N* = 1,044; H = 666.2, rs = −0.762) are given in the final column as a secondary analysis because Q15 was displayed to all respondents. IQR values of 0.75–1.0 reflect concentration of responses on one or two adjacent scale points within age groups.

Trust is high in both younger groups and highest at 25–34, then substantially lower from age 35 onward; the gradient is strongly negative overall but not strictly monotonic across the two youngest groups.

When asked whom they would trust more if a physician's diagnosis contradicted an earlier AI suggestion (Q17, all respondents), 52.9% (*n* = 552) selected ‘definitely the physician’ and 41.1% (*n* = 429) ‘rather the physician’; 6.0% (*n* = 63) were undecided, and no respondent selected either pro-AI option (0/1,044; 95% CI 0.0–0.4). Q17 presented a forced physician-versus-AI disagreement scenario and is interpreted as a boundary between general AI trust and explicit preference in a forced-choice conflict, not as a general measure of AI reliance. The Q15×Q17 association was graded (Spearman *ρ*=0.653, *p* < 0.001): higher Q15 trust corresponded to progressively less categorical physician preference and more undecided responses, although it did not cross into explicit preference for AI (Supplementary File C).

### 3.6 Gender-associated differences

**Figure 5:**
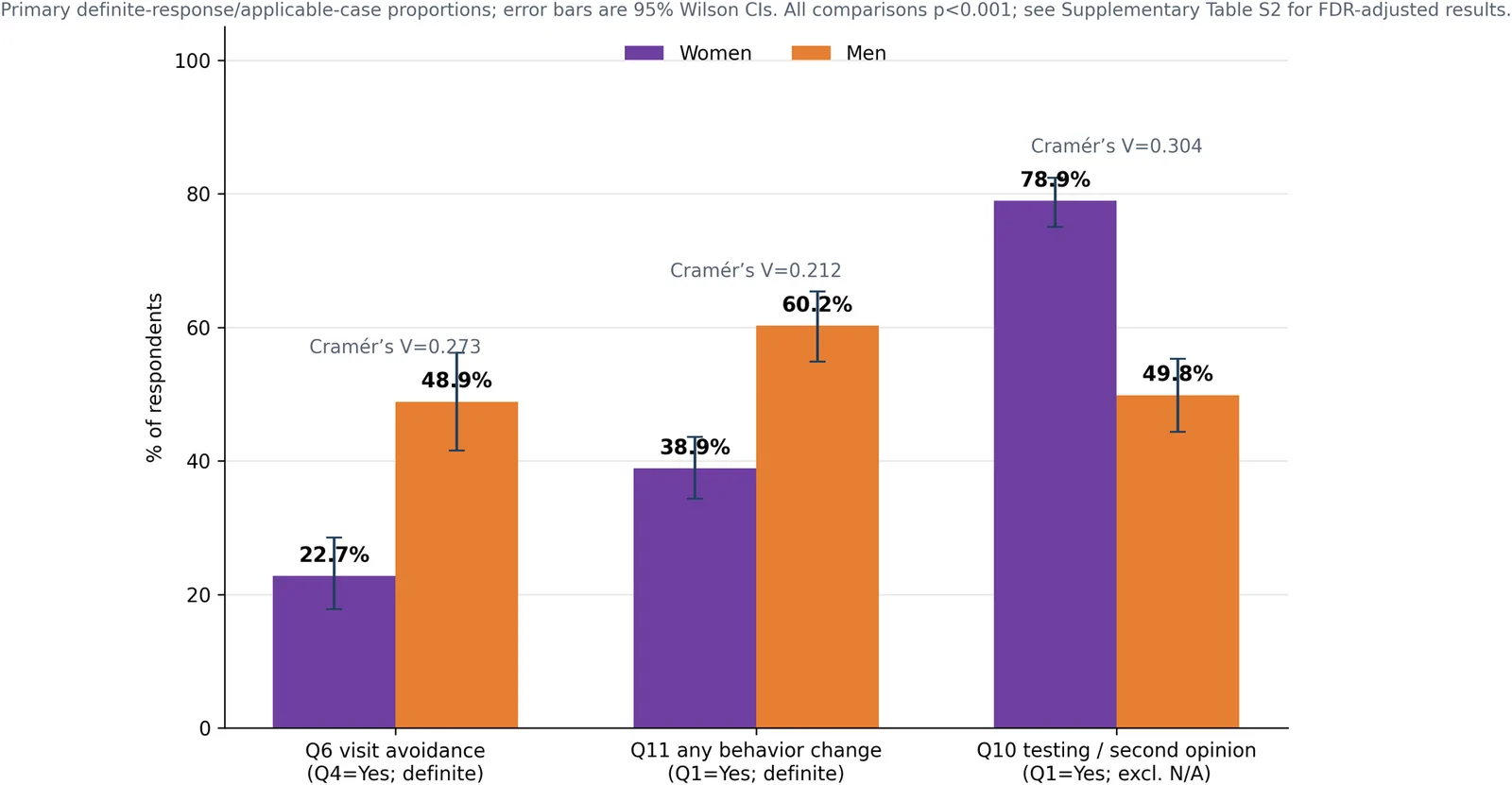
Gender-associated differences in key behavioral outcomes. Primary definite-response proportions (uncertainty/refusal/not-applicable excluded); error bars are 95% Wilson confidence intervals; all comparisons *p* < 0.001; FDR-adjusted results in Supplementary Table S2.

Gender-stratified analyses excluded respondents who selected ‘prefer not to say’ (*n* = 50 overall); binary behavioral comparisons were computed on definite Yes/No responses (uncertainty, refusal, and not-applicable excluded), consistent with the primary regression models. Among pre-consultation AI users, men reported a higher rate of visit avoidance (Q6: 48.9% [86/176] vs. women 22.7% [53/233]; *χ*^2^ = 30.48, *p* < 0.001, Cramér's V = 0.273). Women reported higher self-reported increase in health-related anxiety (Q9: mean 4.06 [*n* = 280] vs. men 3.41 [*n* = 212]; Mann–Whitney U = 43,256, *p* < 0.001, r_rb = 0.457). Among all AI health users, men reported a higher rate of any AI-associated health-behavior change (Q11: 60.2% [200/332] vs. women 38.9% [166/427]; *χ*^2^ = 34.15, *p* < 0.001, V = 0.212). Women reported a higher rate of additional testing and/or second-opinion requests (Q10: 78.9% [371/470] vs. men 49.8% [158/317]; *χ*^2^ = 72.72, *p* < 0.001, V = 0.304). Healthcare utilization frequency was higher in women (Q24, full-sample base, since this item was displayed to all respondents: mean 2.85 [*n* = 572] vs. men 1.96 [*n* = 422]; Mann–Whitney U = 168,697.5, r_rb = +0.398, *p* < 0.001; the comparison restricted to AI users, 2.81 [*n* = 485] vs. 1.99 [*n* = 353]; U = 117,062.5, r_rb = +0.368, is reported as a sensitivity analysis). AI-use frequency was lower in women (Q2: mean 2.24 vs. men 2.48; U = 69,701, r_rb = −0.186, *p* < 0.001). For the binary outcomes, the men-minus-women risk differences (with 95% Newcombe confidence intervals) were +26.1 percentage points (16.8–34.9) for Q6 visit avoidance, +21.4 points (14.2–28.2) for Q11 behavior change, and −29.1 points (−35.6 to −22.4) for Q10 additional testing and/or second-opinion requests; none of the intervals included zero. Full results, including the group-difference confidence intervals, are presented in Table 4 and Figure 5; all eight comparisons remained significant after Benjamini–Hochberg adjustment across the reported family (Supplementary Table S2). Uncertain and refusal responses were not evenly distributed by gender: for Q11 they were more frequent among women (12.0% vs. 5.9% in men; *χ*^2^ = 8.64, *p* = 0.003), and ‘not applicable’ responses to Q10 were more frequent among men (10.2% vs. 3.1%; *χ*^2^ = 18.05, *p* < 0.001) (this differential missingness is summarized in Supplementary File G). Response status was therefore associated with gender for Q11 and Q10; definite-response and applicable-case restriction cannot be assumed to be independent of this measured predictor. The direction of the gender differences is preserved under conservative coding (for Q10, retaining ‘not applicable’ as a non-event gives women 76.5% vs. men 44.8%, still *p* < 0.001), and outcome-coding sensitivity models retaining uncertain outcome responses (Supplementary Table S2, Panel C; these still exclude respondents who did not report gender) reach the same conclusions, but the gender estimates should be read with this differential response status in mind. Under inverse-probability weighting for the probability of a definite response, the Q11 male-gender odds ratio was essentially unchanged (2.40 to 2.43), and across boundary reassignments of the uncertain Q11 responses and of the Q10 ‘not applicable’ responses the direction and significance of the gender difference were preserved; this tipping analysis enumerated 1,298 threshold scenarios for Q11 and 592 for Q10, representing systematic group-level boundary reassignments of the uncertain and not-applicable responses rather than all 2^n individual-level assignment combinations (Supplementary File M).

**Table 4:** Gender-associated differences in behavioral outcomes.

Outcome (base)	Women	Men	Test; p	Effect size	Difference (men−women); 95% CI for risk differences	FDR q
Q6 visit avoidance (Q4 = Yes, definite)	22.7% (53/233)	48.9% (86/176)	*χ*^2^ = 30.48; *p* < 0.001	V = 0.273	+26.1 pp (+16.8, +34.9)	5.4 × 10⁻⁸
Q9 increase in health anxiety (Q4 = Yes)	mean 4.06 (*n* = 280)	mean 3.41 (*n* = 212)	U = 43,256; *p* < 0.001	r_rb=+0.457	−0.65 pts (mean diff)	2.1 × 10⁻^20^
Q10 additional testing and/or second opinion (Q1 = Yes, excl. N/A)	78.9% (371/470)	49.8% (158/317)	χ^2^ = 72.72; *p* < 0.001	V = 0.304	−29.1 pp (−35.6, −22.4)	4.0 × 10⁻^17^
Q10 additional testing, any (Q1 = Yes, excl. N/A)	58.9% (277/470)	40.1% (127/317)	χ^2^ = 26.99; *p* < 0.001	V = 0.185	−18.9 pp (−25.7, −11.8)	2.7 × 10⁻⁷
Q10 second-opinion request, any (Q1 = Yes, excl. N/A)	43.0% (202/470)	26.2% (83/317)	χ^2^ = 23.12; *p* < 0.001	V = 0.171	−16.8 pp (−23.2, −10.1)	1.5 × 10⁻⁶
Q11 any AI-associated behavior change (Q1 = Yes, definite)	38.9% (166/427)	60.2% (200/332)	χ^2^ = 34.15; *p* < 0.001	V = 0.212	+21.4 pp (+14.2, +28.2)	1.0 × 10⁻⁸
Q24 healthcare utilization frequency (full sample)	mean 2.85 (*n* = 572)	mean 1.96 (*n* = 422)	U = 168,697.5; *p* < 0.001	r_rb=+0.398	−0.89 pts (mean diff)	1.6 × 10⁻^27^
Q2 AI-use frequency (Q1 = Yes)	mean 2.24 (*n* = 485)	mean 2.48 (*n* = 353)	U = 69,701; *p* < 0.001	r_rb = −0.186	+0.24 pts (mean diff)	3.6 × 10⁻⁷

Respondents selecting ‘prefer not to say’ for gender excluded. Binary outcomes use definite Yes/No responses; Q10 excludes ‘not applicable’. Q24 uses the full sample because that item was displayed to all respondents. Mann–Whitney effect signs follow a women-vs-men convention (positive = higher in women). FDR = Benjamini–Hochberg q across the eight reported gender comparisons (Q2, Q6, Q9, Q10 composite, Q10 testing, Q10 second-opinion, Q11, Q24) as one family; all eight remain significant after adjustment. The difference column gives the men-minus-women risk difference in percentage points with a 95% Newcombe confidence interval for binary outcomes; for the ordinal items (Q9, Q24, Q2) a 95% CI is not defined for a percentage-point difference, so the difference of group means is shown for orientation only, accompanied by the rank-biserial correlation as a standardized effect size (a measure of effect magnitude, not of precision); p-values from two-sided asymptotic Mann–Whitney U tests with tie correction are reported alongside.

Binary risk differences are men minus women; r_rb is positive when women score higher (Q2 negative = women lower). The Q24 comparison restricted to AI users (women 2.81, *n* = 485; men 1.99, *n* = 353; U = 117,062.5; r_rb=+0.368) is reported as a sensitivity analysis in Supplementary Table S2.

### 3.7 Logistic regression

**Figure 6:**
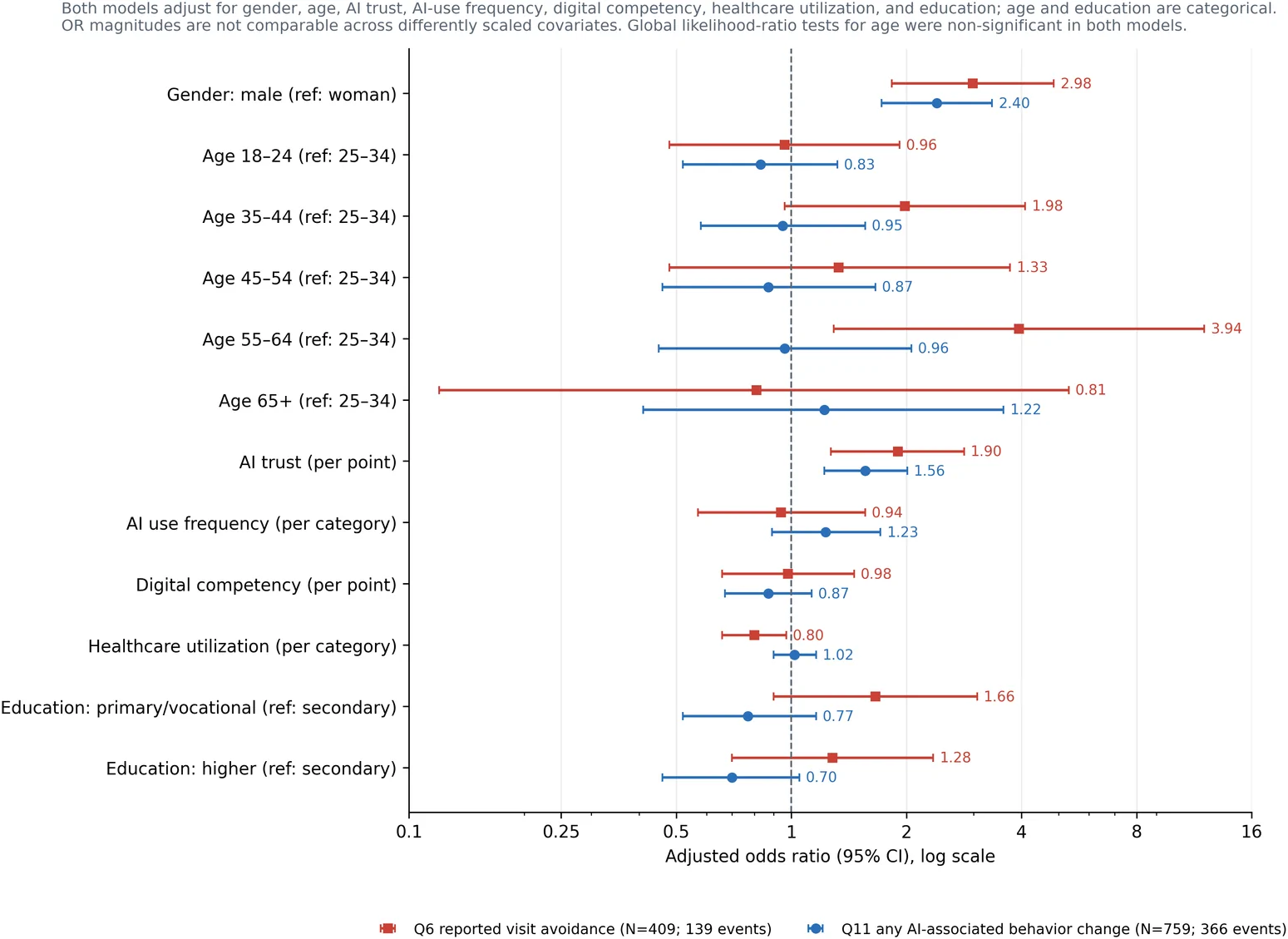
Adjusted odds ratios (95% CI) from the primary definite-response logistic models for reported visit avoidance (Q6) and any AI-associated health-behavior change (Q11).

Primary binary logistic models were restricted to respondents providing definite Yes/No outcome responses (Table 5, Figure 6). For Q6 (reported visit avoidance; *N* = 409 with 139 events after excluding ‘prefer not to say’ for gender), male gender was associated with the outcome (OR = 2.98, 95% CI 1.83–4.86, *p* < 0.001) as was greater AI trust (OR = 1.90, 95% CI 1.27–2.83, *p* = 0.002); model discrimination was modest (AUC=0.720; Nagelkerke R^2^ = 0.183; Hosmer–Lemeshow *p* = 0.90). Among the categorical age contrasts, the 55–64 group showed higher odds relative to the 25–34 reference (OR = 3.94, 95% CI 1.29–12.02; raw *p* = 0.016), an estimate based on few events; this single contrast did not remain significant after Benjamini–Hochberg adjustment of the model coefficients (q = 0.064), and the global likelihood-ratio test for age as a whole was not significant (*χ*^2^ = 10.80, df = 5, *p* = 0.056), so no independent age effect is claimed. The global test for education was also non-significant (*χ*^2^ = 2.93, df = 2, *p* = 0.231). Lower healthcare-utilization frequency was associated with visit avoidance in the per-category term (OR = 0.80, 95% CI 0.66–0.97) but did not remain significant after Benjamini–Hochberg adjustment.

**Table 5:** Binary logistic regression — primary definite-response models.

Variable	Q6 OR	Q6 95% CI	Q6 p	Q11 OR	Q11 95% CI	Q11 p
Gender: male (ref: woman)	2.98	1.83–4.86	<0.001	2.40	1.72–3.35	<0.001
Age 18–24 (ref: 25–34)	0.96	0.48–1.92	0.914	0.83	0.52–1.32	0.428
Age 35–44 (ref: 25–34)	1.98	0.96–4.09	0.065	0.95	0.58–1.56	0.834
Age 45–54 (ref: 25–34)	1.33	0.48–3.73	0.584	0.87	0.46–1.66	0.677
Age 55–64 (ref: 25–34)	3.94	1.29–12.02	0.016	0.96	0.45–2.06	0.911
Age 65+ (ref: 25–34)	0.81	0.12–5.32	0.823	1.22	0.41–3.59	0.720
AI trust (Q15, per point)	1.90	1.27–2.83	0.002	1.56	1.22–2.01	<0.001
AI use frequency (Q2, per category)	0.94	0.57–1.56	0.811	1.23	0.89–1.71	0.216
Digital competency (Q20, per point)	0.98	0.66–1.46	0.929	0.87	0.67–1.13	0.306
Healthcare utilization (Q24, per category)	0.80	0.66–0.97	0.025	1.02	0.90–1.16	0.732
Education: primary/vocational (ref: secondary)	1.66	0.90–3.06	0.105	0.77	0.52–1.16	0.212
Education: higher (ref: secondary)	1.28	0.70–2.35	0.416	0.70	0.46–1.05	0.087
N (events)	409 (139)			759 (366)		
Nagelkerke R^2^	0.183			0.139		
AUC (ROC)	0.720			0.687		
Hosmer–Lemeshow p	0.90			0.72		

Primary models restricted to respondents providing definite Yes/No outcome responses. Reference category: gender = woman; age reference = 25–34; education reference = secondary. Odds ratios with 95% confidence intervals. Global likelihood-ratio tests (whole covariate vs. its omission): Q6 age χ^2^ = 10.80, df = 5, *p* = 0.056; Q6 education χ^2^ = 2.93, df = 2, *p* = 0.231; Q11 age χ^2^ = 1.12, df = 5, *p* = 0.952; Q11 education χ^2^ = 3.90, df = 2, *p* = 0.143. The originally submitted models coding ‘don't remember’/‘prefer not to answer’ as no reported occurrence are retained as sensitivity analyses (Supplementary Table S2).

Global likelihood-ratio tests for the multi-level covariates were non-significant in both models, so individual category contrasts (including Age 55–64 in the Q6 model, which does not survive Benjamini–Hochberg adjustment of the model coefficients, q = 0.064) are not interpreted as independent effects. Maximum VIF 3.26 (Q6) and 3.01 (Q11), both for AI trust. AUC values are apparent (in-sample); internal bootstrap validation gives optimism-corrected AUC of about 0.68 (Q6) and 0.66 (Q11), and the models are exploratory, not externally validated (Supplementary File L).

For Q11 (any AI-associated health-behavior change; *N* = 759 with 366 events), male gender (OR = 2.40, 95% CI 1.72–3.35, *p* < 0.001) and greater AI trust (OR = 1.56, 95% CI 1.22–2.01, *p* < 0.001) were associated with the outcome; model discrimination and explained variation were modest (AUC=0.687; Nagelkerke R^2^ = 0.139; Hosmer–Lemeshow *p* = 0.72). Age group, AI-use frequency, digital competency, education, and healthcare utilization were not independently associated with the outcome; global likelihood-ratio tests were non-significant for both multi-level covariates (age *χ*^2^ = 1.12, df = 5, *p* = 0.95; education *χ*^2^ = 3.90, df = 2, *p* = 0.14). The originally submitted models coding ‘don't remember’ and ‘prefer not to answer’ as no reported occurrence are retained as sensitivity analyses and yielded the same substantive conclusions. Because covariates were measured on different scales, OR magnitudes are not directly comparable as measures of the 'strength' of association. Bootstrap internal validation (1,000 valid resamples; the Q6 model required 1,122 attempts to obtain 1,000 valid fits after 122 non-convergent or numerically failed refits, so its corrected estimates are conditional on successful refitting) indicated performance optimism in both models. For Q6, AUC decreased from 0.720 apparent to 0.684 optimism-corrected, Nagelkerke R^2^ from 0.183 to 0.096, with a corrected calibration slope of 0.793 and intercept −0.114; the Q6 model has limited events per parameter (139 events, 12 coefficients plus an intercept), sparse older-age cells (65+: *n* = 15 with 2 events), and failed to refit in a small fraction of resamples, so its individual coefficients—particularly the age contrasts—are unstable exploratory estimates. For Q11, AUC decreased from 0.687 to 0.665, Nagelkerke R^2^ from 0.139 to 0.099, with calibration slope 0.871 and intercept −0.009. The Hosmer–Lemeshow p-values are not presented as evidence of adequate calibration. The models showed modest apparent discrimination that decreased after bootstrap optimism correction; neither model was externally validated, and the analyses are exploratory throughout (Supplementary File L). The direction of the male-gender and AI-trust associations was robust across standard, Firth penalized (Q6 male OR 2.87, Q11 2.36), and ridge-penalized models, after removal of influential observations, and across 1,000 bootstrap resamples; the Q6 effect magnitudes were more model-dependent than Q11 (for example, ridge shrinkage lowered the Q6 male OR toward 1.8), consistent with the Q6 model's limited stability, and are reported as exploratory (Supplementary File N).

### 3.8 Attitudes toward disclosure and patient education

Among all respondents, 71.8% (*n* = 750) believed that physicians should ask patients whether they used AI before a visit (Q18; 12.0% no, 16.2% undecided), and 74.6% (*n* = 779) believed that healthcare facilities should educate patients on how to safely use AI for health-related matters (Q19; 8.4% no, 17.0% undecided).

## 4 Discussion

### 4.1 Disclosure: what the survey measured

Among respondents reporting AI use before a medical visit, a large majority (82.5%, 428/519) selected ‘No’ when asked whether they had informed a physician about that AI use, and this proportion was similarly high across reported visit-avoidance strata (79.2–86.8%). Does this mean that roughly four in five patients conceal AI use from their doctors? Not from these data. Because the survey did not confirm consultation attendance, establish a single index episode, or ensure that Q6 and Q13 referred to the same experience, this proportion should be read as the share selecting ‘No’ on the disclosure item among pre-consultation AI users, not as the rate of non-disclosure within completed clinical encounters. The actual disclosure rate among patients who attended a consultation cannot be estimated from this survey.

Read through the Network Episode Model as an interpretive lens (12), generative AI can be viewed as an additional informal source that patients may consult before engaging formal care; the model suggests, but the present survey does not establish, that such lay consultation is often not disclosed to clinicians. A similar pattern of 'seeking but not discussing' has been documented for online health information more broadly (23), and the directness with which patients share such information with physicians is shaped by health literacy, perceived communication competence, and the quality of the physician–patient relationship (24, 25). In the present sample, fear of a negative physician reaction was the most frequently selected Q14 option. The mechanisms underlying this selection were not directly measured; item wording, fixed response-option order, multiple-response acquiescence, social desirability, and the recruitment channel cannot be excluded as contributors and are discussed below.

### 4.2 Anxiety, diagnostic demand, and possible interpretive mechanisms

The reported questioning of physician recommendations (Q16) and requests for additional testing and/or a second opinion (Q10) are compatible with several interpretations, including patient activation, uncertainty reduction, health-related anxiety, and reliance on AI-generated information (14, 15). Because automation bias and cyberchondria were not directly measured, the present results cannot distinguish between these mechanisms or establish that either occurred. The higher self-reported anxiety and additional-testing and/or second-opinion requests among women resemble patterns described in the cyberchondria literature (19–21); cyberchondria itself was not directly measured, and this parallel is offered as an interpretive frame only. The substantial inverse association between AI trust and self-reported anxiety increase (rs = −0.538) complicates a simple interpretation in which generative AI uniformly amplifies health anxiety. One possible explanation is that respondents who regarded AI-generated information as more credible experienced it as reassuring, whereas respondents who reported greater anxiety subsequently evaluated that information as less trustworthy. The reverse direction, common-cause explanations, and differences in the content of individual AI responses are equally possible. Because trust and perceived anxiety change were measured contemporaneously using single items, the temporal direction and underlying mechanism cannot be determined. Self-reported medication cessation without physician consultation may represent a potential safety signal but should not be interpreted as evidence of clinically dangerous discontinuation in all cases; medication class and indication were not captured, so the clinical risk cannot be graded (26, 27).

### 4.3 AI trust and relational features of conversational systems

One might expect trust in an unfamiliar technology to grow with cautious exposure; here it did the opposite, standing highest among the youngest respondents and falling steeply from age 35 onward. Given the documented limitations and variable accuracy of LLM-generated medical responses (6, 28, 29), the high trust reported by younger respondents is worth examining critically rather than reading as reassurance; anthropomorphism research (16, 17) offers one interpretive lens, as empathetic, first-person language may build trust through relational rather than informational channels. The observed age pattern may reflect cohort differences in AI exposure and the social-media recruitment frame, but these mechanisms were not measured and this explanation remains speculative.

### 4.4 Gender-associated response patterns in this selected sample

In primary definite-response models, male gender and greater AI trust were consistently associated with the two modeled outcomes (reported visit avoidance and any AI-associated health-behavior change), holding covariates constant, whereas AUC values and explanatory power were modest, indicating that most variability was not captured by the measured variables. Unmeasured factors likely to influence these behaviors include symptom severity, previous healthcare experiences, trust in physicians, baseline health anxiety, chronic-disease status, socioeconomic position, and the specific content of AI responses. Because gender was self-reported and the study was not designed to assess biological-sex mechanisms, gender-associated findings should be interpreted as exploratory behavioral associations rather than evidence of biological sex differences. These sample-specific associations do not establish clinically distinct patient subgroups and do not by themselves justify differentiated clinical interventions.

### 4.5 Clinical communication implications

These findings should not be interpreted as supporting a validated clinical protocol. The high proportion selecting ‘No’ on Q13, particularly when accompanied by fear of a negative physician reaction, suggests a possible communication barrier; we advance this as a hypothesis rather than a demonstrated gap, because attendance, opportunity to disclose, and a shared index episode were not established. It would be worth testing whether simple, normalizing questions about pre-consultation AI use, asked routinely, can improve disclosure and reduce misunderstanding. Such approaches appear acceptable to respondents themselves (71.8% supported routine physician inquiry; 74.6% supported patient education). Two separate items are relevant here and are not directly comparable: Q17, a hypothetical forced-choice item displayed to all 1,044 respondents, recorded no explicit pro-AI selection, whereas Q13, analyzed among respondents reporting AI use before a medical visit (*n* = 519), was answered ‘No’ by most of them. The items differ in framing, denominator, and reference situation. The coexistence of a zero pro-AI response in the forced physician-versus-AI scenario and frequent selection of ‘No’ on Q13 should not be interpreted as distinguishing relational from attitudinal mechanisms. The present data show only that anticipated negative physician reactions and concern about challenging clinical authority were the most frequently selected Q14 reasons. Whether these responses reflect previous relational experiences, anticipated stigma, general attitudes toward physicians, social-desirability effects, or other mechanisms requires direct investigation.

### 4.6 Strengths and limitations

How much of this pattern is signal, and how much is an artifact of how the survey was built? Both possibilities are considered below. Strengths include a relatively large sample of digitally active adults, explicit reporting of denominators under the skip logic, a documented consent and routing procedure, item-faithful terminology, definite-response primary models with a retained sensitivity analysis, an FDR sensitivity analysis, and a public reproducibility package with predefined computational checks recomputing the reported values.

The study has important limitations. Recruitment used a convenience-based, Instagram-based multi-account dissemination strategy and did not create a probability-based sampling frame; dissemination through 15 directly approached independent accounts and subsequent sharing by 101 unique accounts broadened exposure, but the number of unique individuals exposed, overlap between account audiences, Instagram platform algorithms, self-selection, and response yield from each source could not be determined. Professional background was not systematically coded for all 101 accounts involved in subsequent organic redistribution; therefore, circulation within professional medical networks beyond the 15 directly contacted accounts could not be determined. The participant-facing survey title (‘The patient after a chatbot consultation…’) differed from the manuscript title and may itself have framed the items toward a more clinical and episodic interpretation than the items literally specify. Selection may also have influenced the magnitude and potentially the direction of associations observed within the sample, including the age–trust, gender–behavior, and trust–behavior relationships; the reported odds ratios, effect sizes, and correlations should therefore be interpreted as sample-specific exploratory estimates and should not be assumed to generalize to the broader Polish population. No paid advertising or promotion was used. The first author's account focused on AI and professional education, which may have preferentially attracted respondents interested in generative AI. Although the recruitment materials and visible biography did not display a LUX MED affiliation, limited exposure among individuals who knew the first author personally or professionally cannot be completely excluded. The observed prevalence of health-related AI use and the reported attitudes should therefore not be interpreted as population estimates for Polish adults, and comparison with Polish benchmarks confirmed substantial selection by age and education (Supplementary Files E and K).

The survey did not identify a single index clinical episode or require Q4, Q6, Q10, Q11, Q13, and Q16 to refer to the same event, and several items lacked a fixed recall period; responses may therefore reflect different episodes occurring at different times, and associations across items should not be interpreted as a verified sequential pathway from AI use to consultation, disclosure, or subsequent behavior. The *de novo* instrument was not psychometrically validated, and several constructs were represented by single self-report items; automation bias, anthropomorphism, and cyberchondria were not measured with validated scales and remain interpretive frameworks rather than tested constructs. Q14 used a fixed option order, and Q17 used a forced physician-versus-AI disagreement scenario; wording, order, normative framing, and social-desirability effects may have contributed to the observed response patterns, including the frequent selection of fear of a negative physician reaction and the zero pro-AI count. Completion time, IP address, device, and dedicated bot-detection data were not available. Uncertain-response rates differed by gender for some items (Section 3.6), so response status was associated with gender for selected items and definite-response/applicable-case restriction cannot be assumed independent of it; influential-observation diagnostics and corresponding regression-stability sensitivity analyses were performed (Supplementary File N), but no external validation cohort was available; and because a single responder link in a survey anonymous with respect to the investigators was shared across many accounts, no source identifier was available, so possible clustering of responses within recruiting accounts could not be assessed or adjusted for. Several data-quality patterns typical of an anonymous convenience sample—a zero pro-AI count on Q17, a strong but non-deterministic age–trust gradient (Cramér's V = 0.460, with substantial within-age response variation), and one exact-duplicate short-route record pair—were examined in a structural response-quality audit (Supplementary File L), retained, and reported transparently rather than excluded. Self-report is subject to social-desirability bias, and the cross-sectional design precludes causal inference. No independent institutional determination confirming that ethics review was unnecessary was obtained; although the study was assessed by the authors as falling outside the Polish statutory definition of a medical experiment, the absence of an independent determination remains an administrative limitation.

## 5 Conclusions

In this social-media-recruited convenience sample of digitally engaged Polish adults, generative AI was frequently reported as a resource used before a medical visit, and most such respondents selected ‘No’ when asked whether they had informed a physician. Because consultation attendance and a defined index episode were not confirmed, the actual disclosure rate within completed clinical encounters remains unknown. In this sample, the reasons most frequently selected were fear of a negative physician reaction and reluctance to undermine clinical authority; the mechanisms underlying these responses were not directly measured, and fixed response-option order and social-desirability effects were not excluded. In primary definite-response models, male gender and greater AI trust remained associated with both modeled outcomes (reported visit avoidance and any AI-associated health-behavior change). These preliminary and exploratory results, drawn from a convenience sample and a *de novo* instrument, support future prospective, episode-based research using validated instruments, probability-based recruitment, longitudinal follow-up and preregistration, and direct measurement of how AI-derived information enters clinical communication and care-seeking decisions. Whether the frequent selection of ‘No’ on Q13 reflects a clinically meaningful communication phenomenon or an artifact of a convenience-sample instrument cannot be resolved by these data; its consistency across descriptive strata makes it a hypothesis worth testing in prospectively anchored clinical episodes.

## Data Availability

The datasets presented in this study can be found in online repositories. The names of the repository/repositories and accession number(s) can be found below: Open Science Framework (OSF), https://doi.org/10.17605/OSF.IO/7Z6CW. The repository contains the analysis code, a separately written computational-verification script with expected values, aggregate response distributions, figure-source data, the questionnaire with the participant-information and consent screens, the routing specification and form-configuration screenshots, the software environment, and SHA-256 checksums. The individual-level dataset is not openly deposited, because the participant information described data sharing as conditional on assessment of a request, compatibility with the scope of consent, and appropriate safeguards rather than unrestricted public release; de-identified individual-level data may be considered upon reasonable request to the corresponding author.

## Supplementary material

Figure S1 Programmed questionnaire routing (as implemented). N/D denotes an item not displayed by the routing and is not respondent-level missingness.

Supplementary Data Sheet 2 Screenshots of the fielded questionnaire configuration, documenting response settings and the programmed branching rules.

Supplementary Data Sheet 3 Supplementary tables, diagnostic output, sensitivity analyses and data-fidelity documentation.
