# Supplementary material for: Generative AI use before medical visits: disclosure-item responses, trust, and care-seeking behaviors in a cross-sectional social-media survey in Poland
Source: Front Digit Health. 2026 Aug 27;8:1933451. doi: 10.3389/fdgth.2026.1933451 (PMC13558417; doi:10.3389/fdgth.2026.1933451)

Supplementary Material S1. Survey instrument (bilingual: Polish | English)

Manuscript: Generative AI use before medical visits: disclosure-item responses, trust, and care-seeking behaviors in a cross-sectional social-media survey in Poland

The questionnaire was administered to participants exclusively in Polish; the English text was never displayed to respondents. The English column is a literal parallel translation prepared and cross-checked by the authors after data collection, provided solely for transparency and to allow reviewers and international readers to understand the instrument. The manuscript uses the literal wording of Q4 throughout—AI use before a medical visit ("wizyta lekarska"); the item does not specify timing or confirm that a visit took place, and these limits on interpretation are stated in the manuscript's limitations rather than built into the item label. The translation was prepared without generative AI; no formal back-translation or cross-cultural validation of the English version was performed. Item numbers correspond to the question identifiers (Q1–Q24) used throughout the manuscript.

**Polski (oryginał)**	**English (translation)**
**ANKIETA BADAWCZA**	**RESEARCH SURVEY**
Pacjent po konsultacji z chatbotem: wpływ AI na zaufanie do diagnozy lekarskiej, oczekiwania diagnostyczne i decyzje o korzystaniu z opieki medycznej	*The patient after a chatbot consultation: the influence of AI on trust in medical diagnosis, diagnostic expectations, and decisions regarding healthcare use*
Badanie prowadzone jest anonimowo. Wyniki zostaną wykorzystane wyłącznie w celach naukowych. Wypełnienie ankiety zajmuje około 5–7 minut.	*This survey is anonymous. The results will be used exclusively for scientific purposes. Completion takes approximately 5–7 minutes.*
**A. Korzystanie z AI w sprawach zdrowotnych**	**A. AI use for health-related purposes**
**1. Czy kiedykolwiek korzystał/a Pan/Pani z narzędzi AI (np. ChatGPT, Gemini, Claude, Grok, DeepSeek, Copilot) w sprawach zdrowotnych? ○ Tak ○ Nie ○ Nie wiem / nie pamiętam [Jeśli NIE lub NIE WIEM / NIE PAMIĘTAM – proszę przejść do pytania 15.]**	**1. Have you ever used AI tools (e.g., ChatGPT, Gemini, Claude, Grok, DeepSeek, Copilot) for health-related purposes? ○ Yes ○ No ○ I don't know / don't remember [If NO or I DON'T KNOW / DON'T REMEMBER – please proceed to question 15.]**
**2. Jak często korzysta lub korzystał/a Pan/Pani z AI w sprawach zdrowotnych?** ○ Regularnie (kilka razy w miesiącu lub częściej) ○ Kilka razy ○ Raz lub dwa razy ○ Tylko raz, próbowałem/am	**2. How often do you (or did you) use AI for health-related purposes?** ○ Regularly (several times a month or more) ○ Several times ○ Once or twice ○ Only once, just to try
**3. W jakim celu korzystał/a Pan/Pani z AI w sprawach zdrowotnych? [Można zaznaczyć kilka odpowiedzi.]** ☐ Sprawdzenie możliwych przyczyn objawów ☐ Interpretacja wyników badań ☐	**3. For what purposes have you used AI in health-related matters? [Multiple answers allowed.]** ☐ Checking possible causes of symptoms ☐ Interpreting test results ☐ Understanding a physician's

Zrozumienie diagnozy lekarza ☐ Zrozumienie zaleceń lub przyjmowanych leków ☐ Przygotowanie pytań przed wizytą ☐ Ocena, czy sytuacja wymaga wizyty lekarskiej ☐ Wybór odpowiedniego specjalisty ☐ Uspokojenie się / redukcja lęku ☐ Weryfikacja opinii lekarza ☐ Inne: ________	diagnosis ☐ Understanding recommendations or medications ☐ Preparing questions before a visit ☐ Assessing whether the situation requires a medical visit ☐ Choosing an appropriate specialist ☐ Reassurance / anxiety reduction ☐ Verifying a physician's opinion ☐ Other: ________
**B. Wpływ AI na decyzje o kontakcie z lekarzem**	**B. AI influence on decisions to contact a physician**
**4. Czy korzystał/a Pan/Pani z AI przed wizytą lekarską? ○ Tak ○ Nie ○ Nie pamiętam [Jeśli NIE lub NIE PAMIĘTAM – proszę przejść do pytania 10.]**	**4. Have you used AI before a medical visit? ○ Yes ○ No ○ I don't remember [If NO or I DON'T REMEMBER – please proceed to question 10.]**
**5. Czy informacje z AI wpłynęły na decyzję o kontakcie z lekarzem?** ○ Tak, przyspieszyły decyzję o wizycie ○ Tak, opóźniły decyzję o wizycie ○ Tak, zrezygnowałem/am z wizyty ○ Nie wpłynęły	**5. Did information from AI influence your decision to contact a physician?** ○ Yes, it accelerated my decision to attend ○ Yes, it delayed my decision to attend ○ Yes, I decided not to attend ○ No, it had no influence
**6. Czy zdarzyło się Panu/Pani zrezygnować z wizyty, ponieważ odpowiedź AI zasugerowała, że problem nie wymaga konsultacji?** ○ Tak ○ Nie ○ Nie pamiętam ○ Wolę nie odpowiadać	**6. Have you ever given up a medical visit because the AI response suggested that the problem did not require a consultation? ○ Yes ○ No ○ I don't remember ○ Prefer not to answer**
**7. Czy zdarzyło się Panu/Pani umówić wizytę szybciej, ponieważ AI zasugerowało możliwe poważne przyczyny objawów?** ○ Tak ○ Nie ○ Nie pamiętam	**7. Have you ever scheduled a medical visit sooner because the AI suggested possible serious causes of your symptoms?** ○ Yes ○ No ○ I don't remember
**8. Czy po skorzystaniu z AI przed wizytą czuł/a się Pan/Pani lepiej przygotowany/a do rozmowy z lekarzem?** 1 – zdecydowanie nie 2 3 4 5 – zdecydowanie tak	**8. After using AI before a visit, did you feel better prepared for the consultation with your physician?** 1 – definitely no 2 3 4 5 – definitely yes
**9. Czy informacje uzyskane z AI zwiększyły Pana/Pani niepokój o zdrowie?** 1 – zdecydowanie nie 2 3 4 5 – zdecydowanie tak	**9. Did the information obtained from AI increase your health-related anxiety?** 1 – definitely no 2 3 4 5 – definitely yes
**C. Wpływ AI na relację z lekarzem i decyzje terapeutyczne**	**C. AI influence on the physician relationship and therapeutic decisions**
**10. Czy zdarzyło się Panu/Pani poprosić lekarza o dodatkowe badania lub drugą opinię po skorzystaniu z AI?** ○ Tak,	**10. Have you asked a physician for additional tests or a second opinion after using AI?** ○ Yes, I asked for

poprosiłem/am o dodatkowe badania ○ Tak, poprosiłem/am o drugą opinię ○ Tak, jedno i drugie ○ Nie ○ Nie dotyczy	additional tests ○ Yes, I asked for a second opinion ○ Yes, both ○ No ○ Not applicable
**11. Czy zdarzyło się Panu/Pani zmienić swoje postępowanie zdrowotne bez konsultacji z lekarzem po skorzystaniu z AI?** ○ Tak ○ Nie ○ Nie pamiętam ○ Wolę nie odpowiadać	**11. Have you ever changed your health-related behavior without consulting a physician after using AI?** ○ Yes ○ No ○ I don't remember ○ Prefer not to answer
**12. Jeśli tak – czego dotyczyła ta zmiana? [Można zaznaczyć kilka odpowiedzi.]** ☐ Przyjęcia leku bez recepty ☐ Zmiany dawkowania leku ☐ Odstawienia leku ☐ Samodzielnego wykonania badań ☐ Zmiany diety lub suplementacji ☐ Rezygnacji z wizyty ☐ Pilnego zgłoszenia się do lekarza ☐ Inne: _______	**12. If yes – what did the change concern? [Multiple answers allowed.]** ☐ Taking an over-the-counter medication ☐ Changing medication dosage ☐ Discontinuing a medication ☐ Self-arranged tests ☐ Diet or supplementation change ☐ Cancelling a visit ☐ Seeking urgent medical attention ☐ Other: _______
**13. Czy powiedział/a Pan/Pani lekarzowi, że korzystał/a z AI w tej sprawie?** ○ Tak ○ Nie ○ Nie pamiętam ○ Nie dotyczy	**13. Did you tell your physician that you had used AI in this matter?** ○ Yes ○ No ○ I don't remember ○ Not applicable
**14. Jeśli nie – dlaczego? [Można zaznaczyć kilka odpowiedzi.]** ☐ Nie uważałem/am tego za ważne ☐ Bałem/am się negatywnej reakcji lekarza ☐ Nie było czasu ☐ Lekarz nie zapytał ☐ Nie chciałem/am podważać autorytetu lekarza ☐ Inne: _______	**14. If no – why? [Multiple answers allowed.]** ☐ I did not consider it important ☐ I was afraid of a negative physician reaction ☐ There was no time ☐ The physician did not ask ☐ I did not want to undermine the physician's authority ☐ Other: _______
**D. Zaufanie do AI i do lekarza**	**D. Trust in AI and in the physician**
**15. Na ile ufa Pan/Pani informacjom zdrowotnym uzyskanym z AI?** 1 – wcale nie ufam 2 3 4 5 – zdecydowanie ufam	**15. How much do you trust health information obtained from AI?** 1 – I do not trust at all 2 3 4 5 – I fully trust
**16. Czy zdarzyło się Panu/Pani podważyć diagnozę lub zalecenia lekarza po tym, jak AI zasugerowało coś innego?** ○ Tak ○ Nie ○ Nie pamiętam ○ Nie dotyczy	**16. Have you ever questioned a physician's diagnosis or recommendations after AI suggested something different?** ○ Yes ○ No ○ I don't remember ○ Not applicable
**17. Jeśli diagnoza lekarza jest sprzeczna z tym, co wcześniej zasugerowała AI – komu Pan/Pani bardziej ufa?** ○ Zdecydowanie lekarzowi ○ Raczej lekarzowi ○ Trudno powiedzieć ○ Raczej AI ○ Zdecydowanie AI	**17. If a physician's diagnosis contradicts what AI previously suggested – whom do you trust more?** ○ Definitely the physician ○ Rather the physician ○ Difficult to say ○ Rather the AI ○ Definitely the AI
**E. Opinie i postawy**	**E. Opinions and attitudes**
**18. Czy uważa Pan/Pani, że lekarz**	**18. Do you believe physicians should ask**

**powinien pytać pacjentów, czy korzystali z AI przed wizytą?** ◯ Tak ◯ Nie ◯ Nie wiem	**patients whether they used AI before the visit?** ◯ Yes ◯ No ◯ I don't know
**19. Czy uważa Pan/Pani, że placówki medyczne powinny edukować pacjentów, jak bezpiecznie korzystać z AI w sprawach zdrowotnych?** ◯ Tak ◯ Nie ◯ Nie wiem	**19. Do you believe healthcare facilities should educate patients on how to safely use AI for health-related matters?** ◯ Yes ◯ No ◯ I don't know
**F. Dane demograficzne**	**F. Demographic data**
**20. Jak ocenia Pan/Pani swoje umiejętności korzystania z narzędzi cyfrowych i internetu?** 1 – bardzo niskie 2 3 4 5 – bardzo wysokie	**20. How do you rate your skills in using digital tools and the internet?** 1 – very low 2 3 4 5 – very high
**21. Wiek** ◯ 18–24 ◯ 25–34 ◯ 35–44 ◯ 45–54 ◯ 55–64 ◯ 65 lub więcej	**21. Age** ◯ 18–24 ◯ 25–34 ◯ 35–44 ◯ 45–54 ◯ 55–64 ◯ 65 or older
**22. Płeć** ◯ Kobieta ◯ Mężczyzna ◯ Inna ◯ Wolę nie odpowiadać	**22. Gender** ◯ Woman ◯ Man ◯ Other ◯ Prefer not to answer
**23. Wykształcenie** ◯ Podstawowe ◯ Zawodowe ◯ Średnie ◯ Wyższe ◯ Wolę nie odpowiadać	**23. Education** ◯ Primary ◯ Vocational ◯ Secondary ◯ Higher ◯ Prefer not to answer
**24. Jak często korzysta Pan/Pani z opieki medycznej?** ◯ Kilka razy w roku lub rzadziej ◯ Raz na kilka miesięcy ◯ Raz w miesiącu ◯ Kilka razy w miesiącu ◯ Bardzo często	**24. How frequently do you use medical care?** ◯ A few times a year or less ◯ Once every few months ◯ Once a month ◯ Several times a month ◯ Very frequently

Note (routing as implemented in the survey platform and confirmed by the complete data export). Items 2–14 were administered only to respondents who selected "Yes" on Item 1; respondents who selected "No" or "I don't know / don't remember" on Item 1 skipped Items 2–14 and continued from Item 15. Within Item 1 = Yes, Items 5–9 were administered only to respondents who selected "Yes" on Item 4 (respondents selecting "No" or "I don't remember" on Item 4 skipped Items 5–9), Item 12 only to respondents who selected "Yes" on Item 11, and Item 14 only to respondents who selected "No" on Item 13. Items 15–24 were displayed to all respondents. Item 16 is analyzed among respondents who used AI (Item 1 = Yes, n=880), for whom it is meaningful; no respondent without AI use gave an affirmative response. An earlier version of this document instructed Item 1 non-users to proceed to Item 18; this was a documentation error, and the implemented route was to Item 15, as confirmed by the response export. The survey's internal working title ("The patient after a chatbot consultation…") and its stated completion time of 5–7 minutes differ from the final manuscript title and the approximate 10-minute unaided pretest estimate; the instrument text above reproduces what participants actually saw. "Anonymous" denotes anonymity with respect to the investigators: the platform required a Google sign-in to limit duplicate submissions, but email addresses were not collected and no identifiers entered the research dataset.

Supplementary Table S1. Sample characteristics (N = 1044)

Manuscript: Generative AI use before medical visits: disclosure-item responses, trust, and care-seeking behaviors in a cross-sectional social-media survey in Poland

**Characteristic**	**Category**	**n (%) or mean (SD)**
Gender (Q22)	Woman	572 (54.8%)
	Man	422 (40.4%)
	Prefer not to answer	50 (4.8%)
Age group (Q21)	18–24	141 (13.5%)
	25–34	296 (28.4%)
	35–44	265 (25.4%)
	45–54	172 (16.5%)
	55–64	122 (11.7%)
	65+	48 (4.6%)
Education (Q23)	Primary	7 (0.7%)
	Vocational	229 (21.9%)
	Secondary	622 (59.6%)
	Higher	186 (17.8%)
Digital competency (Q20, 1–5)	Overall	4.08 (0.84)
AI trust (Q15, 1–5)	Overall	3.25 (1.06)
Healthcare utilization frequency (Q24, 1–5)	Overall	2.46 (1.27)
	Women	2.85 (1.27)
	Men	1.96 (1.10)
AI use frequency (Q2, 1–4; base Q1=Yes, n=880)	Overall	2.33 (0.65)
	Women	2.24 (0.63)
	Men	2.48 (0.66)
Ever used AI for health	Yes	880 (84.3%)

purposes (Q1)		
	No	94 (9.0%)
	Don't know / don't remember	70 (6.7%)
Pre-consultation AI use (Q4; base Q1=Yes, n=880)	Yes	519 (59.0%)
	No	297 (33.8%)
	Don't remember	64 (7.3%)

Note: Percentages are computed on N = 1044 unless a different base is indicated. Gender-stratified values exclude respondents who selected "prefer not to answer". All values computed directly from the study dataset with the analysis code provided as Supplementary Material.

Supplementary Table S2. Benjamini–Hochberg (FDR) sensitivity analyses

Manuscript 1933451 — Frontiers in Digital Health. Benjamini–Hochberg step-up false-discovery-rate adjustment applied within each pre-defined family of tests as a sensitivity analysis; the primary interpretation of all analyses remains descriptive and exploratory. Exact p-values are computed from the study dataset with the deposited analysis code.

Panel A. Gender-associated comparisons (one family; m = 8 tests)

Comparison	Test	Raw p	BH-adjusted q	Sig. at FDR 0.05
Gender × visit avoidance (Q6; definite Yes/No)	Chi-square	3.4×10^{-8}	5.4×10^{-8}	Yes
Gender × any AI-associated behavior change (Q11; definite)	Chi-square	5.1×10^{-9}	1.0×10^{-8}	Yes
Gender × additional testing and/or second opinion (Q10 composite)	Chi-square	1.5×10^{-17}	4.0×10^{-17}	Yes
Gender × additional testing, any (Q10)	Chi-square	2.0×10^{-7}	2.7×10^{-7}	Yes
Gender × second-opinion request, any (Q10)	Chi-square	1.5×10^{-6}	1.5×10^{-6}	Yes
Gender × increase in health anxiety (Q9)	Mann–Whitney U	5.1×10^{-21}	2.1×10^{-20}	Yes
Gender × healthcare-utilization frequency (Q24; full sample)	Mann–Whitney U	2.0×10^{-28}	1.6×10^{-27}	Yes
Gender × AI-use frequency (Q2)	Mann–Whitney U	3.2×10^{-7}	3.6×10^{-7}	Yes

All eight comparisons form a single Benjamini–Hochberg family and all remain significant after adjustment. Q24 uses the full sample because that item was displayed to all respondents; the AI-user-restricted Q24 comparison (U=117,062.5, r_rb=+0.368) is a further sensitivity analysis reported in the main text.

Panel B. Primary definite-response regression coefficients (within-model BH adjustment)

Benjamini–Hochberg adjustment applied within each model across its coefficients (intercept excluded). Q6 model: N=409, 139 events. Q11 model: N=759, 366 events. OR = odds ratio.

Predictor	Q6 OR	Q6 raw p	Q6 BH q	Q11 OR	Q11 raw p	Q11 BH q
Gender: male (ref: woman)	2.98	1.2×10^{-5}	1.4×10^{-4}	2.40	2.2×10^{-7}	2.6×10^{-6}
AI trust (Q15, per point)	1.90	0.0017	0.010	1.56	4.9×10^{-4}	0.0030
Age 18–24 (ref: 25–34)	0.96	0.914	0.929	0.83	0.428	0.734
Age 35–44 (ref: 25–34)	1.98	0.065	0.155	0.95	0.834	0.910
Age 45–54 (ref: 25–34)	1.33	0.584	0.876	0.87	0.677	0.879
Age 55–64 (ref: 25–34)	3.94	0.016	0.064	0.96	0.911	0.911

Predictor	Q6 OR	Q6 raw p	Q6 BH q	Q11 OR	Q11 raw p	Q11 BH q
Age 65+ (ref: 25–34)	0.81	0.823	0.929	1.22	0.720	0.879
AI-use frequency (Q2, per category)	0.94	0.811	0.929	1.23	0.216	0.518
Digital competency (Q20, per point)	0.98	0.929	0.929	0.87	0.306	0.613
Healthcare utilization (Q24, per category)	0.80	0.025	0.076	1.02	0.732	0.879
Education: primary/vocational (ref: secondary)	1.66	0.105	0.211	0.77	0.212	0.518
Education: higher (ref: secondary)	1.28	0.416	0.713	0.70	0.087	0.347

In both models, male gender and AI trust remain significant after within-model BH adjustment. The unadjusted association between lower healthcare utilization and visit avoidance (Q6, raw p=0.025) does not survive adjustment (q=0.076), and the single Age 55–64 contrast in the Q6 model (raw p=0.016) does not survive adjustment (q=0.064); neither is interpreted as an independent effect. Global likelihood-ratio tests for the multi-level covariates were non-significant (Q6 age p=0.056, education p=0.231; Q11 age p=0.952, education p=0.143).

Panel C. Outcome-coding sensitivity (legacy coding retained from the original submission)

As an additional sensitivity analysis of outcome coding, both models were also estimated with “don’t remember” and “prefer not to answer” coded as no reported occurrence (Q6: N=492, 139 events; Q11: N=838, 366 events). Male gender (Q6 OR=2.70, 95% CI 1.69–4.31; Q11 OR=2.57, 95% CI 1.87–3.53) and AI trust (Q6 OR=1.91, 95% CI 1.31–2.77; Q11 OR=1.52, 95% CI 1.19–1.94) remained significant, yielding the same substantive conclusions as the primary definite-response models. An exact-duplicate one-record-removed sensitivity analysis left both primary models unchanged.

Method note. *Benjamini–Hochberg step-up adjustment was applied separately within (i) the eight gender-comparison tests (Panel A) and (ii) the coefficients of each primary regression model (Panel B). Exploratory Spearman correlations of AI trust with age, AI-use frequency, digital competency, and anxiety were each p<0.001 and are reported descriptively in the main text. The primary interpretation of all analyses remains descriptive and exploratory, as stated in the Materials and methods section.*

CHERRIES Checklist

Manuscript 1933451 | prepared from the 5 August 2026 revision package

Page and section references correspond to the current rendered manuscript and were verified against the final manuscript.

Manuscript title: Generative AI use before medical visits: disclosure-item responses, trust, and care-seeking behaviors in a cross-sectional social-media survey in Poland

Guideline: Eysenbach G. Improving the Quality of Web Surveys: CHERRIES. J Med Internet Res. 2004;6(3):e34; correction 2012;14(1):e8.

Category	CHERRIES item	Final report/response	Location	Status
Design	Survey design	Cross-sectional, self-administered online survey of adults aged ≥18 years residing in Poland. Non-probability convenience sample recruited through Instagram; not representative of Polish adults.	Methods 2.1, pp. 3–4	Complete
Ethics and consent	Ethics/IRB status	Formal ethics-committee approval and an independent exemption determination were not obtained before fieldwork. The authors assessed the survey as outside the statutory definition of a medical experiment; no institutional opinion has been obtain ed . This remains an unresolved administrative limitation and is not retrospective approval.	Methods 2.5, p. 7; Ethics statement, p. 13	No independent institutional determination obtained; submitted for editorial assessment
Ethics and consent	Informed-consent process	Before the questionnaire, participants viewed Polish study information, eligibility declarations, voluntariness/withdrawal information, data-handling information, and a required digital-consent gate. Selecting non-consent terminated the survey.	Methods 2.2, pp. 4–5; Supplementary File I	Complete
Ethics and consent	Survey length disclosed	The participant-facing survey introduction stated 5–7 minutes. A later two-stage pretest estimated unaided completion at approximately 10 minutes. Both values are reported with their source and are not treated as interchangeable.	Supplementary Material S1; Methods 2.2, pp. 4–5; File J	Complete

Category	CHERRIES item	Final report/response	Location	Status
Ethics and consent	Data protection	The survey was anonymous with respect to the investigators. Google-account sign-in was required because the form limited submissions to one response per account; e-mail collection was disabled and no names/e-mails/direct identifiers were present in the research dataset. Google could process account/technical data outside the investigators' dataset.	Methods 2.2, p. 5; Supplementary File I	Complete
Development/pretesting	Instrument development	Author-developed 24-item Polish questionnaire. Reviewed by one expert in medical communication and one in digital health. Two-stage pretest: 15 Teams sessions with concurrent verbal probing, iterative revision, then 15 independent online completions; ages 18–72; no compensation; participants excluded from the main sample. No formal psychometric validation.	Methods 2.2, pp. 4–5; File J	Complete — psychology (medical-communication reviewer) and economics (digital-health reviewer)
Recruitment	Open or closed survey	Open dissemination of one responder link through Instagram, but submission required a Google-account sign-in because the one-response setting was enabled.	Methods 2.1–2.2, pp. 3–5	Complete
Recruitment	Initial contact mode	Five invitations were posted on the first author's Instagram account on 1, 4, 7, 10, and 13 March 2026. Operators of 15 independently managed Instagram accounts were directly asked to share the invitation; 101 unique Instagram accounts shared it organically.	Methods 2.1, pp. 3–4; File K	Complete

Category	CHERRIES item	Final report/response	Location	Status
Recruitment	Advertising and invitation content	Instagram primarily. One low-reach p ost on Fa cebook and one on L inkedIn , no paid advertising/boostin g, and no compensation to respondents or account owners. Posts described the topic but did not disclose hypotheses, expected findings, or preferred answers.	Methods 2.1, pp. 3–4; File K	Complete
Administration	Mode and language	Self-administered Google Forms survey, displayed exclusively in Polish. The English version was prepared after data collection for reporting only and was not used in recruitment or fieldwork.	Methods 2.2, pp. 4–5; Supplementary Material S1	Complete
Administration	Voluntary/mandatory participation	Participation was voluntary. Eligibility and consent declarations were required to enter the questionnaire. No incentives were offered.	Methods 2.1–2.2, pp. 3–5; File I	Complete
Administration	Data-collection period	1 March–15 April 2026.	Methods 2.1, p. 3	Complete
Administration	Item order/randomization	Fixed item and response-option order; no randomization. The potential for order, acquiescence, and social-desirability effects is acknowledged, particularly for Q14 and Q17.	Methods 2.2 and Limitations, pp. 4–5 and 12	Complete
Administration	Adaptive questioning	Four programmed gates: Q1 controlled Q2–Q14; Q4 controlled Q5–Q9; Q11 controlled Q12; Q13=No triggered Q14. Q15–Q24 were displayed to all. No routing violations were found.	Methods 2.2, p. 5; Supplementary Figure S1; File D	Complete
Administration	Number of items/screens	The questionnaire contained 24 numbered items plus participant-information, eligibility, and consent screens. The number of questionnaire screens varied by routing and is not recoverable as one fixed value from the export.	Methods 2.2, pp. 4–5; S1; File I	Complete

Category	CHERRIES item	Final report/response	Location	Status
Administration	Completeness check	Only submitted questionnaires entered the dataset. The export showed complete responses for displayed analytic items and structural non-display (N/D) for routed items. Response settings are documented in Supplementary File I-b; item-level required/not-required settings are not separately documented.	Methods 2.2, p. 5; File D	Complete with one technical setting available only from archive
Administration	Review/change answers	Respondents could navigate and revise answers before submission. Editing after submission was disabled.	Methods 2.2, p. 5; platform settings record	Complete
Response metrics	Unique visitor definition	Not available. Recruitment-post exposure and unique survey-visitor analytics were not collected in a form that could be linked to survey participation.	Methods 2.1–2.2, pp. 3–5	Not determinable
Response metrics	View rate	Not determinable because the number of unique people exposed to the invitations and the number opening the survey were unavailable.	CHERRIES; Methods 2.2, p. 5	Not determinable
Response metrics	Participation rate	Not determinable because unique survey visitors/consent-page visitors were not recorded.	CHERRIES; Methods 2.2, p. 5	Not determinable
Response metrics	Completion rate	N=1,044 submitted questionnaires were analyzed. Started-but-not-submitted sessions were not included in the response export; therefore the CHERRIES completion-rate denominator is unavailable. This uses the corrected CHERRIES definition.	Methods 2.2, p. 5	Not determinable
Preventing multiple entries	Cookies	No investigator-controlled cookie-based duplicate-prevention mechanism was documented. Google Forms’ one-response-per-account setting was used instead.	Methods 2.2, p. 5	Complete
Preventing multiple entries	Account/registration requirement	Yes. Google-account sign-in was required to enforce one response per account; collection of e-mail addresses was disabled.	Methods 2.2, p. 5	Complete
Preventing multiple entries	IP/log checks	IP address, device, user-agent, detailed timestamps, and source-account identifiers were not available in the research export. They were not used for exclusions.	Methods 2.4 and Limitations, pp. 7 and 12; File L	Complete limitation

Category	CHERRIES item	Final report/response	Location	Status
Preventing multiple entries	Duplicate-pattern analysis	One exact matching short-route response vector was detected; no qualifying near-duplicate pairs were found under the prespecified similarity rule. The pair did not enter the Q6/Q11 models; one-record-removed sensitivity analysis did not alter conclusions.	Methods 2.4, p. 7; Files A and L	Complete
Analysis	Incomplete questionnaires	Only submitted questionnaires were analyzed. Routed N/D values were treated as structural non-display, not respondent-level missingness.	Methods 2.2–2.4, pp. 5–7; File D	Complete
Analysis	Atypical response time/bot screening	Completion-time, IP, device/user-agent, and dedicated bot-detection data were not available; no timestamp-based exclusions were possible. Additional structural checks found no routing violations, broad straightlining, serial clustering, or near-duplicate pattern meeting the prespecified rule.	Methods 2.4 and Limitations, pp. 7 and 12; File L	Complete with unavoidable limitation
Analysis	Handling non-representativeness	Primary analyses are unweighted and exploratory. No weighting can make the convenience sample representative. Optional age/gender/education standardization is reported only as descriptive sensitivity; the education-calibrated scenario is unstable because primary education n=7.	Methods 2.1, 2.4, Limitations; File O	Complete

STROBE Cross-Sectional Checklist

Manuscript 1933451 | prepared from the 5 August 2026 revision package

This checklist reflects the revised definite-response models, categorical age and education terms, the primary Q1=Yes trust analysis, the missingness and model-stability supplements, and the final Q24/FDR specification. Page references were verified against the final manuscript file .

Guideline: STROBE Statement — checklist for cross-sectional studies.

Section	Item	Recommendation	Final report/response	Location	Status
Title/abstract	1a	Identify the design	The title identifies a cross-sectional social-media survey; the Abstract Methods states cross-sectional online survey.	Title/Abstract, pp. 1–2	Complete
Title/abstract	1b	Balanced abstract	Structured Background, Methods, Results, and Conclusions report the convenience sample, routed denominators, main findings, lack of confirmed attendance, and non-generalizability.	Abstract, pp. 1–2	Complete
Introduction	2	Background/rationale	Describes patient-facing AI use and uses NEM, automation bias, anthropomorphism, and cyberchondria only as interpretive frameworks, not measured constructs.	Introduction, pp. 2–3	Complete; Complete
Introduction	3	Objectives/ hypotheses	Four exploratory objectives are stated; no a priori hypotheses were preregistered.	Introduction, p. 3	Complete
Methods	4	Study design	Cross-sectional online survey described at the beginning of Methods.	Methods 2.1, p. 3	Complete
Methods	5	Setting and dates	Poland; Instagram-based recruitment; 1 March–15 April 2026; five first-author post dates and broader dissemination described.	Methods 2.1, pp. 3–4	Complete
Methods	6a	Eligibility and selection	Adults ≥18 years residing in Poland; convenience recruitment through Instagram; one common Google Forms link; respondent and account-owner compensation absent.	Methods 2.1–2.2, pp. 3–5	Complete
Methods	6b	Matched-study criteria	Not applicable; no matching was used.	Not applicable	N/A

Section	Item	Recommendation	Final report/response	Location	Status
Methods	7	Variables	Primary/secondary outcomes and covariates are mapped to Q1–Q24; Q13 and Q6 interpretation boundaries are stated; Q11 is treated as a heterogeneous behavior-change outcome.	Methods 2.3, pp. 5–6; Instrument Fidelity Matrix	Complete
Methods	8	Data sources/measurement	Author-developed Polish questionnaire, expert review, two-stage pretest, exact item wording/translation, routing, scale coding, and limitations of single-item measures are described.	Methods 2.2–2.3, pp. 4–6; S1; Files H/J; Fidelity Matrix	Complete — exact expert qualifications and translator/checker roles remain factual inputs
Methods	9	Bias	Recruitment/self-selection, digital divide, social desirability, fixed option order, common-source self-report, lack of episode anchoring, duplicate/bot-data limitations, denominator selection, sparse cells, and model optimism are addressed. Files L–O provide additional checks.	Methods 2.1–2.4 and Limitations, pp. 3–7 and 12; Files L–O	Complete with unavoidable limitations
Methods	10	Study size	An a priori target of approximately 1,000 completed questionnaires was set for descriptive precision (maximum 95% CI half-width about 3.1 percentage points at p=0.50 for N=1,000). It was not a power calculation for routed subgroups or regression models.	Methods 2.1, p. 4	Complete; retain documentary evidence that target predated recruitment
Methods	11	Quantitative variables	Age and education are categorical in primary models. Q2, Q15, Q20, and Q24 coding direction is reported. Q24 full sample is primary for the gender comparison; Q1=Yes is sensitivity. Functional-form and age-group sensitivities are in File N.	Methods 2.3–2.4, pp. 5–7; File N	Complete

Section	Item	Recommendation	Final report/response	Location	Status
Methods	12a	Statistical methods/confounding	Descriptive estimates with denominators/Wilson CIs; chi-square/Cramér's V; Mann–Whitney U/rank-biserial; Kruskal–Wallis; Spearman; simultaneous multivariable logistic regression. Covariates and reference groups reported. Global LR tests, VIF, AUC, R^2, calibration, and internal validation provided.	Methods 2.4, pp. 6–7; Tables 4–5; Files L/N	Complete to manuscript
Methods	12b	Subgroups/ interactions	Q13 stratified by Q6 response; gender comparisons; Q15 by age; Q16 full-sample and user-only bases. No formal interaction model is claimed unless explicitly reported.	Methods 2.4; Results 3.3–3.7, pp. 6–10	Complete
Methods	12c	Missing data	Structural N/D is distinguished from observed uncertainty/refusal/N/A. Primary Q6/Q11 models use definite responses. Sensitivities include original coding, response-propensity/IPW, and deterministic tipping-point assignments. Q10 N/A handled explicitly.	Methods 2.2–2.4, pp. 5–7; File M	Complete
Methods	12d	Sampling strategy	No design weights in primary analysis because inclusion probabilities are unknown. Convenience sampling and lack of clustering/source-account IDs are acknowledged. Descriptive standardization is sensitivity only and not a population estimate.	Methods 2.1/2.4 and Limitations; File O	Complete
Methods	12e	Sensitivity analyses	Q13 alternative denominators; duplicate removal; Q6/Q11 outcome coding; FDR; Q24 full-sample vs Q1=Yes; data-quality checks; bootstrap internal validation; Firth/ridge/influence/age-form analyses; missingness IPW/tipping; descriptive standardization.	Results 3.3–3.7; S2; Files A/L/M/N/O	Complete
Methods	12f	Cross-sectional design	Analyses use contemporaneous self-reported data; no longitudinal/censoring methods apply.	Methods 2.1–2.4	N/A

Section	Item	Recommendation	Final report/response	Location	Status
Results	13a	Participants at each stage	N=1,044 submitted surveys; Q1=Yes n=880; Q4=Yes n=519; item- and model-specific denominators reported. Routing diagram and denominator table provided.	Results 3.1–3.3, pp. 7–8; Figure 2; Figure S1; File D	Complete
Results	13b	Reasons for non-participation	Recruitment exposure, survey views, consent-page visits, and starts/abandonments were unavailable. Routing exclusions are reported but are not non-participation.	Methods 2.2, p. 5; CHERRIES	Complete
Results	13c	Flow diagram	Figure 2 shows sample/analytic flow; Figure S1 shows programmed routing. Figure 2 should avoid implying that Q6=No confirms attendance.	Figures 2 and S1	Complete
Results	14a	Participant characteristics	Gender, age, education, digital competency and other sample characteristics reported; full table in Supplementary Table S1.	Results 3.1, p. 7; Supplementary Table S1	Complete
Results	14b	Missing data by variable	Observed uncertainty/refusal/N/A and structural N/D are reported by item; File M examines differential response status by group.	Methods 2.2–2.4; Results 3.4; Files D/M	Complete
Results	14c	Cohort follow-up	Not applicable to a cross-sectional survey.	Not applicable	N/A
Results	15	Outcome data	Counts, percentages, denominators and summary measures reported for Q1, Q4, Q5–Q19 and routed outcomes; main results in Tables 1–4 and supplements.	Results 3.2–3.8, pp. 8–11	Complete
Results	16a	Main estimates and precision	Unadjusted group estimates/effect sizes and adjusted ORs with 95% CIs reported. Risk-difference CIs are reported for binary gender outcomes. Apparent and bootstrap-corrected model performance is separated.	Results 3.6–3.7, p. 10; Tables 4–5; Files L/N	Complete
Results	16b	Category boundaries	Age, education, Q2, Q15, Q20, and Q24 categories/scales and reference levels described.	Methods 2.3–2.4, pp. 5–7; Table 5	Complete
Results	16c	Absolute risk translation	Not applicable as a time-based risk analysis; absolute cross-sectional percentages and risk differences are already reported.	Results 3.2–3.7	N/A

Section	Item	Recommendation	Final report/response	Location	Status
Results	17	Other analyses	Q13 denominator sensitivities, trust analysis bases, gender FDR family, full-sample Q24 sensitivity, duplicate sensitivity, data-quality/internal validation, missingness, penalized regression/influence, and standardization are reported.	Results 3.3–3.7; S2; Files A/L/M/N/O	Complete
Discussion	18	Key results	Discussion and conclusion summarize findings against the four objectives and distinguish Q13 response patterns from confirmed encounter disclosure.	Discussion 4.1; Conclusions, pp. 11–13	Complete
Discussion	19	Limitations	Selection, platform, self-report, no episode anchoring/recall period, single-item measurement, no psychometric validation, fixed response order, missing technical metadata, sparse model cells, complete-case selection, model optimism, and no external validation discussed.	Limitations 4.6, p. 12; Files L–O	Complete
Discussion	20	Interpretation	Interpretation is exploratory and must avoid causal language, confirmed non-disclosure, homogeneous care substitution, and theory testing. Alternative explanations are discussed.	Discussion 4.1–4.5, pp. 11–12	Complete — Complete
Discussion	21	Generalisability	Convenience Instagram sample is not representative. Recruitment probabilities are unknown; optional standardization does not restore representativeness.	Methods 2.1, Limitations/Conclusions; File O	Complete
Other	22	Funding	No dedicated research funding; APC paid by LUX MED; employer role stated; employment/affiliations disclosed.	Funding/COI statement, p. 13	Complete subject to Frontiers metadata sync

Supplementary File G. Model performance, internal

Manuscript 1933451. The values in this file correspond to the revised definite-response models and the deposited reproducibility materials. The regression coefficients and FDR analyses are also reported in revised Table 5 and Supplementary Table S2.

G.1 Primary definite-response model performance

Model	N	Events	Apparent AUC	Bootstrap optimism	Optimism-corrected AUC	Nagelkerke R^2	Hosmer–Lemeshow p	Max VIF
Q6 reported visit avoidance	409	139	0.720	0.036	0.684	0.183	0.90 (tie-safe deciles)	3.26 (AI trust)
Q11 any AI-associated behaviour	759	366	0.687	0.024	0.665	0.139	0.72 (tie-safe deciles)	3.01 (AI trust)

The optimism-corrected AUC shown here matches the full internal validation in Supplementary File L, which used 1,000 nonparametric bootstrap resamples and additionally reports Brier score, calibration intercept and slope, and grouped calibration plots. The bootstrap-corrected calibration slopes were 0.793 (Q6) and 0.871 (Q11), and the corrected Nagelkerke R^2 values were 0.096 and 0.099. Discrimination is reported as apparent and optimism-corrected only; neither model was externally validated. The Q6 model failed to refit in a small fraction of resamples, consistent with 139 events across 12 parameters and sparse older-age cells (65+: n=15, 2 events); its individual coefficients, particularly the age contrasts, are unstable exploratory estimates. Hosmer-Lemeshow p-values are not presented as evidence of adequate calibration.

G.2 Differential missingness of uncertain/refusal responses by gender

Item (base)	Women uncertain/NA	Men uncertain/NA	χ^2	p	Interpretation
Q11 behaviour change (Q1=Yes)	58/485 (12.0%)	21/353 (5.9%)	8.64	0.003	Uncertain responses more frequent in women
Q10 testing/2nd opinion — “not applicable” (Q1=Yes)	15/485 (3.1%)	36/353 (10.2%)	18.05	<0.001	“Not applicable” more frequent in men

Q6 visit avoidance (Q4=Yes)	47/280 (16.8%)	36/212 (17.0%)	0.00	0.95	No differential missingness

Because uncertain/“not applicable” responses to Q11 and Q10 are associated with gender — itself a primary predictor — the definite-response/applicable-case handling is not missing-completely-at-random. The direction of the gender differences is preserved under conservative coding (for Q10, counting “not applicable” as a non-event gives women 76.5% vs. men 44.8%, $p<0.001$) and in the legacy-coding models that retain all respondents (Supplementary Table S2, Panel C). These estimates should be read with the differential missingness in mind.

Supplementary File I

Participant Information, Eligibility Confirmation, and Digital Consent Documentation

Frontiers in Digital Health manuscript 1933451

Evidence status: The screenshots reproduced in this file are the participant-facing Google Forms information, eligibility, and digital-consent screens as presented to respondents during the data-collection period (1 March-15 April 2026), confirmed by the corresponding author as the screens used in the fielded survey.

1. What the screenshots establish

Documented element	Evidence from the participant-facing screens
**Study information before consent**	Participants were shown a multi-page Polish-language information module describing the study purpose, procedures, voluntariness, anonymity, data handling, foreseeable discomfort, lack of compensation, contact details, and the medical-advice disclaimer.
**Age eligibility**	A required participant declaration explicitly confirmed: “mam ukończone 18 lat” (I am at least 18 years old).
**Residence eligibility**	A required participant declaration explicitly confirmed: “mieszkam w Polsce” (I live in Poland).
**Digital consent gate**	A required Yes/No item asked whether the person consented to participate. The form stated that selecting “I do not consent” would terminate the survey.
**Mandatory participant declaration**	The participant had to confirm reading the study information, understanding the purpose and rules, voluntariness, the right to stop before submission, the possible impossibility of withdrawal after anonymous submission, and consent to participation and analysis of responses.
**No direct identifiers**	The participant information stated that names, surnames, PESEL numbers, residential addresses, telephone numbers, email addresses, and medical-record data were not collected.
**Platform-level processing**	Participants were informed that Google could process technical data in accordance with the rules applicable to Google services.
**Compensation and risk**	No medical or financial benefit and no remuneration were offered. The information acknowledged that some items could recall health-related situations, anxiety, or decision-making.
**Withdrawal**	Participants could stop before submitting the form. Because the survey responses were anonymous, later withdrawal of an already submitted response might not be possible.
**Data sharing**	Possible sharing of anonymized data with other researchers was described as conditional on assessment of the request, consistency with the scope of consent, and appropriate safeguards. The screen did not describe unrestricted public release of individual-level data.

2. Manuscript implications

2.1 Methods – eligibility, information, and consent

Manuscript wording (English):

Before entering the questionnaire, participants viewed a multi-page Polish-language information module describing the study purpose, procedures, voluntariness, anonymity with respect to the investigators, potential discomfort, data handling, and contact details. Eligibility was confirmed through required declarations that the respondent was aged at least 18 years and lived in Poland. Participants were required to confirm that they had read and understood the study information, understood that participation was voluntary, knew that they could discontinue before submitting the form, understood that withdrawal after anonymous submission might not be possible, and consented to participation and analysis of their responses. A required binary consent item was also used; selecting non-consent terminated the survey. No names, email addresses, national identification numbers, contact details, residential addresses, or medical-record data were collected in the research dataset.

2.2 Platform authentication and anonymity wording

Because Google Forms was configured to limit submissions to one response per Google account, respondents were required to sign in to Google; however, email-address collection was disabled and no account identifiers were available to the investigators. The most precise description is therefore "anonymous with respect to the investigators" or "no direct identifiers were collected in the research dataset", rather than an unqualified claim of technical anonymity at the platform level.

2.3 Data availability

Data sharing: The participant information supports controlled sharing of de-identified individual-level data after assessment of a request; it does not support unrestricted public release. Openly deposited materials (code, aggregate outputs, questionnaire, routing, verification code) are in the repository (https://doi.org/10.17605/OSF.IO/7Z6CW).

Recommended Data Availability Statement:

The individual-level dataset is not openly deposited because the participant information described potential sharing with other researchers as conditional on assessment of a request, compatibility with the scope of consent, and appropriate safeguards, rather than unrestricted public release. The questionnaire documentation, participant-information and consent screens, routing materials, analysis code, aggregate response distributions, figure-source data, and statistical outputs are available at https://doi.org/10.17605/OSF.IO/7Z6CW. De-identified individual-level data may be considered upon reasonable request to the corresponding author, subject to compatibility with the original consent, institutional assessment, a justified scientific purpose, appropriate data-security safeguards, and a prohibition on re-identification or onward sharing.

2.4 Response to reviewers

Response provided :

We recovered and documented the complete participant-facing information and digital-consent sequence. The revised Methods now specifies that eligibility was confirmed through required declarations of age ≥18 years and residence in Poland, and that participants had to confirm reading and understanding the study information, voluntariness, the right to stop before submission, and consent to participation and analysis. We also clarified that the survey was anonymous with respect to the investigators: Google-account sign-in was required to enforce the one-response setting, but email-address collection was disabled and no direct identifiers were available in the research dataset. The screens are reproduced in Supplementary File I. In addition, the Data Availability Statement was aligned with the actual participant information: individual-level data are not openly deposited and may be considered only through controlled access consistent with the consent and appropriate safeguards.

3. Remaining limitation

The screenshots establish the information presented and the required declarations in the documented form configuration. They do not by themselves establish how many people viewed the information but did not proceed, because Google Forms did not retain a denominator of visitors, starts, or non-submitted forms. View rate, participation rate, and completion rate therefore remain unavailable for CHERRIES reporting.

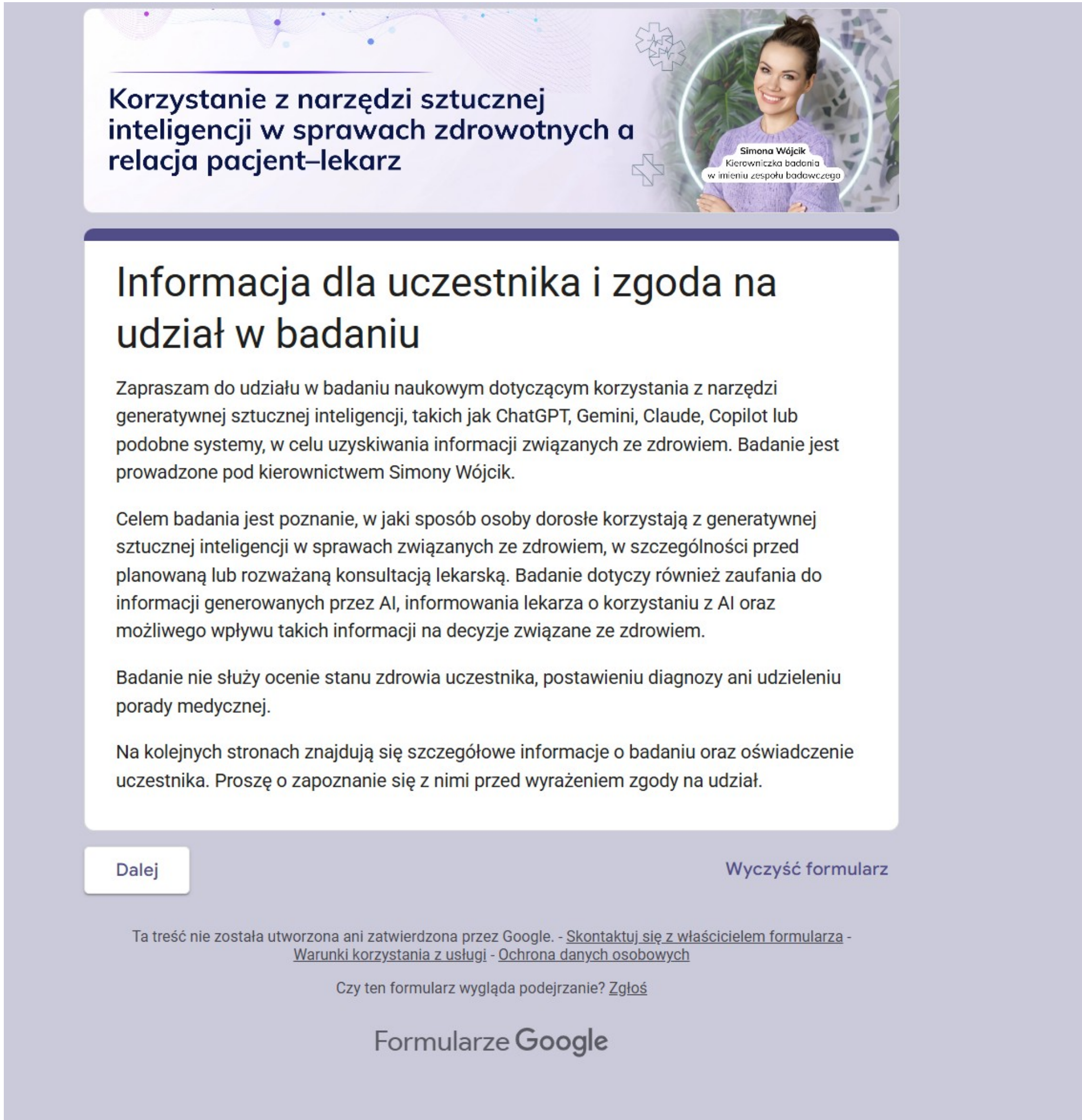

Korzystanie z narzędzi sztucznej inteligencji w sprawach zdrowotnych a relacja pacjent–lekarz

Simona Wójcik
Kierowniczka badania
w imieniu zespołu badawczego

Informacja dla uczestnika i zgoda na udział w badaniu

Zapraszam do udziału w badaniu naukowym dotyczącym korzystania z narzędzi generatywnej sztucznej inteligencji, takich jak ChatGPT, Gemini, Claude, Copilot lub podobne systemy, w celu uzyskiwania informacji związanych ze zdrowiem. Badanie jest prowadzone pod kierownictwem Simony Wójcik.

Celem badania jest poznanie, w jaki sposób osoby dorosłe korzystają z generatywnej sztucznej inteligencji w sprawach związanych ze zdrowiem, w szczególności przed planowaną lub rozważaną konsultacją lekarską. Badanie dotyczy również zaufania do informacji generowanych przez AI, informowania lekarza o korzystaniu z AI oraz możliwego wpływu takich informacji na decyzje związane ze zdrowiem.

Badanie nie służy ocenie stanu zdrowia uczestnika, postawieniu diagnozy ani udzieleniu porady medycznej.

Na kolejnych stronach znajdują się szczegółowe informacje o badaniu oraz oświadczenie uczestnika. Proszę o zapoznanie się z nimi przed wyrażeniem zgody na udział.

Dalej

Wyczyść formularz

Ta treść nie została utworzona ani zatwierdzona przez Google. - Skontaktuj się z właścicielem formularza - Warunki korzystania z usługi - Ochrona danych osobowych

Czy ten formularz wygląda podejrzanie? Zgłoś

Formularze Google

Figure I1. Opening participant-information screen.

The opening page identifies the study topic and explains that the survey concerns generative-AI use for health information, especially before a medical visit, trust in AI-generated information, physician disclosure, and possible effects on health decisions. It also states that the survey is not a health assessment, diagnosis, or medical advice.

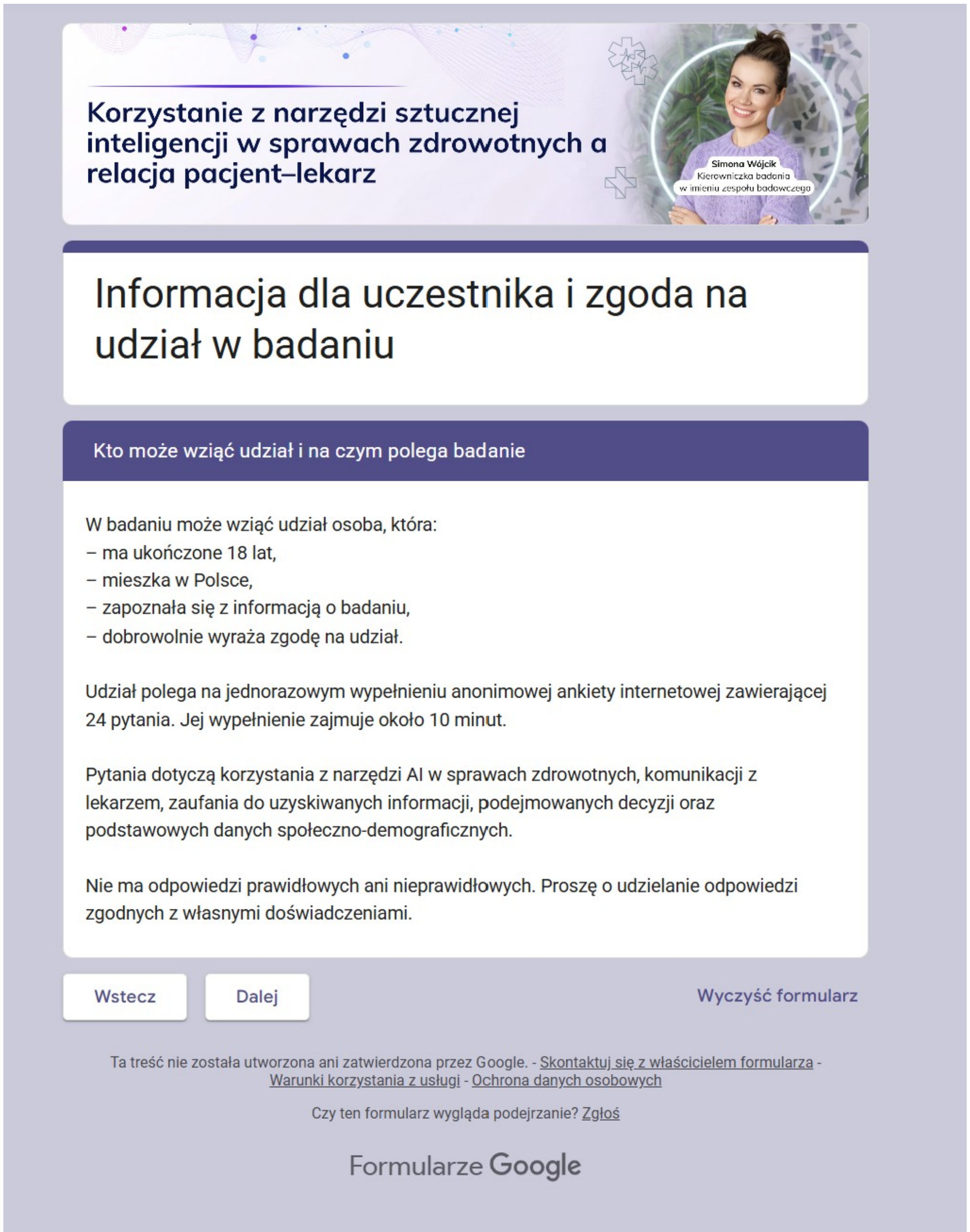

Korzystanie z narzędzi sztucznej inteligencji w sprawach zdrowotnych a relacja pacjent–lekarz

Simona Wójcik
Kierowniczka badania
w imieniu zespołu badawczego

Informacja dla uczestnika i zgoda na udział w badaniu

Kto może wziąć udział i na czym polega badanie

W badaniu może wziąć udział osoba, która:
– ma ukończone 18 lat,
– mieszka w Polsce,
– zapoznała się z informacją o badaniu,
– dobrowolnie wyraża zgodę na udział.

Udział polega na jednorazowym wypełnieniu anonimowej ankiety internetowej zawierającej 24 pytania. Jej wypełnienie zajmuje około 10 minut.

Pytania dotyczą korzystania z narzędzi AI w sprawach zdrowotnych, komunikacji z lekarzem, zaufania do uzyskiwanych informacji, podejmowanych decyzji oraz podstawowych danych społeczno-demograficznych.

Nie ma odpowiedzi prawidłowych ani nieprawidłowych. Proszę o udzielanie odpowiedzi zgodnych z własnymi doświadczeniami.

Wstecz | Dalej | Wyczyść formularz

Ta treść nie została utworzona ani zatwierdzona przez Google. - Skontaktuj się z właścicielem formularza - Warunki korzystania z usługi - Ochrona danych osobowych

Czy ten formularz wygląda podejrzanie? Zgłoś

Formularze Google

Figure I2. Eligibility and study procedures.

The screen states that participants must be aged 18 years or older, live in Poland, have read the study information, and voluntarily consent. It describes a one-time anonymous online survey of 24 questions taking approximately 10 minutes and instructs respondents to answer according to their own experience.

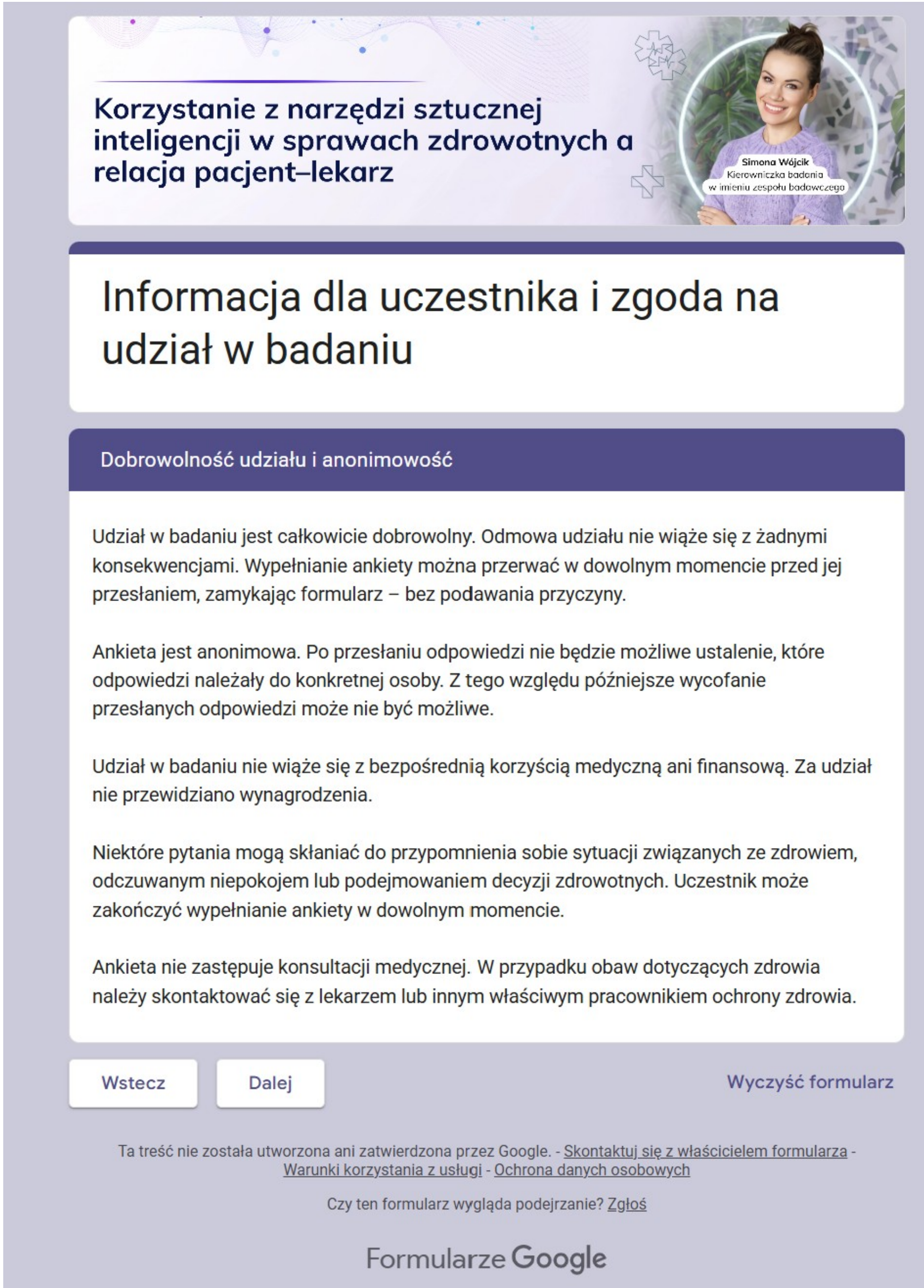

Korzystanie z narzędzi sztucznej inteligencji w sprawach zdrowotnych a relacja pacjent–lekarz

Simona Wójcik
Kierowniczka badania
w imieniu zespołu badawczego

Informacja dla uczestnika i zgoda na udział w badaniu

Dobrowolność udziału i anonimowość

Udział w badaniu jest całkowicie dobrowolny. Odmowa udziału nie wiąże się z żadnymi konsekwencjami. Wypełnianie ankiety można przerwać w dowolnym momencie przed jej przesłaniem, zamykając formularz – bez podawania przyczyny.

Ankieta jest anonimowa. Po przesłaniu odpowiedzi nie będzie możliwe ustalenie, które odpowiedzi należały do konkretnej osoby. Z tego względu późniejsze wycofanie przesłanych odpowiedzi może nie być możliwe.

Udział w badaniu nie wiąże się z bezpośrednią korzyścią medyczną ani finansową. Za udział nie przewidziano wynagrodzenia.

Niektóre pytania mogą skłaniać do przypomnienia sobie sytuacji związanych ze zdrowiem, odczuwanym niepokojem lub podejmowaniem decyzji zdrowotnych. Uczestnik może zakończyć wypełnianie ankiety w dowolnym momencie.

Ankieta nie zastępuje konsultacji medycznej. W przypadku obaw dotyczących zdrowia należy skontaktować się z lekarzem lub innym właściwym pracownikiem ochrony zdrowia.

Wstecz | Dalej | Wyczyść formularz

Ta treść nie została utworzona ani zatwierdzona przez Google. - Skontaktuj się z właścicielem formularza - Warunki korzystania z usługi - Ochrona danych osobowych

Czy ten formularz wygląda podejrzanie? Zgłoś

Formularze Google

Figure I3. Voluntariness, anonymity, withdrawal, lack of compensation, and potential discomfort.

The screen states that participation is voluntary, refusal has no consequences, and the form may be closed before submission. It explains that later withdrawal may be impossible because submitted answers cannot be linked to a person, that no compensation is provided, and that some questions may recall health-related anxiety or decisions.

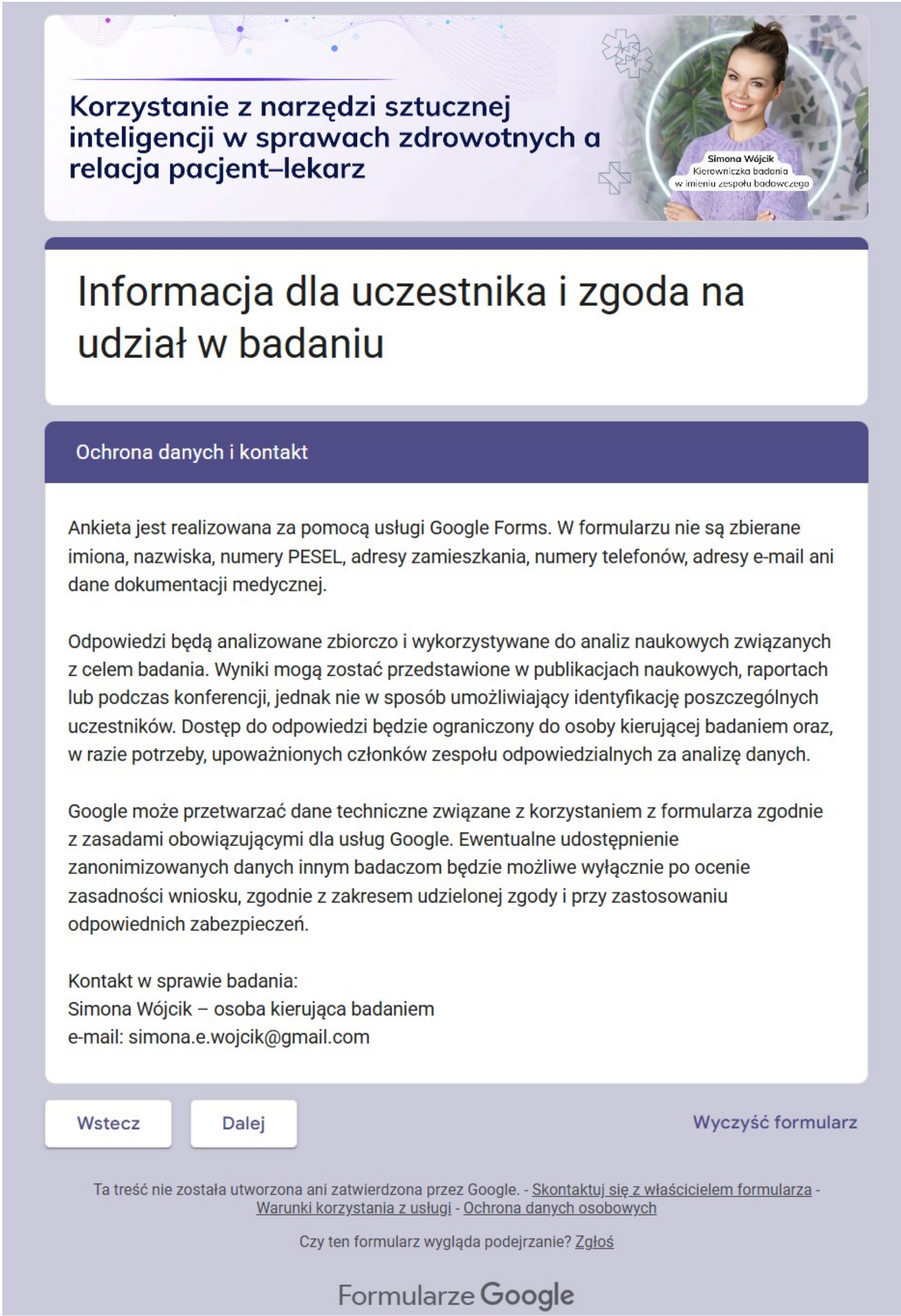

Korzystanie z narzędzi sztucznej inteligencji w sprawach zdrowotnych a relacja pacjent–lekarz

Simona Wójcik
Kierowniczka badania
w imieniu zespołu badawczego

Informacja dla uczestnika i zgoda na udział w badaniu

Ochrona danych i kontakt

Ankieta jest realizowana za pomocą usługi Google Forms. W formularzu nie są zbierane imiona, nazwiska, numery PESEL, adresy zamieszkania, numery telefonów, adresy e-mail ani dane dokumentacji medycznej.

Odpowiedzi będą analizowane zbiorczo i wykorzystywane do analiz naukowych związanych z celem badania. Wyniki mogą zostać przedstawione w publikacjach naukowych, raportach lub podczas konferencji, jednak nie w sposób umożliwiający identyfikację poszczególnych uczestników. Dostęp do odpowiedzi będzie ograniczony do osoby kierującej badaniem oraz, w razie potrzeby, upoważnionych członków zespołu odpowiedzialnych za analizę danych.

Google może przetwarzać dane techniczne związane z korzystaniem z formularza zgodnie z zasadami obowiązującymi dla usług Google. Ewentualne udostępnienie zanonimizowanych danych innym badaczom będzie możliwe wyłącznie po ocenie zasadności wniosku, zgodnie z zakresem udzielonej zgody i przy zastosowaniu odpowiednich zabezpieczeń.

Kontakt w sprawie badania:
Simona Wójcik – osoba kierująca badaniem
e-mail: simona.e.wojcik@gmail.com

Wstecz | Dalej | Wyczyść formularz

Ta treść nie została utworzona ani zatwierdzona przez Google. - Skontaktuj się z właścicielem formularza - Warunki korzystania z usługi - Ochrona danych osobowych

Czy ten formularz wygląda podejrzanie? Zgłoś

Formularze Google

Figure I4. Data protection, controlled sharing, and study contact.

The screen lists categories of direct identifiers that were not collected, states that results may be published only without identifying participants, limits access to the study lead and authorized team members, discloses Google technical-data processing, and describes data sharing with other researchers as conditional on request assessment, consent compatibility, and safeguards.

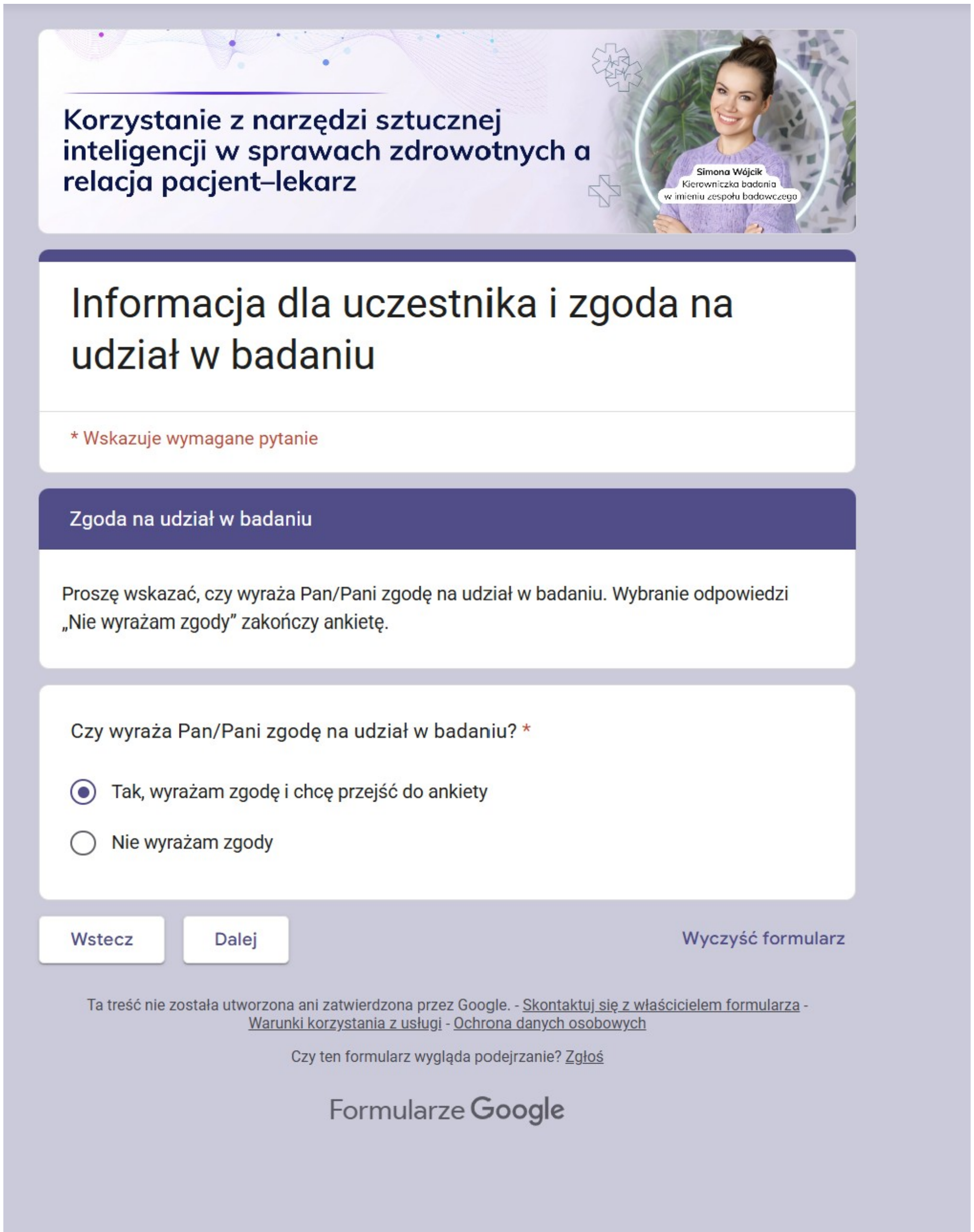

Korzystanie z narzędzi sztucznej inteligencji w sprawach zdrowotnych a relacja pacjent–lekarz

Simona Wójcik
Kierowniczka badania
w imieniu zespołu badawczego

Informacja dla uczestnika i zgoda na udział w badaniu

* Wskazuje wymagane pytanie

Zgoda na udział w badaniu

Proszę wskazać, czy wyraża Pan/Pani zgodę na udział w badaniu. Wybranie odpowiedzi „Nie wyrażam zgody" zakończy ankietę.

Czy wyraża Pan/Pani zgodę na udział w badaniu? *

- (•) Tak, wyrażam zgodę i chcę przejść do ankiety
- ( ) Nie wyrażam zgody

Wstecz | Dalej | Wyczyść formularz

Ta treść nie została utworzona ani zatwierdzona przez Google. - Skontaktuj się z właścicielem formularza - Warunki korzystania z usługi - Ochrona danych osobowych

Czy ten formularz wygląda podejrzanie? Zgłoś

Formularze Google

Figure I5. Required binary consent gate.

A required Yes/No question asks whether the person consents to participate. The form explicitly states that selecting non-consent terminates the survey.

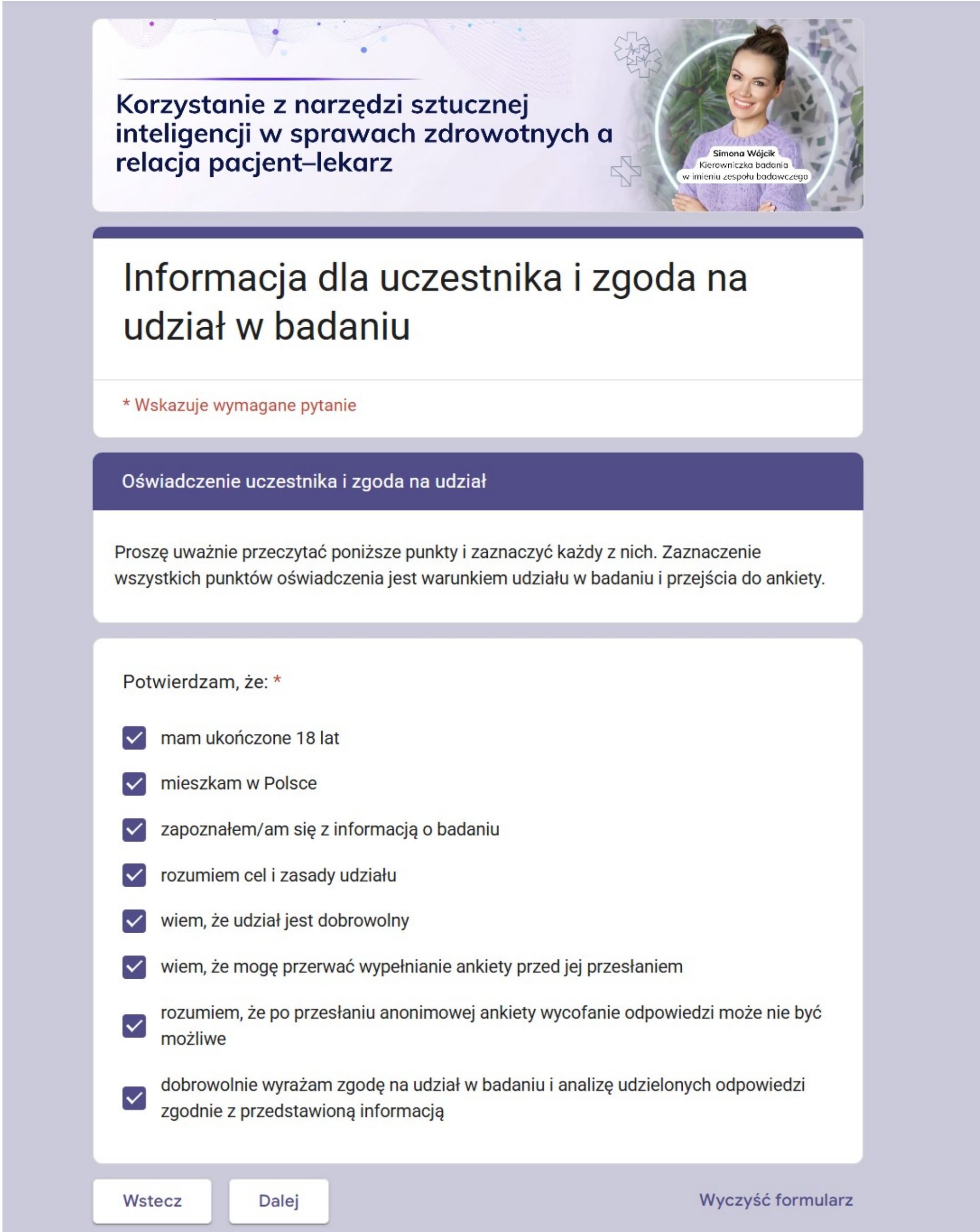

Figure I6. Required eligibility and participant-declaration checklist.

The mandatory declarations confirm age ≥18 years, residence in Poland, reading and understanding the information, voluntariness, the right to discontinue before submission, awareness that withdrawal after anonymous submission may be impossible, and consent to participation and analysis of the responses.

SUPPLEMENTARY FILE J

Questionnaire development and two-stage pretesting

Confirmed histord account for manuscript 1933451

Purpose: document the expert review, participant selection, two-stage pretest procedure, feedback domains, and resulting questionnaire refinements.

1. Expert review

Reviewer	Expertise	Primary review domains	Interpretive boundary
**Expert 1**	Medical communication	Clarity and neutrality of consultation, medication, diagnostic-testing, health-action, and physician-communication items; avoidance of wording implying clinical appropriateness.	Expert review supported face validity and clarity; it was not a formal content-validity-index exercise.
**Expert 2**	Digital health	Logical sequence, routing, cognitive burden, response-option coverage, wording neutrality, and alignment with intended analyses.	The review did not constitute formal survey-methodology certification or psychometric validation.

2. Two-stage pretest

Stage	N	Recruitment	Mode	Procedure	Primary purpose	Outcome
**Stage 1: concurrent cognitive pretesting**	15	Purposive recruitment through acquaintances of acquaintances and unpaid local community groups.	Online Microsoft Teams session.	Participants completed the draft while explaining interpretation and response selection; standardized probes addressed ambiguity, missing options, repetition, judgment, and navigation.	Identify comprehension, response-process, option-coverage, and routing problems.	Iterative refinement of wording, response-option coverage, and routing.
**Stage 2: independent usability pilot**	15	Separate purposively selected group recruited through the same low-cost channels.	Independent online completion followed by structured feedback.	Participants completed the revised questionnaire without assistance, then reported unclear items, response-selection difficulty, missing options, repetition/judgment, and completion time.	Check whether the revised questionnaire functioned without researcher explanation.	Estimated unaided completion time approximately 10 minutes; final usability review.

3. Participant-selection characteristics

- 30 adults, aged 18–72 years, not included in the main N=1,044 sample.
- Purposive maximum-variation selection across age, education, place of residence, digital competence, healthcare use, and previous generative AI experience.
- No compensation or other incentives.
- Participants were informed that the pretest session would last approximately 15–25 minutes.

4. Refinement informed by open-ended feedback

Issue/feedback source	Themes noted	Action in final questionnaire	Caution
**Open-ended comments concerning why a respondent might not inform a physician about prior generative AI use.**	Perceived lack of clinical relevance; fear of a negative physician reaction; insufficient consultation time; physician did not ask; reluctance to undermine physician authority.	Converted into predefined multiple-response Q14 options, with an “other” option retained.	The categories were informed by pretest feedback but do not form a validated barriers scale and should not be interpreted as causal determinants.

5. Methods text

The Polish-language questionnaire was developed by the authors and reviewed before fieldwork by two experts: one specializing in medical communication and one specializing in digital health. The medical-communication expert assessed the clarity and neutrality of items concerning medical consultations, medication use, diagnostic testing, health-related actions, and communication with physicians, including whether any wording could imply that a particular action was clinically appropriate or inappropriate. The digital-health expert reviewed the logical sequence and routing of the questionnaire, cognitive burden, response-option coverage, wording neutrality, and alignment between the items and the intended analyses.

The revised draft underwent a two-stage pretest with 30 adults who were not included in the main study sample. Participants were recruited purposively through acquaintances of acquaintances and unpaid local community groups to maximize variation in age, education, place of residence, digital competence, healthcare use, and previous experience with generative AI. Participants ranged from 18 to 72 years of age, received no compensation, and were informed that participation would take approximately 15–25 minutes. In the first stage, 15 participants completed the questionnaire during Microsoft Teams sessions using concurrent verbal probing. They were asked to explain how they understood selected questions, how they chose their responses, whether an applicable answer option was missing, and whether any item appeared repetitive, judgmental, or difficult to navigate. Feedback was reviewed iteratively and used to refine wording, response-option coverage, and routing. In the second stage, a separate group of 15 participants completed the revised online questionnaire independently and subsequently provided structured feedback on unclear items, difficulty selecting an answer, missing response options, perceived repetition or judgment, and completion time. The second stage supported an estimated unaided questionnaire completion time of approximately 10 minutes.

Some open-ended comments concerning reasons for not informing a physician about previous generative AI use informed the predefined response categories in Q14. These included not considering the information clinically relevant, fear of a negative physician reaction, insufficient consultation time, the physician not asking about AI use, and reluctance to undermine the physician's authority; an 'other' option was retained. The expert review and two-stage pretest were intended to improve face validity, comprehensibility, response-option coverage, routing, and usability. They did not constitute formal psychometric validation.

6. Response to the reviewer

We agree that the original description of questionnaire development was insufficiently reproducible. We have expanded the Methods to specify that the draft was reviewed by one expert in medical communication and one expert in digital health. We now describe their respective review domains, including item clarity and neutrality, clinical wording, routing, cognitive burden, response-option coverage, and alignment with the intended analyses.

We have also clarified that pretesting used a two-stage design involving 30 adults who were not included in the main sample. Participants were purposively recruited through acquaintances of acquaintances and unpaid local community groups to maximize variation in age, education, place of residence, digital competence, healthcare use, and previous AI experience. The first 15 participants completed the questionnaire during Microsoft Teams sessions with concurrent verbal probing. Following iterative revision, a separate group of 15 participants completed the revised questionnaire independently and provided structured feedback. No incentives were provided. The sessions lasted approximately 15–25 minutes, and the independent stage supported an estimated questionnaire completion time of approximately 10 minutes.

Several open-ended comments from the pretest informed the final Q14 response categories. We now explicitly state that this process was intended to improve face validity and usability and did not constitute formal psychometric validation.

7. Manuscript limitatiti

The questionnaire was author-developed and the pretest was designed to assess comprehension and usability rather than psychometric properties. Although purposive recruitment sought variation in age, education, residence, digital competence, healthcare use, and AI experience, the 30 pretest participants were recruited through extended social networks and local community groups and do not constitute a representative sample. Several constructs were assessed using single items, and the pretest did not eliminate limitations related to recall, social

desirability, forced-choice framing, or the absence of a single verified clinical index episode.

8. statements to avoid

- Formal psychometric validation was performed.
- A Content Validity Index was calculated.
- The pretest established construct validity, reliability, or measurement invariance.
- The pretest eliminated ambiguity in Q4, Q6, Q13, or the forced-comparison framing of Q17.
- The pretest sample was representative of Polish adults.

Supplementary File K

Recruitment strategy and anonymized characteristics of directly contacted Instagram accounts

Study: Generative AI use before medical visits: disclosure-item responses, trust, and care-seeking behaviors in a cross-sectional social-media survey in Poland

K1. Confirmed recruitment procedure

Participants were recruited between 1 March and 15 April 2026 through an Instagram-based, multi-account dissemination strategy. The first author published five recruitment invitations on her professional educational Instagram account on 1, 4, 7, 10, and 13 March 2026. The account focused primarily on artificial intelligence and workplace applications; it was not an institutional LUX MED account, and neither the recruitment materials nor the visible profile biography displayed the LUX MED name, logo, or institutional affiliation. One recruitment post was also published on each of the first author's LinkedIn and Facebook accounts on the first day of data collection; because both generated very limited reach, no further posts, sharing, or reposting were undertaken on these platforms.

To broaden dissemination beyond the first author's AI-oriented follower network, the operators of 15 independently managed Instagram accounts were directly asked to share the survey invitation. The accounts represented diverse, predominantly non-medical and non-technical audience profiles, including lifestyle/fashion, beauty and cosmetics, travel, culinary content, fitness and sport, health and wellbeing, parenting and family life, photography and art, and entertainment/humor/memes. Approximate account sizes ranged from 8,000 to 73,000 followers. Further organic redistribution was encouraged, and the invitation was ultimately shared by 101 unique Instagram accounts, including the 15 directly contacted accounts.

No paid advertising, boosted post, or other paid promotion was used. Neither respondents nor account owners received financial or non-financial compensation. The recruitment materials described the survey as concerning health-related use of generative AI and the patient–physician relationship but did not disclose the study hypotheses, expected associations, or preferred response patterns. The same Google Forms link was used across all dissemination sources; therefore, the number of completed questionnaires attributable to individual accounts could not be reconstructed.

K2. Directly contacted Instagram accounts

The table summarizes all 15 independently managed Instagram accounts that were directly asked by the first author to share the invitation. Account names, handles, links, profile images, and exact follower counts are not reported to protect third-party privacy. Approximate follower counts are presented in broad bands. Account audiences may overlap, so follower counts must not be summed to estimate recruitment reach.

Account ID	Primary content category	Approximate follower band
RA-01	Lifestyle/fashion	20,000–25,000
RA-02	Beauty and cosmetics	40,000–45,000
RA-03	Travel	10,000–15,000
RA-04	Travel	5,000–10,000
RA-05	Travel	25,000–30,000
RA-06	Culinary	70,000–75,000
RA-07	Culinary	10,000–15,000
RA-08	Culinary	25,000–30,000
RA-09	Fitness and sport	10,000–15,000
RA-10	Fitness and sport	30,000–35,000
RA-11	Health and wellbeing	5,000–10,000

RA-12	Parenting and family	10,000–15,000
RA-13	Parenting and family	15,000–20,000
RA-14	Photography and art	25,000–30,000
RA-15	Entertainment, humor, and memes	45,000–50,000

K3. Recruitment features relevant to interpretation

- Instagram was the primary recruitment platform; the first author additionally published one low-reach post on each of Facebook and LinkedIn on the first day of data collection only.
- Five first-author recruitment invitations were posted on 1, 4, 7, 10, and 13 March 2026.
- The invitation was shared by 101 unique Instagram accounts in total.
- No paid advertising, boosted post, or other paid promotion was used.
- No respondent or account owner received compensation.
- The LUX MED name, logo, and institutional affiliation were not visible in the recruitment materials or profile biography.
- The study hypotheses, expected associations, and preferred response patterns were not disclosed.
- A single Google Forms link was used, preventing source-specific attribution of completed questionnaires.

K4. Interpretation and limitations

Recruitment used a non-probability Instagram dissemination strategy and therefore cannot support population-level estimates for Polish adults. Although sharing through 101 unique accounts and 15 directly approached accounts broadened exposure beyond the first author’s immediate follower network, the recruitment audience remained shaped by Instagram use, platform algorithms, self-selection, overlap between account audiences, and the willingness of account operators to redistribute the invitation. Because one common survey link was used, source-specific recruitment yields and clustering by recruitment account could not be assessed. The first author’s profile focused on AI-related professional content, which may have increased participation among digitally engaged or AI-interested individuals, even though the LUX MED affiliation, study hypotheses, and expected findings were not displayed in the recruitment materials.

K5. Suggested manuscript wording

Participants were recruited between 1 March and 15 April 2026 through an Instagram-based, multi-account dissemination strategy. The first author published five recruitment invitations on her professional educational Instagram account on 1, 4, 7, 10, and 13 March 2026. The account focused primarily on artificial intelligence and workplace applications; it was not an institutional LUX MED account, and neither the recruitment materials nor the visible profile biography displayed the LUX MED name, logo, or institutional affiliation. One recruitment post was also published on each of the first author's LinkedIn and Facebook accounts on the first day of data collection; because both generated very limited reach, no further posts, sharing, or reposting were undertaken on these platforms.

To broaden dissemination beyond the first author’s AI-oriented follower network, the operators of 15 independently managed Instagram accounts were directly asked to share the survey invitation. The accounts represented diverse, predominantly non-medical and non-technical audience profiles, including lifestyle/fashion, beauty and cosmetics, travel, culinary content, fitness and sport, health and wellbeing, parenting and family life, photography and art, and entertainment/humor/memes. Approximate account sizes ranged from 8,000 to 73,000 followers. Further organic redistribution was encouraged, and the invitation was ultimately shared by 101 unique Instagram accounts, including the 15 directly contacted accounts.

No paid advertising, boosted post, or other paid promotion was used. Neither respondents nor account owners received financial or non-financial compensation. The recruitment materials described the survey as concerning

health-related use of generative AI and the patient–physician relationship but did not disclose the study hypotheses, expected associations, or preferred response patterns. The same Google Forms link was used across all dissemination sources; therefore, the number of completed questionnaires attributable to individual accounts could not be reconstructed.

K6. Response provided to the reviewer

Thank you for requesting a fuller description of the recruitment audience. We agree that the original Methods section inaccurately summarized the recruitment channels and did not sufficiently describe the dissemination network. We have corrected the Methods to state that recruitment was conducted exclusively through Instagram. The first author published five invitations on 1, 4, 7, 10, and 13 March 2026 and directly asked 15 independently managed Instagram accounts to share the invitation. These accounts represented diverse, predominantly non-medical themes and ranged from approximately 8,000 to 73,000 followers. In total, 101 unique Instagram accounts shared the invitation organically. No paid advertising or promotion was used, and neither respondents nor account owners received compensation. The recruitment post and the first author’s visible profile biography did not display a LUX MED affiliation, and the recruitment materials did not disclose the study hypotheses or expected findings. We have also added an anonymized account-characteristics table and expanded the limitations concerning Instagram-based convenience sampling, audience overlap, platform algorithms, self-selection, and the inability to attribute completed questionnaires to individual recruitment accounts.

Supplementary File L

Structural response-quality audit and internal bootstrap validation

Manuscript 1933451 | N=1,044

Purpose. This supplementary analysis addresses the unusual response patterns noted during peer review and provides model-calibration and internal-validation evidence beyond the Hosmer-Lemeshow test. It is a structural and computational audit; it cannot verify respondent identity or exclude automated participation because timestamps, IP addresses, device/user-agent metadata and source-account identifiers were unavailable.

1. Audit objectives

- Verify routing, displayed-item completeness, response-vector uniqueness, exact duplicates and route-aware near duplicates.
- Examine whether the extreme Q17 distribution and the age-by-Q15 association were internally coherent, deterministic, sequentially clustered, or accompanied by broad straightlining.
- Quantify the concentration of Q14 multiple-response patterns without treating repeated option combinations as duplicate respondents.
- Assess the apparent calibration of the primary Q6 and Q11 logistic models and estimate optimism-corrected discrimination, Brier score, Nagelkerke R^2, calibration intercept and calibration slope using nonparametric bootstrap internal validation.

2. Methods

2.1 Structural response-quality audit

Routing and completeness. The complete de-identified export was checked against the programmed Q1, Q4, Q11 and Q13 display rules. For routed multiple-response items, the audit also verified that at least one option was selected whenever the item was displayed.

Duplicate screening. Exact duplicates were defined as identical values across the complete response vector, including structural N/D values. A route-aware near-duplicate scan compared all record pairs and required at least 15 jointly displayed fields and at least 95% identity; exact matches were excluded from the near-duplicate definition.

Extreme-pattern audit. Age-by-Q15 and Q15-by-Q17 contingency tables were evaluated using Cramér's V, entropy, conditional entropy, mutual information and modal classification accuracy. These measures distinguish a strong association from deterministic separation. Sequence checks used the numeric record order only as an export-order proxy, not as a timestamp. Lag-1 correlations and adjacent-category match rates were compared with 5,000 random permutations. Broad straightlining was defined as selecting the same numeric response across Q8, Q9, Q15, Q20 and Q24 among respondents for whom all five items were displayed.

2.2 Internal validation of the logistic models

Model specification. The Q6 and Q11 primary definite-response models were reconstructed using the same 12 predictor parameters as the revised manuscript: gender, five age-group indicators, AI trust, AI-use frequency, digital competency, healthcare-utilization frequency and two education indicators.

Calibration and bootstrap validation. Apparent calibration intercept and slope were obtained by regressing the observed outcome on the original model linear predictor. Their in-sample values are expected to be approximately 0 and 1 by construction and are not treated as evidence of external calibration. Nonparametric bootstrap validation used 1,000 valid resamples for each model. In each resample, the full model was refitted and evaluated both in the bootstrap sample and in the original sample. Mean optimism was subtracted from the apparent AUC, Nagelkerke R^2 and calibration slope, and added to the apparent Brier score. Calibration intercept was corrected using the same train-versus-test optimism framework. Grouped calibration plots use ten rank-based groups, observed proportions with 95% Wilson intervals, the ideal line and the bootstrap-corrected calibration line.

Reproducibility. The final run was executed on Ubuntu 22.04 with Python 3.10.12, NumPy 1.26.4, pandas 2.3.3, SciPy 1.15.3, statsmodels 0.14.6, scikit-learn 1.7.2, Matplotlib 3.10.9, and openpyxl 3.1.5. Separate random seeds were used for Q6 (20260802) and Q11 (20260803). The primary analysis script, separately written verification script, aggregate outputs, environment file, and run-level bootstrap results are deposited in the OSF repository. The individual-level dataset is not publicly deposited.

3. Structural data-quality results

Overall interpretation: the audit found no routing violations, no route-aware near-duplicate pairs and no broad sequential clustering or straightlining. One exact matching full-vector pair was present on the short Q1 non-user route. The extreme Q17 and age-by-Q15 patterns were confirmed, but the age-by-Q15 table retained substantial within-age variation and was not deterministic.

Audit item	Result	Interpretation
Routing violations	0.0	None identified
Unique full response vectors	1043.0	of 1044 records
Exact matching full-vector groups	1.0	1 extra record
Near-duplicate pairs	0.0	At least 15 jointly displayed fields and at least 95% identity; exact matches excluded
Q1=Yes unique vectors	880.0	of 880
Q4=Yes unique vectors	519.0	of 519
Q17 pro-AI responses	0.0	95% Wilson upper bound 0.37%
Age×Q15 Cramer's V	0.45961569369491695	23/30 cells non-zero; modal accuracy 55.8%
Age×Q15 residual entropy	1.4218700743402786	bits; association is strong but not deterministic
Q15×Q17 Spearman rho	0.6531490483140593	graded shift without any pro-AI selection
Five-item straightlining among Q4=Yes	1.0	of 519 (0.19%)
Q14 distinct patterns among Q4=Yes and Q13=No	34.0	top 3 account for 48.6%
Q14 distinct patterns among all Q13=No	37.0	top 3 account for 48.6%
Record-order lag-1 correlation: age	0.030599851167451838	no material sequential clustering
Record-order lag-1 correlation: Q15	0.0031668624695821317	no material sequential clustering
Record-order lag-1 correlation: Q17	-0.05131857304652488	no material sequential clustering

Table L1. Summary of the structural response-quality audit.

Duplicates and uniqueness. Of 1,044 records, 1043 complete response vectors were unique. One exact matching pair was identified, representing one extra record, and no near-duplicate pair met the prespecified route-aware

threshold. All 880 Q1=Yes response vectors and all 519 Q4=Yes response vectors were unique. The exact pair occurred on the short non-user route and therefore did not enter either primary logistic model.

Q17 boundary pattern. No respondent selected either pro-AI option on Q17 (0/1044); the 95% Wilson upper bound was 0.37%. This confirms the observed boundary but does not establish whether it arose from true preference, fixed option order, social desirability, forced comparison framing or another response-process effect.

Age-by-Q15 association. The association was strong (Cramér's V=0.460), but not deterministic: 23 of 30 cells were non-zero, conditional Q15 entropy within age groups remained 1.422 bits, and using only age-group modal responses classified Q15 exactly in 55.8% of respondents.

Coherent related gradients. Age was also negatively associated with digital competency (Spearman ρ=-0.614) and, among AI users, AI-use frequency (ρ=-0.554). Q15 correlated with digital competency (ρ=0.475) and with Q17 (ρ=0.653). This coherence is compatible with a real digital-experience gradient, although it does not prove respondent authenticity.

Sequence and straightlining checks. Lag-1 correlations in export order were 0.031 for age, 0.003 for Q15 and -0.051 for Q17. Adjacent-match rates were within permutation reference intervals. Record-quartile associations were statistically detectable but small (Cramér's V=0.084 for Q15 and 0.093 for age), consistent with minor recruitment-wave variation rather than obvious response batching. Only 1/519 respondents (0.19%) selected the same value across all five displayed ordinal items.

Q14 response combinations. Among the 428 pre-consultation AI users selecting No on Q13, 34 distinct Q14 option patterns occurred; the largest single pattern represented 24.8% and the three most common patterns together 48.6%. Repetition of multiple-choice combinations did not translate into repeated complete response vectors.

3.1 Age-by-Q15 distribution

Age group	Q15=1	Q15=2	Q15=3	Q15=4	Q15=5
18–24	0	2	19	85	35
25–34	0	0	32	169	95
35–44	4	61	149	50	1
45–54	7	74	83	8	0
55–64	13	71	36	2	0
65+	16	26	6	0	0

Table L2. Counts for AI trust (Q15) by age group.

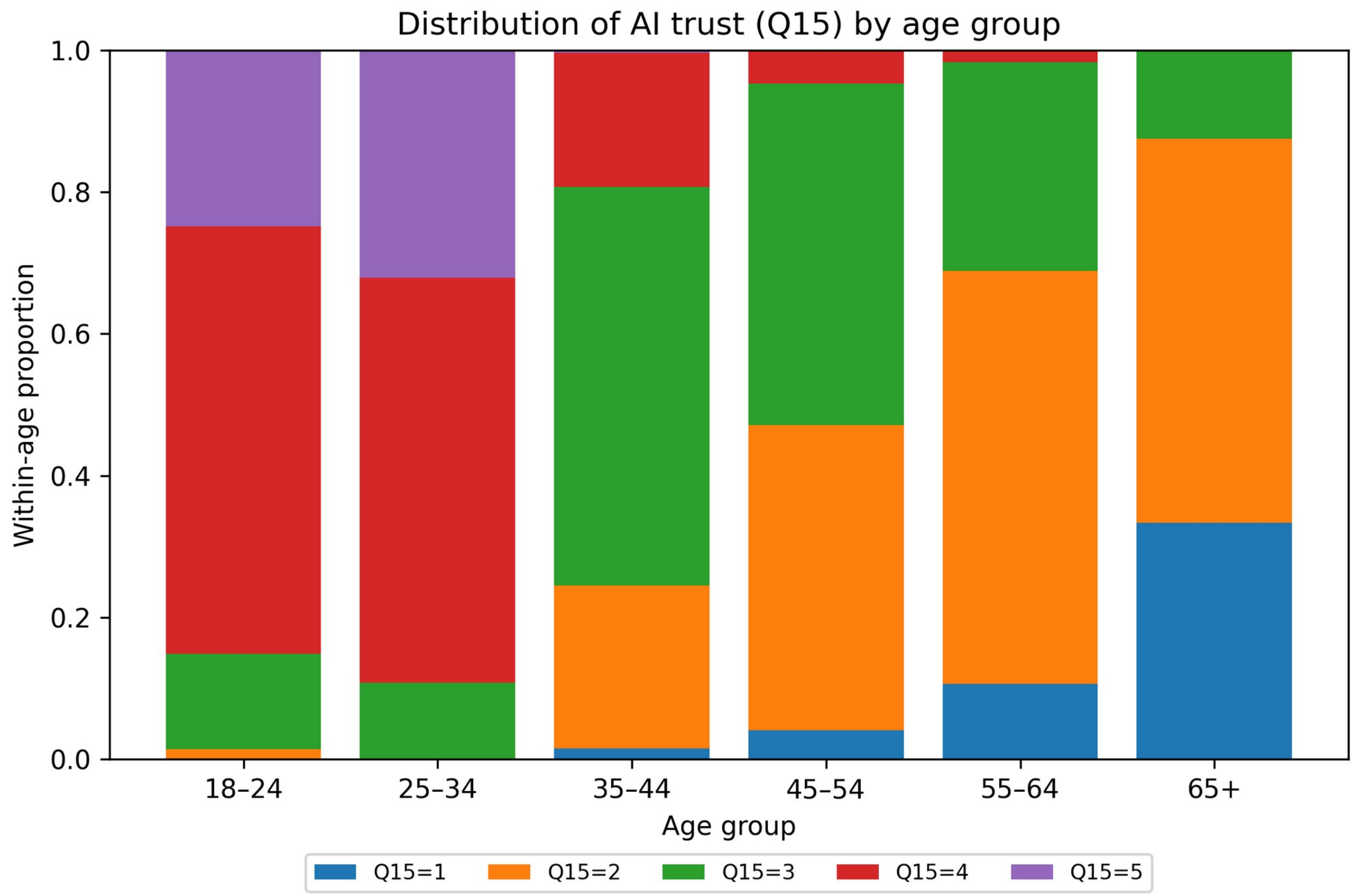

Figure L1. Within-age distribution of Q15 responses. The relationship is strong but retains multiple response categories within each age group.

3.2 Q15-by-Q17 distribution

Q15	Definitely physician	Rather physician	Difficult to say	Rather AI	Definitely AI
1	39	1	0	0	0
2	213	21	0	0	0
3	222	103	0	0	0
4	71	214	29	0	0
5	7	90	34	0	0

Table L3. Counts for forced physician-versus-AI preference (Q17) by Q15 trust score.

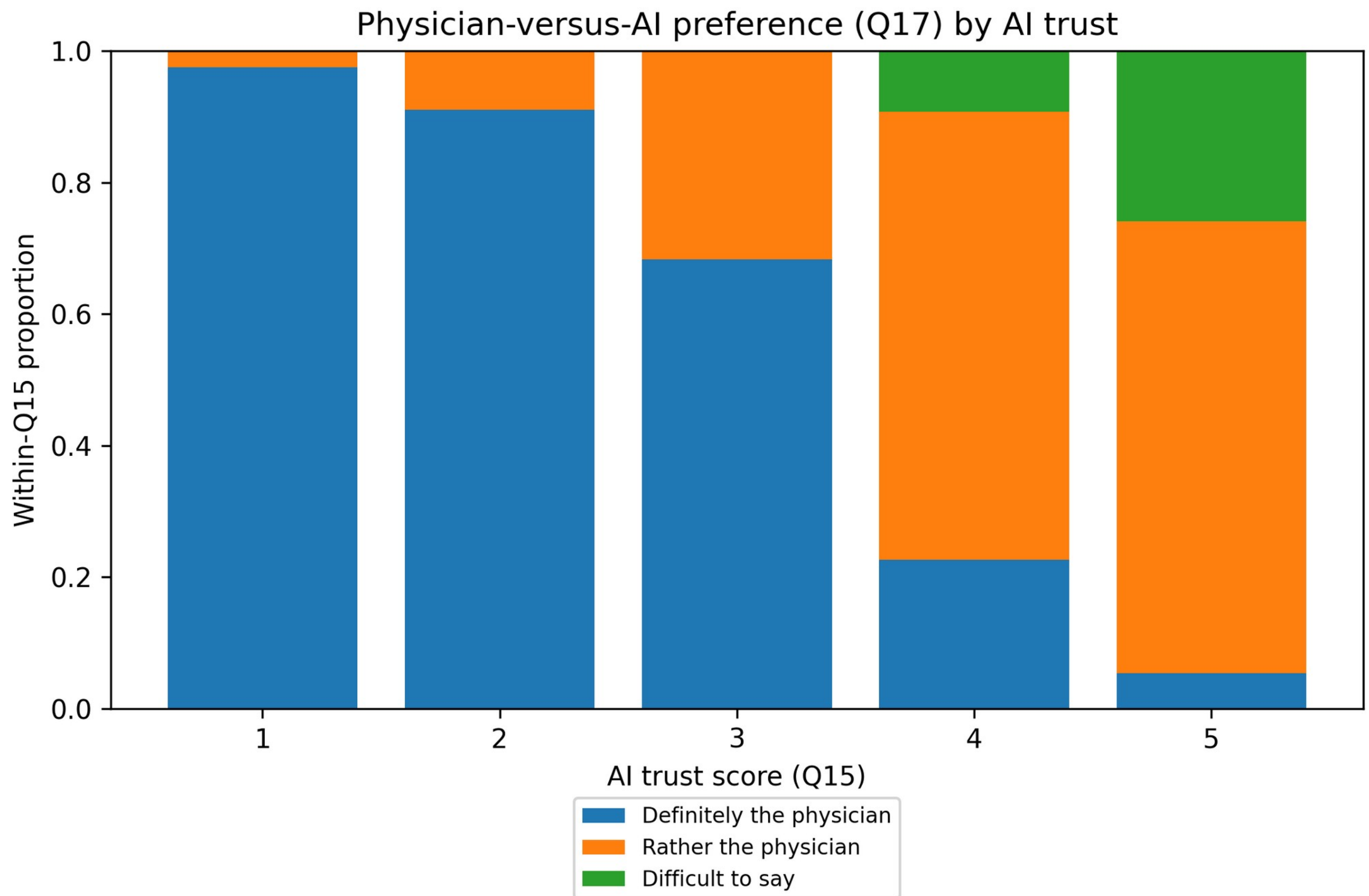

Figure L2. Q17 response distribution by Q15. Higher AI trust shifted responses from definite to qualified physician preference and to uncertainty, without crossing into explicit AI preference.

4. Internal validation of the primary models

The Hosmer-Lemeshow p-values are not presented as proof of adequate calibration. Apparent intercepts near 0 and slopes near 1 are expected in the development sample. The clinically relevant evidence is the bootstrap optimism correction: Q6 showed more overfitting and less stable resampling than Q11.

Metric	Q6 apparent	Q6 optimism-corrected	Q11 apparent	Q11 optimism-corrected
AUC	0.720	0.684	0.687	0.665
Brier score	0.195	0.208	0.223	0.231
Nagelkerke R^2	0.183	0.096	0.139	0.099
Calibration intercept	-0.000	-0.114	0.000	-0.009
Calibration slope	1.000	0.793	1.000	0.871

Table L4. Apparent and bootstrap optimism-corrected model performance (1,000 valid resamples per model).

Q6 model. The apparent AUC was 0.720 and decreased to 0.684 after optimism correction. Nagelkerke R^2 decreased from 0.183 to 0.096. The corrected calibration slope was 0.793, indicating moderate overfitting, and the corrected intercept was -0.114, consistent with slight average overprediction. Obtaining 1,000 valid resamples required 1,122 sampling attempts; 122 refits were non-convergent or failed numerically.

Q11 model. The apparent AUC was 0.687 and the optimism-corrected AUC was 0.665. Nagelkerke R^2 decreased from 0.139 to 0.099. The corrected calibration slope was 0.871 and the corrected intercept -0.009. All 1,000 resamples produced valid model fits.

Interpretation. Both models retain only modest internally validated discrimination and explained variation. The Q6 model is more sensitive to overfitting and resampling instability. These findings do not alter the reported odds-ratio estimates in the development sample, but they require the performance claims to remain descriptive and exploratory. Neither model has undergone external validation.

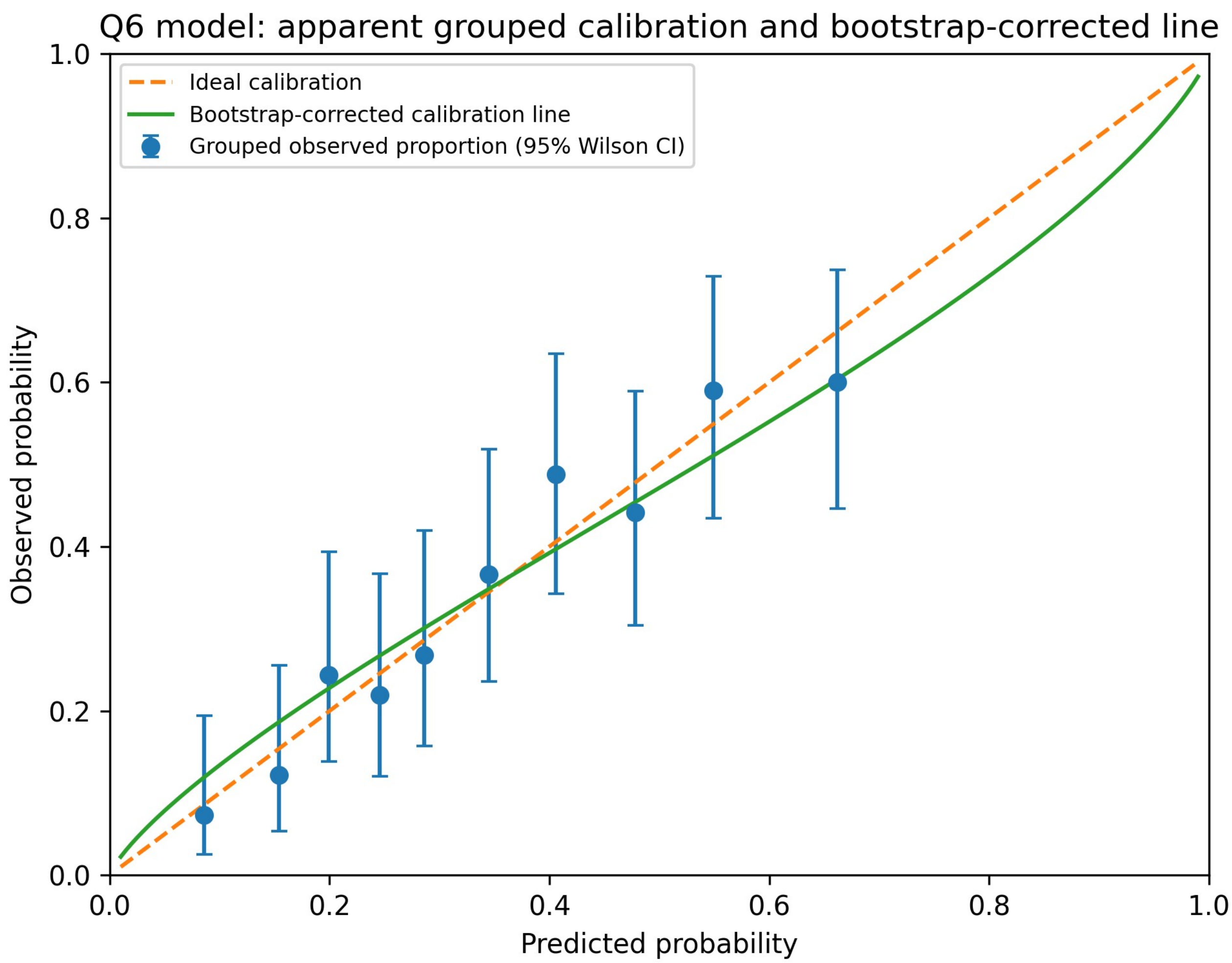

Figure L3. Q6 grouped apparent calibration with 95% Wilson intervals and bootstrap-corrected calibration line.

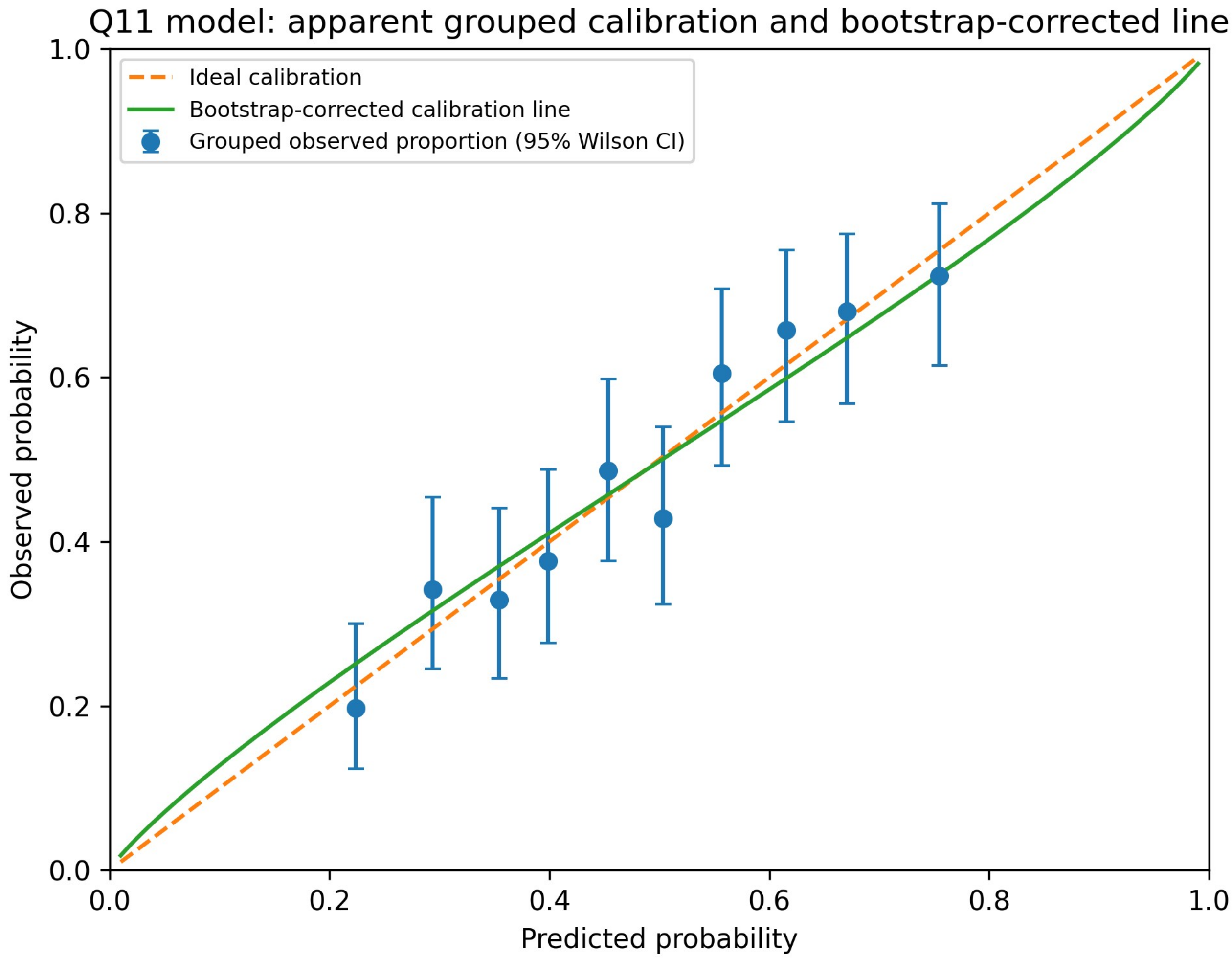

Figure L4. Q11 grouped apparent calibration with 95% Wilson intervals and bootstrap-corrected calibration line.

5. Manuscript-ready reporting text

Methods addition

To examine unusual response patterns, we conducted a structural response-quality audit of the complete de-identified export. The audit verified bidirectional routing and displayed-item completeness, counted exact full-vector matches, and performed a route-aware near-duplicate scan requiring at least 15 jointly displayed fields and at least 95% identity. Age-by-Q15 and Q15-by-Q17 structures were quantified using contingency tables, Cramér's V, entropy and modal classification accuracy. We also assessed broad straightlining across five ordinal items and tested sequential clustering in export order using lag-1 correlations and permutation-based adjacent-match rates. Because timestamps, IP addresses, device/user-agent metadata and source-account identifiers were unavailable, these analyses could not establish respondent identity or exclude automated or multiple participation.

Model performance was internally validated using 1,000 nonparametric bootstrap resamples with the full primary model specification refitted in each resample. We estimated optimism-corrected AUC, Brier score, Nagelkerke R^2, calibration intercept and calibration slope. Apparent calibration intercept and slope were not interpreted as independent evidence because values near 0 and 1 are expected in the development sample. Grouped calibration plots show observed outcome proportions with 95% Wilson confidence intervals and bootstrap-corrected calibration lines.

Results addition

No routing violation was detected. There were 1,043 unique complete response vectors among 1,044 records, with one exact matching pair on the short Q1 non-user route and no route-aware near-duplicate pair. All 880 Q1=Yes vectors and all 519 Q4=Yes vectors were unique. Only one of 519 respondents with all five relevant ordinal items displayed selected the same response across Q8, Q9, Q15, Q20 and Q24. No pro-AI Q17 response was observed (0/1,044; 95% Wilson upper bound 0.37%). The age-by-Q15 association was strong (Cramér's V=0.460) but not deterministic: 23 of 30 cells were non-zero, conditional Q15 entropy within age groups was 1.422 bits, and age-group modal values classified Q15 exactly in 55.8% of respondents. Export-order lag-1 correlations were close to zero and adjacent-category match rates did not exceed permutation reference distributions.

Bootstrap internal validation indicated performance optimism in both logistic models. For Q6, the AUC decreased from 0.720 apparent to 0.684 optimism-corrected, Nagelkerke R^2 from 0.183 to 0.096, and the corrected calibration slope was 0.793 with intercept -0.114. For Q11, the corresponding values were AUC 0.687 to 0.665, Nagelkerke R^2 0.139 to 0.099, calibration slope 0.871 and intercept -0.009. These results support describing model discrimination and calibration as modest and internally optimism-sensitive rather than validated.

Response-to-reviewer wording

We agree that the extreme Q17 distribution, the strong age-by-trust gradient and the absence of platform-level paradata required additional scrutiny. We therefore added a structural data-quality audit and internal bootstrap validation as Supplementary File L. The audit identified no routing violations, no route-aware near-duplicate pairs, no broad straightlining and no material sequential clustering in export order. One exact matching pair occurred on the short non-user route; all Q1=Yes and Q4=Yes full response vectors were unique. The age-by-Q15 association was strong but retained substantial within-age variation and was not deterministic. We explicitly retain the limitation that these checks cannot exclude automated or multiple participation because timestamps, IP addresses, device information and source-account identifiers were unavailable. We also replaced reliance on Hosmer-Lemeshow p-values with calibration intercepts, slopes, calibration plots and 1,000-resample bootstrap internal validation. The internally corrected performance was lower than the apparent performance, particularly for Q6, and the revised manuscript now describes both models as exploratory, modest and not externally validated.

6. Interpretation boundaries

- The audit provides evidence against widespread exact duplication, route violations, broad straightlining and obvious export-order batching. It does not prove that every record corresponds to a distinct human respondent.
- The strong age-by-Q15 association is confirmed but is not a near-complete separation: substantial response entropy remains within age groups. Its magnitude may still reflect recruitment, wording or selection effects.
- The zero pro-AI Q17 count is verified. It remains a measurement boundary affected by forced comparison, fixed option order and possible social desirability, and should not be generalized as population-level rejection of AI.
- Bootstrap validation is internal. It estimates optimism under repeated sampling from the same empirical population and does not establish transportability to another sample or clinical setting.
- The corrected Q6 calibration slope below 0.80 and the failed bootstrap refits indicate that Q6 model performance should not be presented as stable or validated. The model remains suitable only for exploratory association reporting with full uncertainty.

7. Accompanying reproducibility files

- data_quality_and_internal_validation.py — separate executable analysis implementation
- Supplementary_File_L_summary.json — machine-readable complete summary
- Table_L1_data_quality_summary.csv — aggregate response-quality results
- Table_L2_age_by_Q15_counts.csv and row proportions
- Table_L3_Q15_by_Q17_counts.csv and row proportions
- Table_L4_internal_validation_summary.csv — apparent and optimism-corrected metrics
- Table_L5_Q6_calibration_groups.csv and Table_L6_Q11_calibration_groups.csv
- bootstrap_validation_replicates.csv — 2,000 aggregate bootstrap result rows
- Figures L1–L4 — age/Q15, Q15/Q17 and calibration plots

This supplementary file reports a new analysis and does not modify the original response dataset.

Supplementary File N

Regression stability, penalization, sparse cells, functional form, and influence diagnostics

Purpose: to test whether the principal Q6 and Q11 odds ratios for male gender and AI trust depend materially on maximum-likelihood estimation, sparse age cells, influential observations, the coding of age, or resampling variability.

Methods

The revised definite-response logistic models were re-estimated using standard maximum likelihood, Firth bias-reduced logistic regression, and cross-validated ridge logistic regression. Nonparametric bootstrap resampling (1,000 replicates) assessed the distribution of the main odds ratios. Influence screening used Cook's distance >4/n and leverage >2p/n from an equivalent binomial generalized linear model; a sensitivity model removed the union of observations meeting either threshold. Age was alternatively represented as the six original categories, a pooled 55+ category, a single ordered term, and a restricted cubic spline. These analyses assess numerical and specification stability and do not constitute external validation.

Main coefficient stability

Outcome	Method	Term	OR	95% CI	p
Q6	Standard MLE	Male gender	2.98	1.83–4.86	1.17e-05
Q6	Standard MLE	AI trust	1.90	1.27–2.83	0.00168
Q6	Firth	Male gender	2.87	1.77–4.66	1.99e-05
Q6	Firth	AI trust	1.85	1.25–2.75	0.00227
Q6	Ridge CV C=0.1	Male gender	1.83		
Q6	Ridge CV C=0.1	AI trust	1.30		
Q6	Remove influential union	Male gender	3.16	1.87–5.33	1.64e-05
Q6	Remove influential union	AI trust	1.98	1.28–3.06	0.00225
Q11	Standard MLE	Male gender	2.40	1.72–3.35	2.21e-07
Q11	Standard MLE	AI trust	1.56	1.22–2.01	0.000495
Q11	Firth	Male gender	2.36	1.70–3.29	3.42e-07
Q11	Firth	AI trust	1.55	1.21–1.99	0.000615
Q11	Ridge CV C=0.0562341	Male gender	1.71		
Q11	Ridge CV C=0.0562341	AI trust	1.37		
Q11	Remove influential union	Male gender	2.46	1.74–3.47	3.03e-07
Q11	Remove influential union	AI trust	1.57	1.21–2.03	0.000709

Both principal coefficients remained positive under every method. Firth estimates were close to the standard maximum-likelihood estimates. Ridge penalization produced smaller coefficients, as expected from shrinkage, but did not reverse either association. Removing all observations meeting at least one influence threshold also did not materially change the main estimates.

Bootstrap and numerical stability

Outcome	Metric	Successful replicates	Median	2.5th–97.5th percentile
Q6	Male OR	976	3.031	1.794–5.258
Q6	AI-trust OR	976	1.924	1.271–2.988

Outcome	Metric	Successful replicates	Median	2.5th–97.5th percentile
Q6	Apparent AUC in resampled data	976	0.739	0.690–0.787
Q11	Male OR	1000	2.459	1.771–3.366
Q11	AI-trust OR	1000	1.582	1.238–2.063
Q11	Apparent AUC in resampled data	1000	0.700	0.663–0.738

The standard model fitted successfully in 976/1,000 Q6 bootstrap samples and all 1,000 Q11 samples. Bootstrap failures in Q6 are evidence of sensitivity to sparse or separated resamples and should be reported rather than silently discarded. The bootstrap AUC values shown here describe model performance in each resampled development dataset; optimism-corrected performance is reported separately in Supplementary File L.

Influence and sparse cells

Outcome	N	Cook >4/n	Leverage >2p/n	Union flagged	Maximum Cook D	Maximum leverage
Q6	409	14	13	25	0.0336	0.153
Q11	759	12	29	33	0.0096	0.059

These thresholds are screening rules, not formal exclusion criteria. The union-removal analysis was deliberately conservative and retained the direction and magnitude of the male-gender and AI-trust associations. For Q6, the 65+ category contained only 15 respondents and 2 events, explaining its very wide confidence interval. The 55–64 category contained 43 respondents and 18 events; its individual unadjusted contrast should not be treated as evidence of an age effect when the global age test is not conventionally significant.

Age functional-form sensitivity

Outcome	Age specification	Male OR	Male 95% CI	AI-trust OR	AI-trust 95% CI	AUC
Q6	Categorical	2.98	1.83–4.86	1.90	1.27–2.83	0.720
Q6	Pooled 55+	2.89	1.78–4.69	1.93	1.29–2.87	0.713
Q6	Ordinal	2.82	1.75–4.55	1.71	1.19–2.46	0.707
Q6	Restricted cubic spline	2.81	1.74–4.54	1.82	1.25–2.65	0.711
Q11	Categorical	2.40	1.72–3.35	1.56	1.22–2.01	0.687
Q11	Pooled 55+	2.41	1.73–3.35	1.56	1.21–2.00	0.687
Q11	Ordinal	2.39	1.72–3.33	1.61	1.28–2.04	0.688
Q11	Restricted cubic spline	2.40	1.72–3.34	1.58	1.25–2.01	0.687

The principal associations were stable across categorical, pooled, ordinal, and spline age specifications. For Q6, model discrimination varied modestly (AUC 0.707–0.720). For Q11, AUC was essentially unchanged (0.687 across specifications).

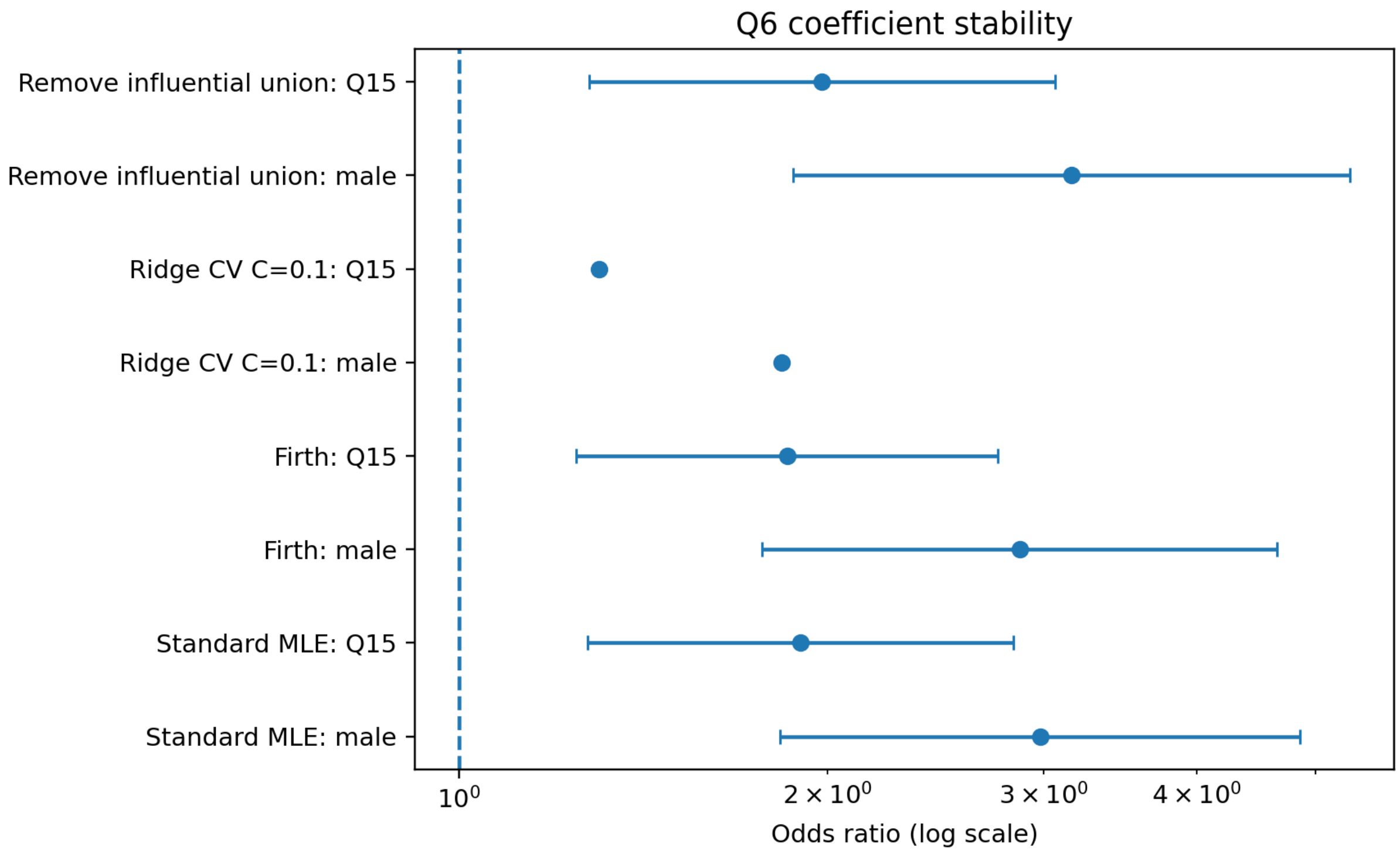

Figure N1. Q6 coefficient stability across estimation and influence-sensitivity approaches. Ridge estimates are penalized point estimates and do not have conventional Wald confidence intervals in this analysis.

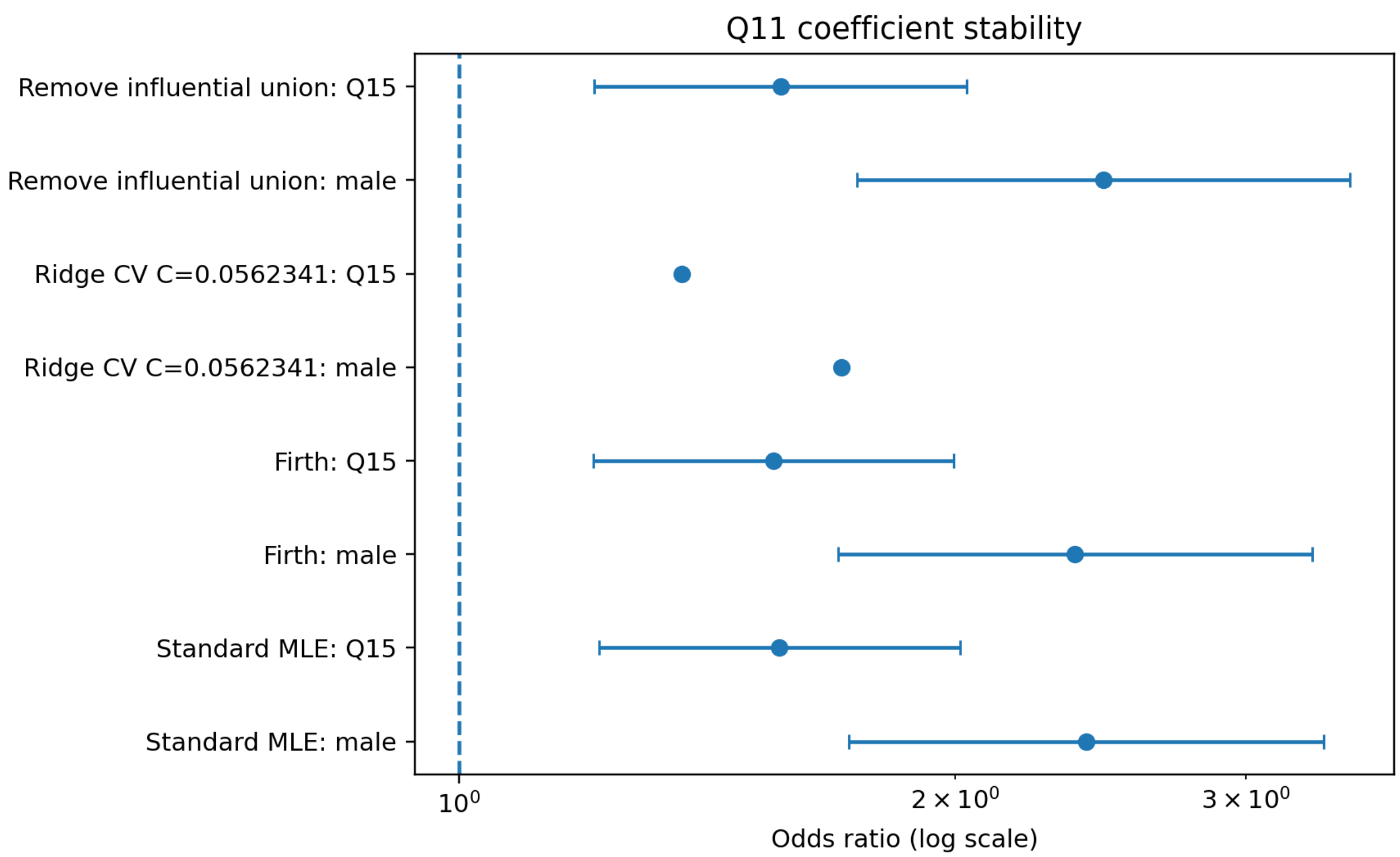

Figure N2. Q11 coefficient stability across estimation and influence-sensitivity approaches.

Conclusion and reporting

The associations of male gender and greater AI trust with Q6 and Q11 are not artifacts of a single maximum-likelihood specification. Their direction persists under Firth correction, ridge shrinkage, removal of flagged observations, alternative age functions, and bootstrap resampling. Q6 is nevertheless less stable than Q11 because of sparse age cells and bootstrap convergence failures. Both models remain exploratory association models and should not be presented as externally validated prediction models.

Methods text

Regression-stability analyses re-estimated the primary definite-response models using Firth bias-reduced logistic regression and cross-validated ridge regression. Nonparametric bootstrap resampling with 1,000 replicates assessed the distributions of the male-gender and AI-trust odds ratios. Influence diagnostics were obtained from an equivalent binomial generalized linear model using Cook's distance and leverage screening thresholds, followed by a sensitivity analysis excluding the union of flagged observations. Age was alternatively represented categorically, with the two oldest groups pooled, as an ordered term, and using a restricted cubic spline. These analyses evaluated numerical and specification stability and did not constitute external validation.

Results text

The direction of the associations for male gender and AI trust was unchanged under Firth correction, ridge shrinkage, removal of flagged observations, and alternative age specifications. Firth estimates were close to the maximum-likelihood estimates. In 1,000 nonparametric bootstrap samples, 976 Q6 models and all 1,000 Q11 models fitted successfully; percentile intervals for the main odds ratios excluded 1. Q6 was more sensitive to resampling and sparse age cells than Q11. These findings support coefficient-direction stability but do not establish external predictive validity.

Supplementary File O

Selection and descriptive standardization sensitivity

Purpose: to show how selected estimates change when the convenience sample is calibrated to available Polish age, gender, and education margins. These are descriptive sensitivity analyses, not representative population estimates.

Methods

Raking weights calibrated the sample to three broad adult age groups derived from end-2024 Statistics Poland counts, a contextual women/men distribution, and a four-category education distribution harmonized from the 2021 Census. Because 50 respondents preferred not to report gender, their observed share was preserved while the remaining target share was divided between women and men. Five scenarios were examined: unweighted, age only, age plus gender, age plus gender plus education, and age plus gender plus education with weights capped at 10 and renormalized. Kish effective sample size and design effect describe weight instability. Percentile intervals were obtained from 1,000 fixed-weight nonparametric bootstrap resamples and therefore condition on the estimated weights.

Weight diagnostics

Scenario	Min	Median	99th percentile	Max	Kish ESS	Design effect
Unweighted	1.00	1.00	1.00	1.00	1044.0	1.00
Age only	0.63	0.63	5.47	5.47	518.4	2.01
Age + gender	0.55	0.74	6.37	6.37	514.9	2.03
Age + gender + education	0.35	0.55	3.69	70.06	112.6	9.27
Age + gender + education (trimmed at 10)	0.40	0.62	4.16	11.29	460.6	2.27

The untrimmed age–gender–education calibration is unstable: the maximum weight is 70.1 and Kish effective sample size falls to approximately 113. This is driven primarily by the severe underrepresentation of respondents with primary education (7/1,044). The trimmed scenario is substantially more stable but no longer exactly reproduces all target margins.

Estimate sensitivity

Outcome	Unweighted	Age only	Age + gender	Age + gender + education	Trimmed at 10
Q1 Yes (full sample)	84.3% (82.0–86.6)	79.5% (75.7–83.2)	79.6% (75.7–83.2)	76.7% (65.7–85.3)	80.8% (76.3–84.6)
Q4 Yes among Q1 Yes	59.0% (55.6–62.2)	57.9% (53.6–62.2)	58.0% (53.8–62.6)	61.0% (54.0–67.2)	59.0% (54.1–63.4)
Q13 No among Q4 Yes	82.5% (79.2–85.5)	81.4% (76.1–86.2)	81.8% (76.6–86.3)	72.2% (56.5–85.6)	79.9% (73.4–85.7)
Q6 Yes among definite Q4 Yes	33.6% (28.8–37.9)	29.2% (24.0–34.7)	30.9% (25.6–36.6)	29.5% (20.5–40.4)	31.8% (25.5–39.1)
Q10 composite Yes among applicable Q1 Yes	66.6% (63.6–70.0)	67.5% (63.3–71.8)	65.5% (60.9–70.0)	65.8% (59.4–72.6)	64.0% (59.2–69.0)
Q11 Yes among definite Q1 Yes	47.6% (44.0–50.9)	44.3% (39.4–49.1)	45.7% (40.8–50.7)	41.5% (36.5–46.4)	42.3% (37.5–46.8)
Q15 mean (full sample)	3.25 (3.18–3.32)	2.80 (2.70–2.91)	2.81 (2.70–2.90)	2.84 (2.68–2.99)	2.94 (2.84–3.04)

Age calibration lowers the estimated prevalence of health-related AI use and the mean trust score, consistent with the strong underrepresentation of older adults. Q13=No remains high under age-only and

age-plus-gender calibration, but the untrimmed education-calibrated estimate is unstable because a handful of respondents with primary education receive very large weights. The trimmed estimate for Q13=No is 79.9%, compared with 82.5% unweighted. Q6 and Q11 estimates also decline modestly after calibration.

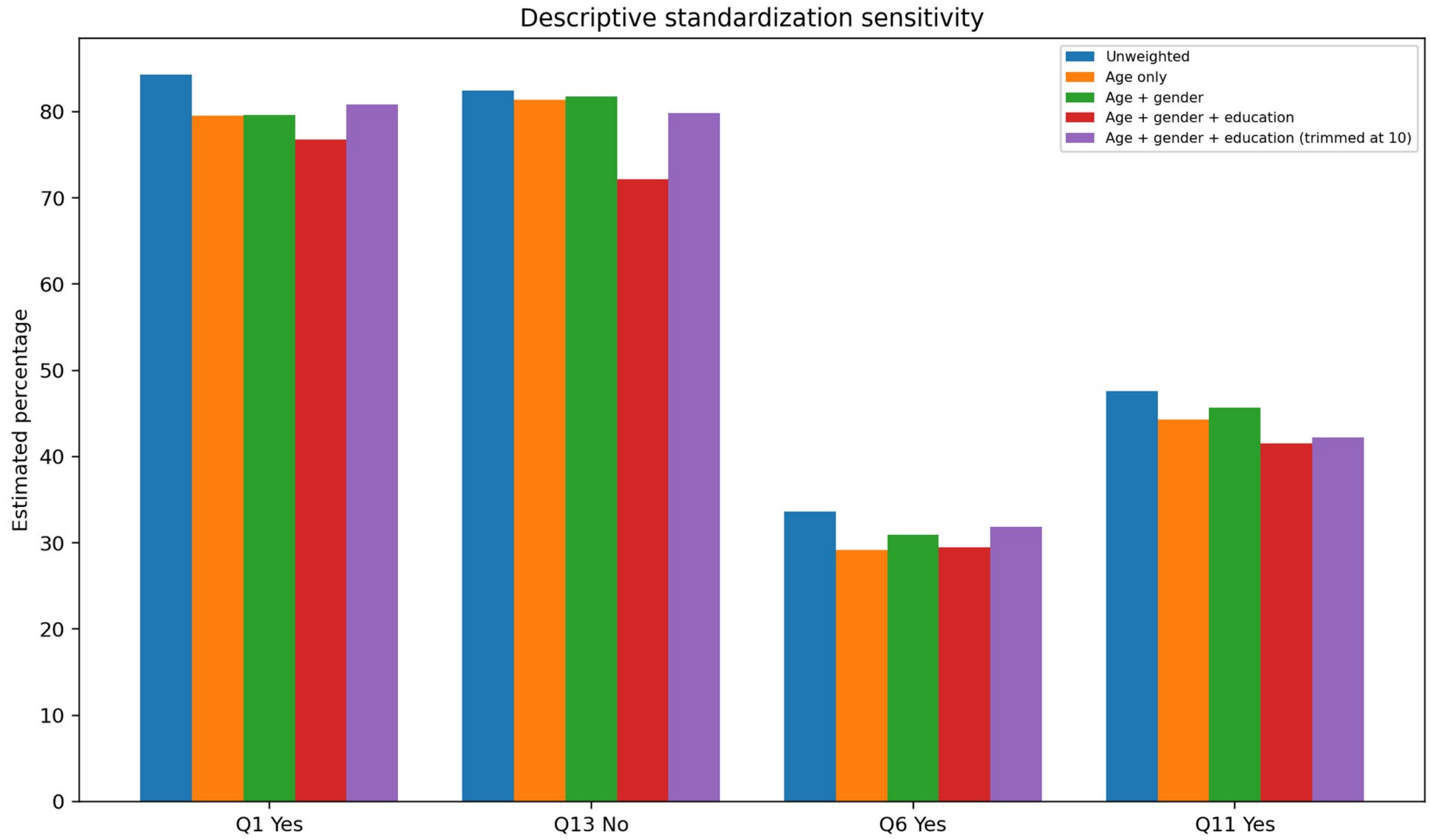

Figure O1. Unweighted and descriptively standardized estimates. The untrimmed education-calibrated scenario is shown for transparency but should not be treated as the preferred estimate because of extreme weights.

Interpretation boundary

Calibration on observed age, gender, and education cannot reconstruct unknown probabilities of exposure to the Instagram invitation, account-level clustering, differential interest in AI and health, digital engagement, or willingness to participate. The weighted values must not be described as nationally representative estimates. Their proper role is to demonstrate that the convenience-sample composition materially influences some magnitudes, especially Q1 and Q15, while the central Q13 pattern remains high in the more stable age-only, age-plus-gender, and trimmed scenarios.

Interpretive limitations

Descriptive calibration to available Polish age, gender, and education margins reduced the estimated prevalence of health-related AI use and mean AI trust, confirming that the sample composition influenced observed magnitudes. However, education calibration produced extreme weights because only seven respondents reported primary education, and even trimmed weights cannot correct unknown selection related to social-media exposure, digital engagement, interest in AI, or willingness to participate. These analyses are therefore reported only as sensitivity analyses and not as population-representative weighted estimates.

Supplementary File P. Complete questionnaire routing specification

Manuscript 1933451. This file gives the full, unambiguous conditional-display logic of the 24-item instrument as implemented in the survey platform and confirmed against the complete data export, so that the survey can be reproduced item-for-item. It complements the flow diagram (Supplementary Figure S1) and the item-response table (Supplementary File D). N/D denotes an item that was structurally not displayed by the routing and is not respondent-level missingness.

Correction note. An earlier version of the questionnaire document instructed Q1 non-users to proceed to Item 18. This was a documentation error; the implemented route sent them to Item 15, as confirmed by the response export (all 164 non-users have N/D on Q2–Q14 and complete responses on Q15–Q24). The specification below reflects the implemented (correct) routing.

Per-item display conditions and branching

Item	Question (abbreviated)	Displayed to	Response options	Branch / skip rule	Analysis base
Q1	Ever used generative AI for health?	All (N=1044)	Yes / No / Don't know–remember	No or Don't-know → skip Q2–Q14, go to Q15	All
Q2	Frequency of health-related AI use	Q1=Yes (880)	4-point ordinal	—	Q1=Yes
Q3	Purposes of AI use (multiple response)	Q1=Yes (880)	10 options + other	—	Q1=Yes
Q4	AI use before a medical visit?	Q1=Yes (880)	Yes / No / Don't remember	No or Don't-remember → skip Q5–Q9, go to Q10	Q1=Yes
Q5	Perceived influence on contacting a physician	Q4=Yes (519)	4 categories	—	Q4=Yes
Q6	Forwent a visit because AI said it was unnecessary	Q4=Yes (519)	Yes / No / Don't remember / Prefer not	—	Q4=Yes (definite Yes/No primary)
Q7	Scheduled a visit sooner because of AI	Q4=Yes (519)	Yes / No / Don't remember	—	Q4=Yes
Q8	Felt better prepared for the visit (1–5)	Q4=Yes (519)	5-point	—	Q4=Yes
Q9	AI increased health anxiety (1–5)	Q4=Yes (519)	5-point	—	Q4=Yes
Q10	Additional testing and/or second opinion	Q1=Yes (880)	tests / 2nd opinion / both / no / N-A	—	Q1=Yes (exclude Not applicable)
Q11	Behavior change without a	Q1=Yes (880)	Yes / No / Don't	Yes → show Q12	Q1=Yes (definite Yes/No

	physician?		remember / Prefer not		primary)
Q12	Types of change (multiple response)	Q11=Yes	7 options + other	—	Q11=Yes
Q13	Told the physician about AI use?	Q1=Yes (880)	Yes / No / Don't remember / N-A	No → show Q14	Q4=Yes (primary disclosure)
Q14	Reasons for not telling (multiple response)	Q13=No	6 options	—	Q13=No
Q15	Trust in AI health information (1–5)	All (1044)	5-point	—	primary Q1=Yes; full-sample secondary
Q16	Questioned physician's diagnosis/recommendations	All (1044)	Yes / No / Don't remember / N-A	—	Q1=Yes (item meaningful only for AI users)
Q17	Trust more: physician vs AI (forced choice)	All (1044)	5-point physician ↔ AI	—	All
Q18	Physicians should ask about AI use?	All (1044)	Yes / No / Don't know	—	All
Q19	Facilities should educate on safe AI use?	All (1044)	Yes / No / Don't know	—	All
Q20	Digital competency (1–5)	All (1044)	5-point	—	covariate
Q21	Age group	All (1044)	6 groups	—	covariate (categorical)
Q22	Gender	All (1044)	Woman/Man/Other/Prefer not	Prefer-not excluded from gender analyses	covariate
Q23	Education	All (1044)	5 levels	—	covariate (categorical)
Q24	Healthcare-utilization frequency (1–5)	All (1044)	5-point	—	covariate; full sample for gender comparison

Branching logic (pseudocode)

```
display Q1
if Q1 in {No, Don't-know/remember}:      # n=164
    do NOT display Q2..Q14               # recorded as N/D
    go to Q15
else:                                     # Q1=Yes, n=880
    display Q2, Q3
    display Q4
    if Q4 == Yes:           display Q5, Q6, Q7, Q8, Q9   # n=519
    else:                   skip Q5..Q9                  # go to Q10
    display Q10
    display Q11
    if Q11 == Yes:          display Q12
    display Q13
    if Q13 == No:           display Q14
```

```
display Q15, Q16, Q17, Q18, Q19, Q20, Q21, Q22, Q23, Q24   # all respondents
```

Analysis-base note: an item's display condition and its analysis base can differ. Q13 was displayed to all Q1=Yes respondents but the primary disclosure analysis uses the Q4=Yes base (pre-consultation users). Q16 was displayed to all respondents but is analyzed among Q1=Yes users, for whom the item is meaningful; no respondent without AI use gave an affirmative response.

Figure S1. Programmed questionnaire routing as configured in the fielded form

Solid arrows: default progression. Dashed orange arrows: configured conditional jumps; items in skipped sections were never displayed and are recorded as “not displayed”.
Section numbers follow the fielded form. Branching configuration is evidenced by form screenshots (Supplementary File I-b) and specified in Supplementary File P.
Counts are observed responses from the analytic sample (N = 1,044).

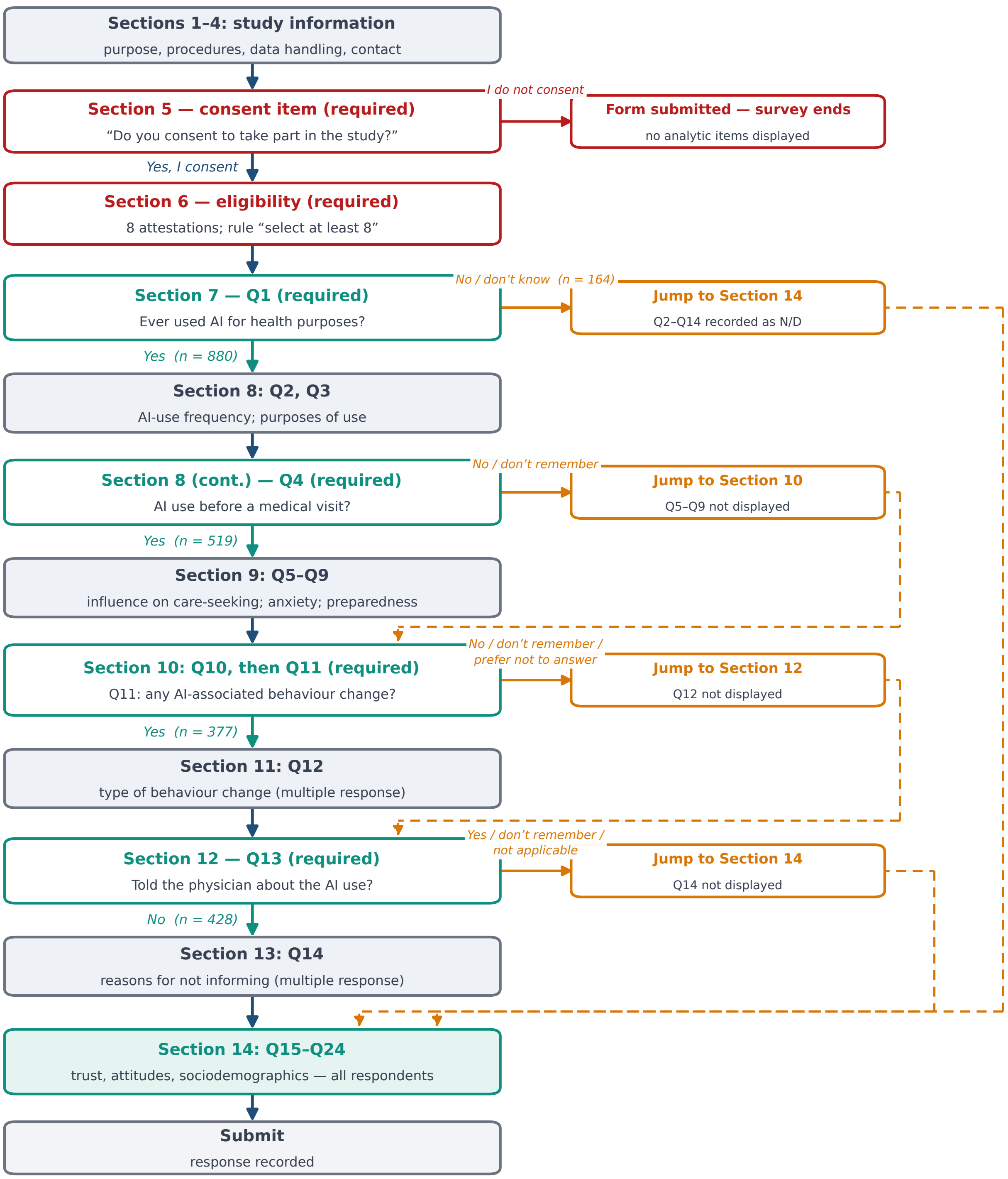

Supplement: Supplementary file 1 [file Datasheet1.pdf]
