# Supplementary material for: Generative AI use before medical visits: disclosure-item responses, trust, and care-seeking behaviors in a cross-sectional social-media survey in Poland
Source: Front Digit Health. 2026 Aug 27;8:1933451. doi: 10.3389/fdgth.2026.1933451 (PMC13558417; doi:10.3389/fdgth.2026.1933451)
Supplement: Supplementary file 2 [file Datasheet2.docx]

**Supplementary File I-b. Google Forms configuration evidence: response settings, consent gate, and implemented branching**

Manuscript 1933451 — *Generative AI use before medical visits: disclosure-item responses, trust, and care-seeking behaviors in a cross-sectional social-media survey in Poland*

**1. Index of screenshots provided with this revision**

| **Screenshot** | **What it documents** | **Reviewer point addressed** |
| --- | --- | --- |
| S1 — Settings panel (“Ustawienia”) | Email collection disabled; post-submission editing disabled; one response per Google account with sign-in required; quiz mode off | Response settings and duplicate-response controls |
| S2 — Section 5 of 14, consent item | Consent gate branching: “Yes, I consent” → next section; “I do not consent” → submit form (survey ends). Item required | Consent procedure; branching evidence |
| S3 — Section 6 of 14, eligibility and attestation | Eight-point attestation with the rule “select at least 8”; item required | Eligibility documentation; required-item settings |
| S4 — Section 7 of 14, item Q1 | Implemented branching of Q1: “Yes” → next section; “No” and “I don’t know / don’t remember” → Section 14 (trust, attitudes, and sociodemographic items). Item required | Q1 routing (skip of Q2–Q14) |
| S5 — Item Q4 | Implemented branching of Q4: “Yes” → next section (Q5–Q9); “No” and “I don’t remember” → Section 10 (actions after obtaining AI information). Item required | Q4 routing (display of Q5–Q9) |
| S6 — Section 10 of 14, item Q11 | Implemented branching of Q11: “Yes” → next section (Q12); “No”, “I don’t remember”, and “prefer not to answer” → Section 12 (informing the physician). Item required | Q11 routing (display of Q12) |
| S7 — Section 12 of 14, item Q13 | Implemented branching of Q13: “No” → next section (Q14); “Yes”, “I don’t remember”, and “not applicable” → Section 14. Item required | Q13 routing (display of Q14) |

The screenshots document the response settings, the consent gate, the required-item configuration, and the implemented branching rules for all four routing items (Q1, Q4, Q11, Q13). They show the form-editing interface only and contain no participant data. The branching rules visible in S4–S7 correspond to the routing specification in Supplementary File P and to the structural non-display (N/D) patterns in the complete response export.

**2. Documented response settings**

| **Setting (Polish interface)** | **English equivalent** | **State** | **Consequence for the dataset** |
| --- | --- | --- | --- |
| Zbieraj adresy e-mail | Collect email addresses | Nie zbieraj (off) | No respondent email addresses were collected or available in the research dataset |
| Wyślij użytkownikom kopie ich odpowiedzi | Send respondents a copy of their responses | Wył. (off) | Not applicable; no email addresses processed |
| Zezwalaj na edytowanie odpowiedzi | Allow response editing after submission | Off | Submitted responses could not be modified after submission |
| Ogranicz do jednej odpowiedzi (wymaga zalogowania) | Limit to 1 response (sign-in required) | On | One response per Google account; respondents had to sign in. Account identifiers were not exported to, or retained in, the research dataset |
| Zrób z tego test | Make this a quiz | Off | No scoring, correct answers, or automatic feedback |

**3. Implemented branching rules as configured in the form**

| **Item** | **Response option** | **Configured destination** | **Consequence** |
| --- | --- | --- | --- |
| Q1 | Yes | Next section (Section 8) | Q2–Q14 displayed |
| Q1 | No; I don’t know / don’t remember | Section 14 | Q2–Q14 not displayed; recorded as N/D (n=164) |
| Q4 | Yes | Next section (Section 9) | Q5–Q9 displayed |
| Q4 | No; I don’t remember | Section 10 | Q5–Q9 not displayed |
| Q11 | Yes | Next section (Section 11) | Q12 displayed |
| Q11 | No; I don’t remember; prefer not to answer | Section 12 | Q12 not displayed |
| Q13 | No | Next section (Section 13) | Q14 displayed |
| Q13 | Yes; I don’t remember; not applicable | Section 14 | Q14 not displayed |
| Consent item (Section 5) | I do not consent | Submit form | Survey terminated |

**4. Author attestation**

Email-address collection was disabled, post-submission editing was not permitted, and submissions were limited to one response per Google account, which required respondents to sign in. The consent item, the eligibility and attestation item, and the four routing items were configured as required, and non-consent terminated the survey. The corresponding author confirms that these settings and branching rules were unchanged during data collection from 1 March to 15 April 2026.

**5. Scope and limitations of this evidence**

The screenshots were taken from the form after the field period and document the configuration as retained in the form; they are supported by the corresponding author’s attestation that the configuration was unchanged during data collection, and they are consistent with the structural non-display patterns observed in the complete response export (all 164 respondents not reporting health-related AI use have N/D on Q2–Q14 and complete responses on Q15–Q24). The sign-in requirement limited duplicate participation at the Google-account level but could not exclude multiple submissions by the same individual using different accounts, and no timestamps, IP addresses, or device identifiers were available in the research dataset.

**6. Screenshot S1. Response settings (“Ustawienia”)**

*Response settings panel of the fielded form (Polish interface).*

**7. Screenshot S2. Consent gate with branching (Section 5 of 14)**

*Consent gate (Section 5 of 14): consent continues to the next section, non-consent submits and terminates the form; item required.*

**8. Screenshot S3. Eligibility and attestation item (Section 6 of 14)**

*Eligibility and attestation item (Section 6 of 14): eight confirmations with the rule "select at least 8"; item required.*

**9. Screenshot S4. Item Q1 with branching (Section 7 of 14)**

*Item Q1 (Section 7 of 14): "Yes" continues to the next section; "No" and "I don’t know / don’t remember" jump to Section 14; item required.*

**10. Screenshot S5. Item Q4 with branching**

*Item Q4: "Yes" continues to the next section (Q5–Q9); "No" and "I don’t remember" jump to Section 10; item required.*

**11. Screenshot S6. Item Q11 with branching (Section 10 of 14)**

*Item Q11 (Section 10 of 14): "Yes" continues to Q12; "No", "I don’t remember", and "prefer not to answer" jump to Section 12; item required.*

**12. Screenshot S7. Item Q13 with branching (Section 12 of 14)**

*Item Q13 (Section 12 of 14): "No" continues to Q14; "Yes", "I don’t remember", and "not applicable" jump to Section 14; item required.*
